# Stereoselective Domino Rearrangement *peri-*Annulation of *Cinchona* Alkaloid Derivatives with 8-Bromo-1-naphthyl Grignard

**DOI:** 10.1021/acs.joc.2c01249

**Published:** 2022-08-23

**Authors:** Przemysław J. Boratyński

**Affiliations:** Department of Organic and Medicinal Chemistry, Wrocław University of Technology, Wyb. Wyspiańskiego 26, Wrocław 50-370, Poland

## Abstract

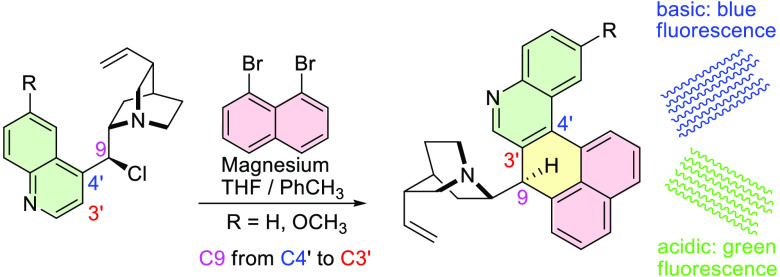

The unexpected domino coupling and rearrangement of the *Cinchona* alkaloid skeleton has been found to occur in the reaction of 9-chloro-9-deoxy-alkaloids with Grignards from *peri*-dihalogenonaphthalene. The cyclization and migration of the central quinuclidinylmethyl group (C9) from position C-4′ to position C-3′ of quinoline formed a novel chiral ring system of 5-aza-7*H*-benzo[*no*]tetraphene, yielding products of *unlike* configuration. The proposed reaction pathway involves radical intermediates.

## Introduction

**Scheme 1:**
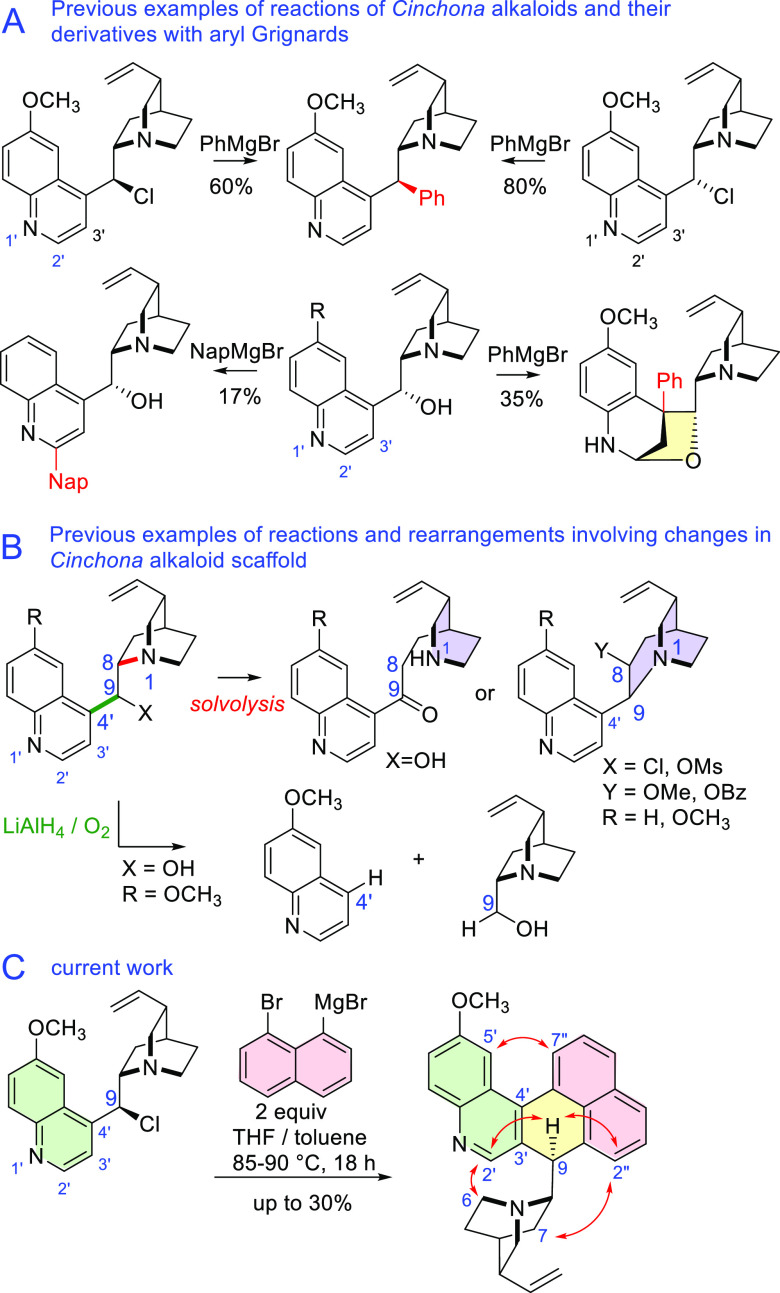
(A) Reactivity of *Cinchona* Alkaloids and Their Derivatives with Grignard Reagents; (B) Rearrangements and Bond Dissociations in *Cinchona* Alkaloid Chemistry Involving the C-9 Atom; (C) New Domino Coupling–Rearrangement; Essential NOESY Interactions and Traditional Atom Numbering Are Shown

The *Cinchona* alkaloids are of relevance to medicinal chemistry and the development of asymmetric catalytic methods. Many valuable organocatalysts and metal ligands were made by the manipulation of the central 9-hydroxyl group of quinine.^1^ In 2008, a synthetically viable substitution of 9-chlorodeoxy-*Cinchona* alkaloids with sp^2^ Grignard reagents providing 9-arylated *Cinchona* derivatives emerged. The reactions proceeded stereoconvergently, producing only an 8,9-*like* diastereoisomer from both 9*R* and 9*S* epimers of chloro derivatives. This stereochemical outcome was justified by the coordination of magnesium by the quinuclidine nitrogen atom.^2^ In previous reactions of Grignard reagents from *meta*- and *para*-dihalobenzene, halophenyl derivatives were rather efficiently prepared (23–80% yield), while di-Grignard reagents produced the corresponding dimeric products.^3^ Reactions of unmodified alkaloids with Grignard reagents have been shown to result in nucleophilic additions at positions 4′ and 2′ (Scheme 1A).^4^

*Cinchona* alkaloids functionalized at position 9 with the 1,2-disubstituted naphthyl group have been utilized in several asymmetric transformations.^5^ The naphthalene ring facilitates π-interactions and gives versatility to functional group placement. Although the most effective spatial control would be enforced through 1,8-substitution (*peri* arrangement),^6^ no such product has been described, hence the attempt to couple a *Cinchona* alkaloid with the 8-functionalized naphthalene ring amenable for further derivatization. The reactivity of 1,8-dihalonaphthalene-derived Grignards with 9-chloro-9-deoxy-quinine resulted in the formation of an additional carbon–carbon bond and an unprecedented rearrangement within the quinoline part. Previously, some modifications at position 9 caused rearrangements of the alkaloid structure, mostly by the breakage of the adjacent N-1–C-8 bond, which resulted in quinuclidine ring opening or ring expansion.^1,7^ There is a reaction of a likely radical mechanism, in which the quinuclidine methyl group separates from the quinoline ring on treatment with the LiAlH_4_/O_2_ system (Scheme 1B).^8^ However, no migration of this group to other positions of the quinoline ring has been reported. Since the migration of any group from position 4 to position 3 of pyridine requires special circumstances, it is rare.^9^

## Results and Discussion

In order to selectively modify *Cinchona* alkaloids with the previously developed method for C-9–C bond formation,^2^ 9*S*-chlorodeoxyquinine was treated with the Grignard reagents obtained separately from 1,8-diiodonaphthalene and 1,8-dibromonaphthalene. The electrospray mass spectrometry of the crude reaction mixtures only revealed traces of naphthalene 8-halogenated derivatives. Instead, the most abundant signal originated from unexpected product **1**, which was isolated in up to 30% yield (Scheme 1C). In this product, the quinuclidine methylene unit (the C-9 atom) migrated from position 4′ to position 3′ of the quinoline ring and the newly introduced naphthalene ring became fused between the central C-9 and quinoline C-4′ carbon atoms. The product contains an unprecedented fusion of five rings with one nitrogen and one sp^3^ carbon atom of defined stereochemistry.

By way of model experiments on the reaction mechanism, the reaction of 1,8-dibromonaphthalene with magnesium in tetrahydrofuran (THF) was initially found to provide a mixture of mono- and bis-Grignard reagents (3:1 to 10:1) after 1 h of reaction time and only moderately correlated with the ratio of reactants. When the reaction was carried out for 18 h, bis-Grignard^10^ and mono-Grignard were both separately prepared in an estimated >92% selectivity by controlling the magnesium to dibromonaphthalene ratio (2.1:1 and 1.07:1) as evidenced by quenching experiments (for details, see the SI). In the subsequent reaction with 9-chloro-9-deoxyquinine, an increase in bis-Grignard quantity led to a significant deterioration of yields. An opposite effect was seen with pure 8-bromo-1-naphthylmagnesium bromide, which provided the highest yield when used in 2-fold excess (30% for 2.0 equiv, compared to 16% for 1.3 equiv). Furthermore, the quantity of isolated product **1** (0.3 mmol) exceeded the content of the initial bis-Grignard species (0.2 mmol).

All 9-chloro-9-deoxy derivatives of *Cinchona* alkaloids reacted in the same manner as quinine (Table 1). Much lower yields (4–5%) were observed for derivatives of cinchonine and cinchonidine, which do not have the 6′-methoxy group. The lack of the methoxy group has been previously shown to halve the yield of the initial coupling at position 9 with simple aryl Grignards.^2^

**Table 1:**
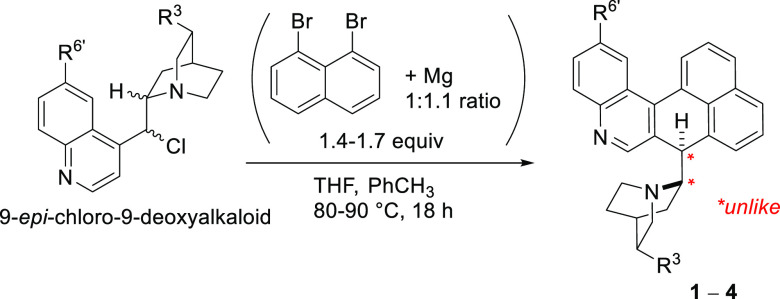
Domino Coupling–Rearrangement of 9-Chloro-9-deoxy *Cinchona* Alkaloids

parent alkaloid	R^6′^	R^3^	product config	product, %^a^
quinine	OMe	C_2_H_3_	(8*S*,9*R*)	**1**, 16–30^b^
quinidine	OMe	C_2_H_3_	(8*R*,9*S*)	**2**, 15
cinchonine	H	C_2_H_3_	(8*R*,9*S*)	**3**, 5
dihydrocinchonidine	H	Et	(8*S*,9*R*)	**4**, 4

a Isolated yield.

b Under optimized conditions with 2 equiv of 8-bromo-1-naphthylmagnesium bromide.

The structures of quinine-derived **1** and cinchonine-derived **3** were unambiguously elucidated from NMR experiments (see the SI). The relative configuration of the products was investigated with a combination of NMR and density functional theory (DFT) computations. In the lowest energy conformers of quinine-derived products of 9*R* and 9*S* configuration, the observed contacts between atoms of quinuclidine and benzo[*no*]tetraphene rings (Scheme 1C) correspond to 2.2–2.5 Å and 3.7–4.8 Å, respectively. The correlation of computed and experimental chemical shifts is also noticeably better assuming *unlike* (8*S*,9*R*)-**1** and *unlike* (8*R*,9*S*)-**3** configurations (for details, see the SI). Therefore, for the isolated products, rearrangement must have occurred with the inversion of configuration. Flash chromatography aided by mass spectrometry detection revealed traces of plausible isomeric products. These isomers were not isolated in pure form; nevertheless, the diastereomeric ratio lower limit was estimated at 20:1. The reaction of any 9 epimer of 9-chlorodeoxyquinine resulted in the formation of the same isomer of product **1**. This is partly consistent with our previous finding that the Wurtz-type coupling of Grignard reagents only produced a single *like* stereoisomer of the product regardless of the configuration at position 9 of the starting material.^2^ In later experiments it was shown that thermodynamic base-promoted equilibration produced a mixture of stereoisomers in comparable quantities.^11^ Here, for the reaction quenched in D_2_O no observable incorporation of deuterium into the molecule occurred thus precluding thermodynamic equilibration of the product.

**Scheme 2:**
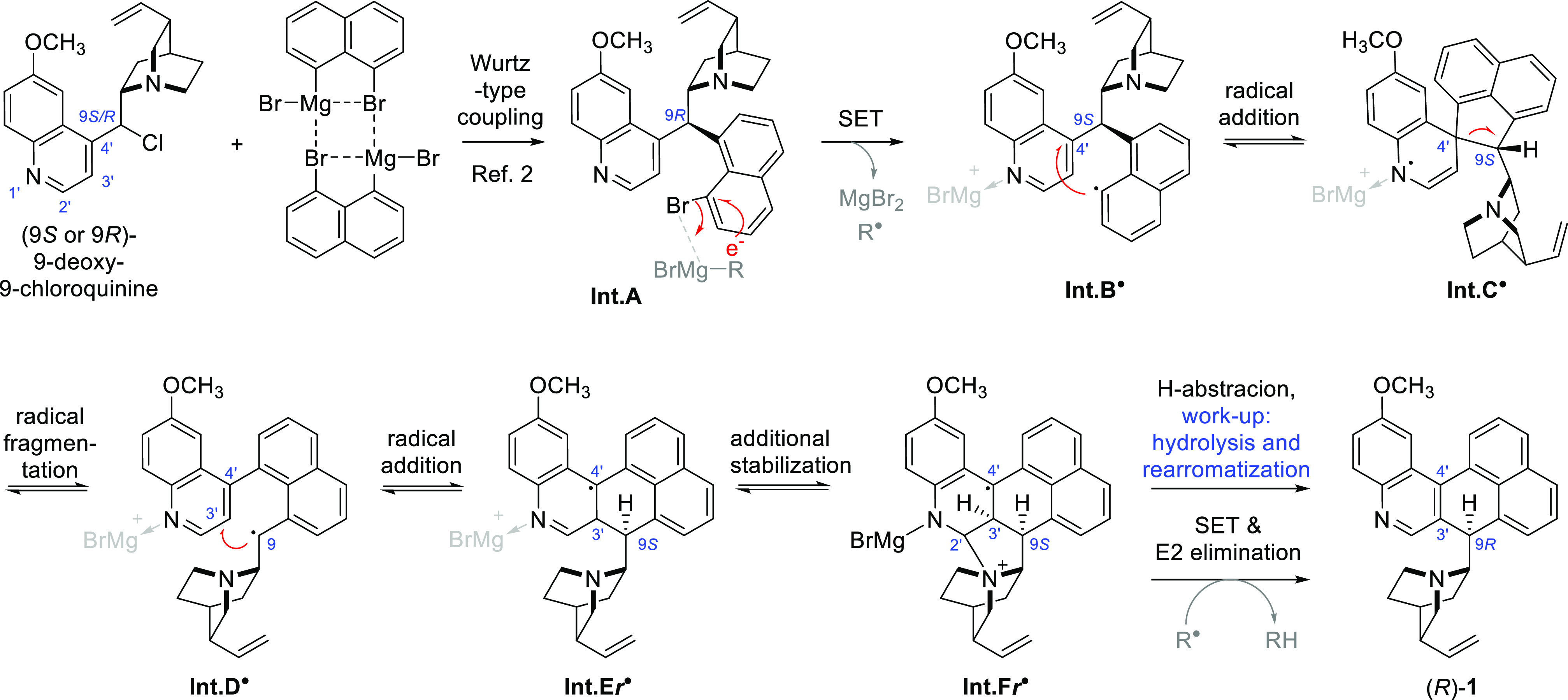
Outline of Considered Intermediates in the Observed Substitution–Rearrangement Reaction

A tentative reaction mechanism can be outlined (Scheme 2). First, the substitution of quinine 9-halide with 8-bromonaphth-1-ylmagnesium bromide according to the previously described pathway^2^ would produce intermediate **Int.A**. Proximity of another Grignard molecule could initiate single electron transfer (SET) analogous to the one postulated for metal–halogen exchange in main group organometallic chemistry, particularly at elevated temperatures.^12^ This could produce aryl radical **Int.B**^**•**^.^13^ This localized nucleophilic radical can attack the C-4′ atom of the quinoline ring, forming a spirocyclic radical **Int.C**^**•**^.^14^ The ensuing fragmentation of the C-4′/C-9 bond produces **Int.D**^**•**^ in an overall radical substitution reaction from **Int.B**^**•**^.^15^ In this intermediate, the radical is of a highly delocalized benzyl type and as such is expected to be more stable than **Int.B**^**•**^. Furthermore, sufficient lifetime of this species may result in the loss of stereochemistry at the sp^2^ carbon at the former position 9. Productive intramolecular addition^16^ in the intermediate **Int.D**^**•**^ can result in the formation of a bond between C-9 and quinoline C-3′, giving diarylmethyl-type radical **Int.E**^**•**^, which has a complete carbon skeleton of the end product **1**. A similar mechanism was proposed for radical rearrangement annulation involving nitrogen-centered (aminyl) attacking and leaving radicals.^17^ Overall, the presence of a radical pathway is partly supported by the observation of a faint EPR signal after 3 h of reaction time and by the trapping experiments with DMPO and TEMPO. The adduct with DMPO showed an intense EPR trace, which could not be easily interpreted. The diamagnetic coupling product with TEMPO was identifiable in the ESI-MS. The observed value (*m*/*z* 590) is consistent with the formula of isomeric intermediate radicals **Int.B–F**^**•**^ (for details, see the SI). The end radical **Int.E**/**Int.F**^**•**^ will eventually become diamagnetic **1**, either by hydrogen abstraction and oxidative rearomatization during workup or by electron abstraction in another SET process. The latter explanation may be consistent with the unchanged ESI-MS spectral pattern following the workup of the reaction mixture under reductive conditions (NaBH_4_). For a brief discussion of alternative reaction pathways, see the SI.

**Figure 1:**
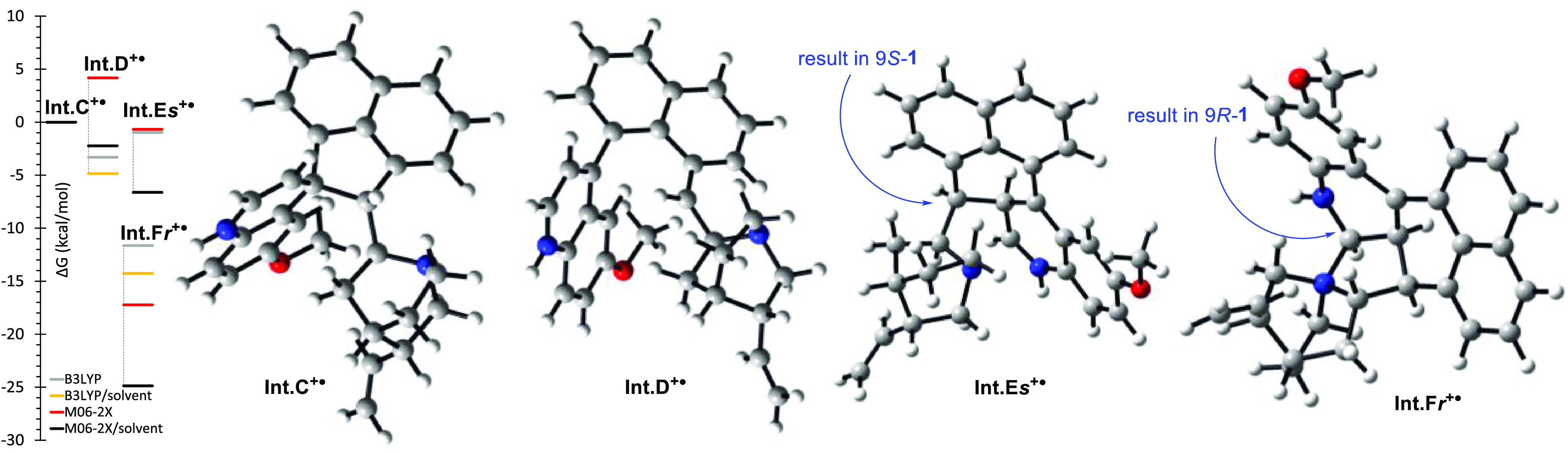
Computed ground state free energies DFT/B3LYP or M06-2X levels of theory (gas phase and SMD solvent model for THF) with the CC-pVDZ basis set and structures and at the SMD/M06-2X/CC-pVDZ level of intermediate radical cation models of **Int.C**^**+**^^•^, **Int.D**^**+**^^•^, and **Int.Es/F*****r***^**+**•^.

Some stabilization of the proposed intermediates **Int.C–F**^**•**^ may be offered by forming a coordination bond between the quinoline nitrogen atom and magnesium ions.^12,18^ DFT calculations on simplified models (MgBr^+^ removed or replaced with a proton) were conducted at the DFT/B3LYP/CC-pVDZ and M06-2X/CC-pVDZ levels of theory. These indicate that the radical isomerization pathway from **Int.B**^**•**^ to **Int.F**^**•**^ is energetically favorable. For the observed (9*R*)-**1**, additional stabilization can be offered by the interaction between quinoline C-2′ and quinuclidine nitrogen atoms (**Int.F*****r***^**•**^). In the radical cation model, the geometry of **Int.E*****r***^**•**^ converges into **Int.F*****r***^**•**^. This intermolecular nucleophilic addition is spatially not accessible for the unobserved 9*S* epimer of **1** and is likely the cause of the observed stereoselectivity in the reaction (Figure 1).

Attempts to extend the scope of the reaction for either other *peri*-substituted arenes or non-*Cinchona* alkaloid derivatives were synthetically ineffective. For the reaction of similar 5,6-dibromoacenaphthene, the most abundant signal in the mass spectrometry corresponded to alkaloid 9-dimer. The presence of a likely cyclized product was evident (*m*/*z* = 459), but the quantity was low and the isolation of a sample of sufficient purity was not achieved. The reaction of organomagnesium compound from 1,8-dibromonaphthalene and 4-quinoline carbaldehyde was also attempted. Again, no annulation–rearrangement product was received; however the relevant ESI-MS (*m*/*z* = 284) signal was observed.

**Figure 2:**
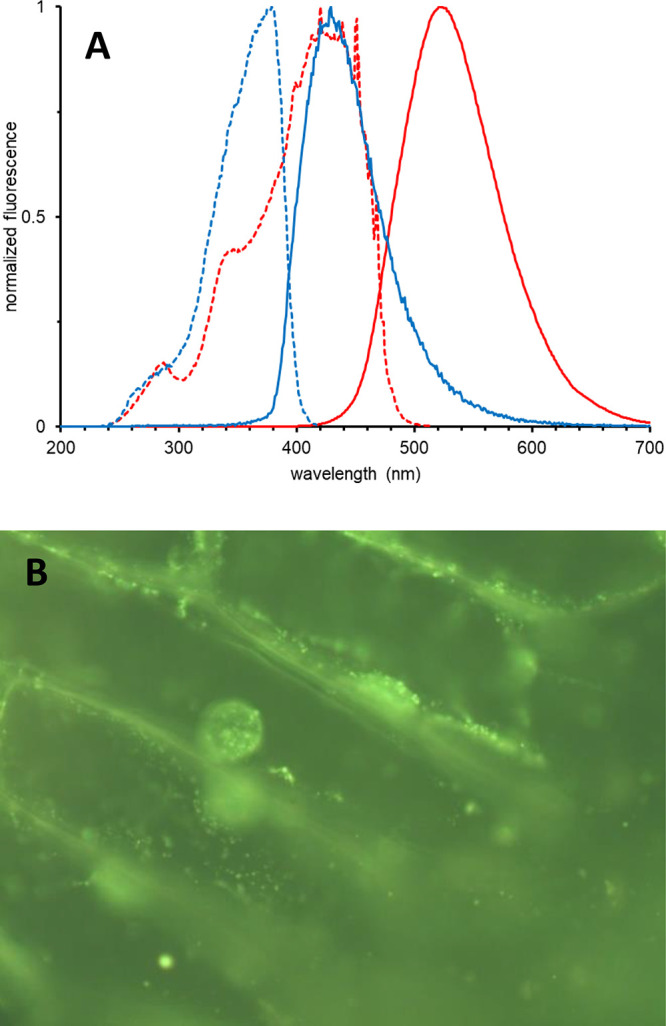
(A) Emission (solid lines) and excitation (dashed lines) for a 10^–4^ M solution of **1** in DCM without acid (blue lines) and in 15 mM TFA (red lines). (B) Fluorescence micrograph (280× magnification, FITC setup) of live plant skin from *Allium cepa* stained with **1**.

The obtained products can be defined as nitrogen-containing polyaromatics which may be valued for their electronic and associated fluorescent properties.^19^ The large nearly planar polycyclic aromatic system with a nitrogen atom in **1**–**4** is the cause of fluorescent properties on the TLC plate and in the solution. In the absence of external acid, blue light is emitted, while in 15 mM TFA the solution of **1** becomes deeply orange and green fluorescence emerges with a similar quantum yield (Φ_F_ = 0.32–0.45, Figure 2A). The corresponding emission maxima for quinine-derived **1** are 436 nm for neutral and 522 nm for acidic samples. The presence of acid also increases the Stokes shift by a factor of 2 (56 nm vs 94 nm). In contrast, the results for the cinchonine derivative **3** (Φ_F_ = 0.47–0.58) show that the methoxy group is not responsible for fluorescence (for details, see the SI). The structure of the modified natural products as well as acidity-dependent fluorescence prompted the evaluation of its utility for biological staining in a simple assay.^20^ The microscopic live plant cell imaging with the quinine derivative **1** revealed preferential fluorescence staining of some globular cell cytoplasm structures surrounding the nucleus and cell walls (Figure 2B).

## Conclusions

In summary, the unprecedented rearrangement involving carbon bond migration from position 4 to position 3 of quinoline without transition metals and under nonacidic conditions forms a novel chiral 5-aza-7*H*-benzo[*no*]tetraphene ring system with fluorescent properties. While the isolated products are limited to *Cinchona* alkaloid derivatives, the transformation may be relevant to other lepidine and *peri*-naphthalene derivatives.

## Experimental Section

### General Comments

NMR spectra were collected on a 600 MHz Bruker Avance II instrument. Spectra were internally referenced to tetramethylsilane (TMS, δ_C_ = 0 and δ_H_ = 0). Structural assignments were made with additional information from gCOSY, gHSQC, gHMBC, and NOESY experiments. Electrospray (ESI) MS and HRMS spectra were recorded on a Waters LCT Premier XE apparatus with a TOF analyzer. ECD spectra were measured on a Jasco J-1500 circular dichroism spectrophotometer. UV–vis spectra were taken on a Jasco V-670 spectrophotometer. Fluorescence spectra were taken on a Horiba Fluoromax-4 spectrofluorimeter and are uncorrected. Flash chromatography was performed on standard silica gel 230–400 mesh (Merck). Automated flash-chromatography system CombiFlash NextGen 300 (ISCO, Teledyne) was used in some isolations. TLC plates with F256 indicator (Sigma-Aldrich) were illuminated by a dual UV lamp at 256 and 365 nm. *Cinchona* alkaloids were purchased from Buchler (Braunschweig, Germany). 9*S*-Chloro-9-deoxyquinine and other 9-deoxy-9-halogeno-alkaloids were obtained by the treatment of the corresponding *Cinchona* alkaloid with thionyl chloride (56–84% yield) as described in the literature.^21^ THF was purified and dried by sequential distillation from LiAlH_4_ and distillation from sodium/benzophenone, and toluene was dried by storing over sodium chunks. All other reagents were purchased from commercial suppliers (Merck/Sigma-Aldrich and Fluorochem) and used as received.

### (7*R*)-5-Aza-2-methoxy-7-((1*S*,2*S*,5*R*)-5-vinylquinuclidin-2-yl)-7*H*-benzo[*no*]tetraphene (**1**); Quinine Derivative **1**

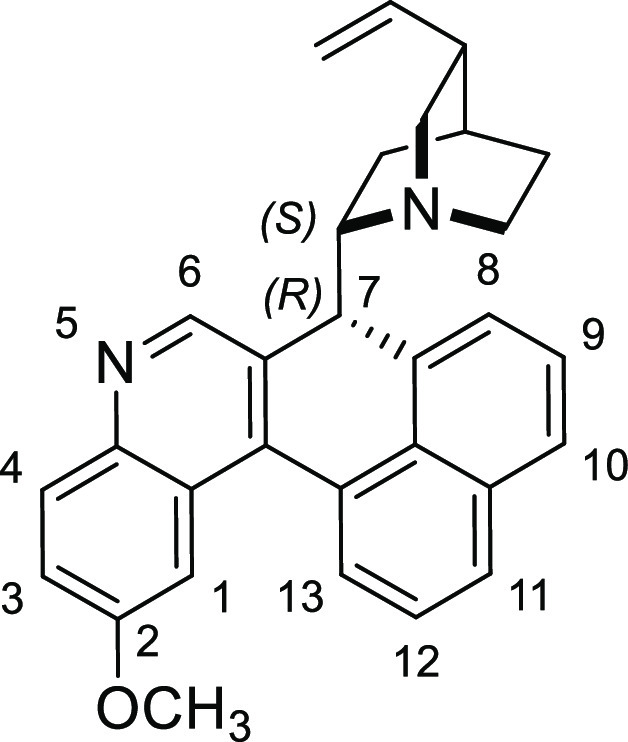



Magnesium (190 mg, 7.88 mmol, 1.5 equiv) was activated with iodine (ca. 10 mg) and suspended in dry THF (26 mL) under argon. 1,8-Dibromonaphthalene (2.08 g, 7.26 mmol, 1.4 equiv) was added, and the mixture was stirred under reflux in an oil bath. 1,2-Dibromoethane was added in small portions (total 50 μL, 0.58 mmol, 0.1 equiv), and after ca. 1–1.5 h almost all magnesium dissolved. Then, a solution of 9*S*-chloro-9-deoxyquinine (1.78 g, 5.19 mmol, 1 equiv) in toluene (25 mL) was added to the reaction mixture, and stirring was continued in an oil bath set at 85–90 °C for 18 h. The mixture gradually took a deep brown color. The heating was discontinued, and at room temperature the reaction was quenched with a saturated NH_4_Cl solution (15 mL), extracted with DCM (1 × 60 mL, 3 × 10 mL), and dried over MgSO_4_ in a flask open to air. After 18 h the mixture was filtered, evaporated, and subjected to column chromatography on silica gel with DCM/MeOH (3% to 5% gradient), and fractions containing a bright fluorescent spot on TLC were collected. Obtained 0.449 g of **1** as a light brown, amorphous solid (20%).

Repeated reactions on 0.6–3.5 g (2–10 mmol) scale gave 15–20% yields. The reaction performed with 1,8-diiodonaphthalene instead of 1,8-dibromonaphthalene gave a 9% yield. An increase of relative quantity of magnesium vs 1,8-dibromonaphthalene or use of >2 equiv of magnesium generally caused a decrease in isolated pure product yield. Reactions run for significantly shorter time (3 h) or at room temperature showed much lower conversion. Similar yields were obtained for the reactions run in benzene/THF instead of toluene/THF. A reaction performed with 2.0 equiv of 8-bromo-1-naphthylmagnesium bromide gave a 30% yield.

### Preparation of 8-Bromo-1-naphthylmagnesium Bromide

In a sealable Schlenk tube under an argon atmosphere magnesium (55 mg, 2.28 mmol, 1.07 equiv) was placed and activated with bromine (ca. 20 μL) at 150–200 °C for 3 min, bromine was evacuated, and a solution of 1,8-dibromonaphthalene (664 mg, 2.32 mmol) in dry THF was added (10 mL). The container was sealed and heated at 80 °C in an oil bath for 18 h, producing a dark brown but transparent solution, which was used directly for the preparation of **1** (26–30% yield). ^1^H NMR (600 MHz, CDCl_3_, TMS): δ 8.75 (s, 1H), 8.44 (d, *J* = 7.2 Hz, 1H), 8.05 (d, *J* = 9.1 Hz, 1H), 8.01 (d, *J* = 2.6 Hz, 1H), 7.92 (d, *J* = 8.2 Hz, 1H), 7.76–7.79 (m, 1H), 7.68 (t, *J* = 7.7 Hz, 1H), 7.47–7.49 (m, 2H), 7.32 (dd, *J* = 9.1, 2.6 Hz, 1H), 5.33 (ddd, *J* = 17.1 10.3, 8.1 Hz, 1H), 4.72 (dt, *J* = 17.1, 1.1 Hz, 1H), 4.64 (dt, *J* = 10.3, 1.5 Hz, 1H), 4.19 (d, *J* = 10.1 Hz, 1H), 3.92 (s, 3H), 3.37–3.43 (m, 1H), 2.94 (dd, *J* = 14.1, 9.9 Hz, 1H), 2.82–2.88 (m, 1H), 2.32 (q, *J* = 9.5 Hz, 1H), 2.19 (dt, *J* = 14.1, 2.9 Hz, 1H), 2.03–2.07 (m, 1H), 1.58–1.63 (m, 2H), 1.45–1.52 (m, 1H), 1.07–1.12 (m, 1H), 0.73–0.79 (m, 1H) ppm. ^1^H NMR (600 MHz, C_6_D_6_): δ 9.01 (s, 1H), 8.36 (d, *J* = 9.0 Hz, 1H), 8.31 (d, *J* = 7.3 Hz, 1H), 8.02 (d, *J* = 2.7 Hz, 1H), 7.62 (d, *J* = 8.4 Hz, 1H), 7.60 (d, *J* = 8.7 Hz, 1H), 7.33 (dd, *J* = 8.0, 7.7 Hz, 1H), 7.31 (t, *J* = 7.8 Hz, 1H), 7.24 (dd, *J* = 9.0, 2.7 Hz, 1H), 7.20 (d, *J* = 7.0 Hz, 1H), 5.02 (ddd, *J* = 17.2 10.4, 8.7 Hz, 1H), 4.57 (dt, *J* = 17.2, 1.5 Hz, 1H), 4.41 (dt, *J* = 10.4, 1.4 Hz, 1H), 3.93 (d, *J* = 10.1 Hz, 1H), 3.38 (s, 3H), 3.02–3.08 (m, 1H), 2.74 (dd, *J* = 14.0, 10.0 Hz, 1H), 2.52–2.57 (m, 1H), 2.50 (q, *J* = 9.4 Hz, 1H), 2.17 (dt, *J* = 14.0, 2.9 Hz, 1H), 1.72–1.77 (m, 1H), 1.35–1.38 (m, 1H), 1.25–1.34 (m, 1H), 1.08–1.15 (m, 1H), 0.89–0.94 (m, 1H), 0.74–0.80 (m, 1H) ppm. ^13^C{^1^H} NMR (151 MHz, CDCl_3_, TMS): δ 158.0, 150.3, 144.4, 142.4, 134.5, 133.38, 133.29, 132.6, 131.7, 130.4, 128.8, 128.6, 126.14, 126.02, 126.00, 125.95, 125.5, 125.2, 119.4, 113.8, 104.9, 62.8, 56.6, 55.5, 47.2, 41.3, 40.5, 28.7, 28.2, 27.7 ppm. ^13^C{^1^H} NMR (151 MHz, C_6_D_6_): δ 158.1, 150.7, 145.3, 142.1, 133.83, 133.79, 133.57, 132.8, 132.5, 130.6, 129.1, 128.5, 126.1, 125.98, 125.92, 125.87, 125.3, 125.1, 119.3, 113.4, 104.9, 63.0, 56.5, 54.6, 47.0, 41.0, 40.4, 28.5, 28.3, 27.8 ppm. HRMS (ESI-TOF) *m*/*z*: calcd for [C_30_H_28_N_2_O + H]^+^ 433.2274, found 433.2275.

### (7*S*)-5-Aza-2-methoxy-7-((1*S*,2*R*,5*R*)-5-vinylquinuclidin-2-yl)-7*H*-benzo[*no*]tetraphene (**2**); Quinidine Derivative **2**

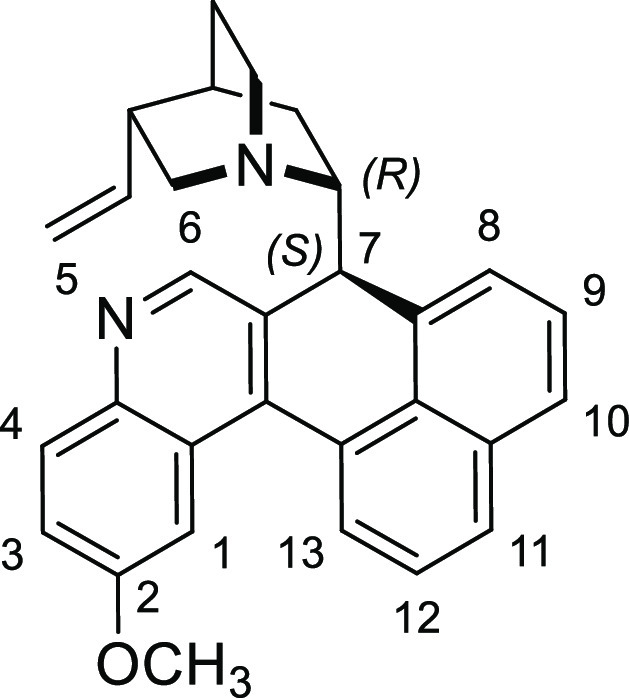



The product was obtained as described for **1** starting from magnesium (0.202 g, 8.38 mmol, 1.9 equiv), 1,8-dibromonaphthalene (2.09 g, 7.30 mmol, 1.7 equiv), and 9*R*-chloro-9-deoxy-quinindine (1.47 g, 4.30 mmol, 1 equiv) instead of chlorodeoxyquinine. Chromatography on silica gel with DCM/MeOH (3% to 5% gradient) gave a brown amorphous solid (277 mg, 15%). ^1^H NMR (151 MHz, CDCl_3_, TMS): δ 8.76 (s, 1H), 8.38 (d, *J* = 7.1 Hz, 1H), 8.05 (d, *J* = 9.2 Hz, 1H), 7.95 (d, *J* = 2.1 Hz, 1H), 7.87 (d, *J* = 8.2 Hz, 1H), 7.75 (d, *J* = 8.1 Hz, 1H), 7.63 (t, *J* = 7.7 Hz, 1H), 7.57 (d, *J* = 6.8 Hz, 1H), 7.46 (t, *J* = 7.5 Hz, 1H), 7.29 (dd, *J* = 9.2, 2.1 Hz, 1H), 5.69 (ddd, *J* = 17.2, 10.1, 7.2 Hz, 1H), 5.00 (d, *J* = 10.1 Hz, 1H), 4.92 (d, *J* = 17.2 Hz, 1H), 4.41 (d, *J* = 8.9 Hz, 1H), 3.87 (s, 3H), 3.01 (dd, *J* = 13.3, 10.2 Hz, 1H), 2.71–2.76 (m, 1H), 2.61–2.68 (m, 1H), 2.50–2.59 (m, 2H), 2.11–2.17 (m, 1H), 1.49–1.52 (m, 1H), 1.26–1.33 (m, 2H), 1.14–1.20 (m, 1H), 0.49–0.55 (m, 1H) ppm. ^13^C{^1^H} NMR (151 MHz, CDCl_3_, TMS): δ 158.0, 149.9, 144.2, 139.6, 135.0, 133.3, 132.5, 131.8, 131.4, 130.5, 128.9, 128.6, 126.2 (2C overlap), 125.94, 125.81, 125.77, 125.2, 119.5, 114.6, 104.8, 62.8, 55.4, 49.3, 47.7, 46.1, 39.4, 28.1, 25.6, 25.4 ppm of HRMS (ESI-TOF) *m*/*z*: calcd. for [C_30_H_28_N_2_O + H]^+^ 433.2274, found 433.2281.

### (7*S*)-5-Aza-7-((1*S*,2*R*,5*R*)-5-vinylquinuclidin-2-yl)-7*H*-benzo[*no*]tetraphene (**3**); Cinchonine Derivative **3**

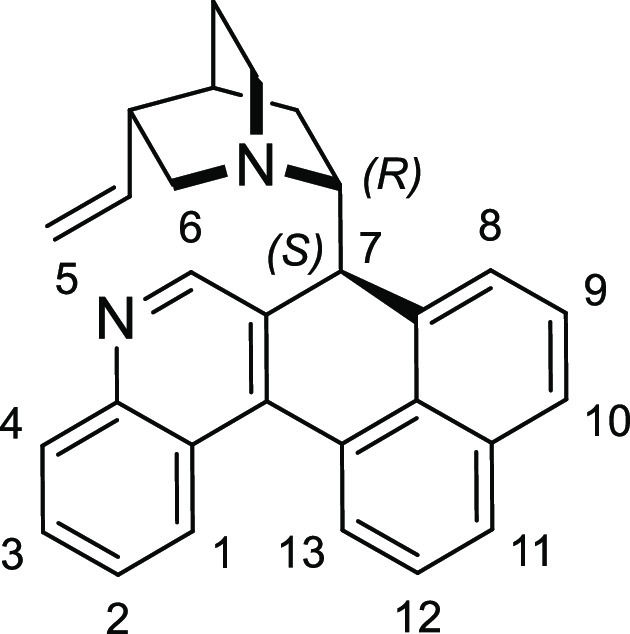



The product was obtained as described for **1** starting from magnesium (0.198 g, 8.22 mmol, 1.5 equiv), 1,8-dibromonaphthalene (2.21 g, 7.72 mmol, 1.4 equiv), and 9*R*-chloro-9-deoxycinchonine (1.74 g, 5.49 mmol, 1 mmol) instead of chlorodeoxyquinine. Chromatography on silica gel with DCM/MeOH 3% to 6% gradient gave a brown amorphous solid (121 mg, 5%). ^1^H NMR (600 MHz, CDCl_3_, TMS): δ 8.89 (s, 1H), 8.61 (d, *J* = 8.7 Hz, 1H), 8.39 (d, *J* = 7.3 Hz, 1H), 8.15 (dd, *J* = 8.7, 1.1 Hz, 1H), 7.93 (d, *J* = 8.2 Hz, 1H), 7.79 (d, *J* = 8.1 Hz, 1H), 7.68 (t, *J* = 7.9 Hz, 1H), 7.63–7.67 (m, 1H), 7.48–7.55 (m, 3H), 5.87 (ddd, *J* = 17.3, 10.4, 7.3 Hz, 1H), 5.08 (dt, *J* = 10.4, 1.4 Hz, 1H), 5.04 (d, *J* = 17.3 Hz, 1H), 4.33 (d, *J* = 9.6 Hz, 1H), 3.06 (dd, *J* = 13.6, 10.1 Hz, 1H), 2.91–2.96 (m, 1H), 2.60–2.67 (m, 1H), 2.42–2.48 (m, 1H), 2.38 (q, *J* = 9.0 Hz, 1H), 2.20 (q, *J* = 8.2 Hz, 1H), 1.55–1.58 (m, 1H), 1.36–1.41 (m, 1H), 1.28–1.34 (m, 1H), 1.12–1.19 (m, 1H), 0.47–0.52 (m, 1H) ppm. ^13^C{^1^H} NMR (151 MHz, CDCl_3_, TMS): δ 152.6, 148.3, 140.3, 135.9, 133.4, 133.0, 131.9, 130.41, 130.26, 129.1, 128.3, 128.0, 126.8, 126.4, 126.21, 126.06, 125.7, 125.5, 125.3, 125.0, 114.5, 62.8, 49.4, 47.9, 46.4, 39.9, 28.3, 26.12, 26.03 ppm. HRMS (ESI-TOF) *m*/*z*: calcd for [C_29_H_26_N_2_ + H]^+^ 403.2169, found 403.2175.

### (7*R*)-5-Aza-7-((1*S*,2*S*,5*R*)-5-ethylquinuclidin-2-yl)-7*H*-benzo[*no*]tetraphene (**4**); Dihydrocinchonidine Derivative **4**

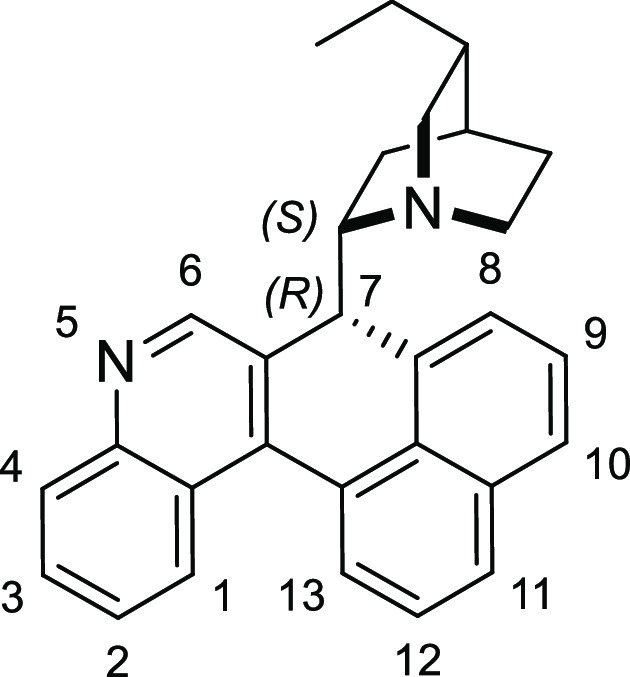



The product was obtained as described for **1** starting from magnesium (0.105 g, 4.36 mmol, 1.7 equiv), 1,8-dibromonaphthalene (0.995 g, 3.48 mmol, 1.4 equiv), and 9*S*-chloro-9-deoxy-10,11-dihydrocinchonidine (0.785 g, 2.49 mmol, 1 mmol) instead of chlorodeoxyquinine. Following chromatography on silica gel with DCM/MeOH (20:1) a light brown, amorphous solid (49 mg, 4%) was obtained. ^1^H NMR (600 MHz, CDCl_3_, TMS): δ 8.89 (s, 1H), 8.60 (d, *J* = 8.6 Hz, 1H), 8.40 (d, *J* = 7.2 Hz, 1H), 8.14 (d, *J* = 8.4 Hz, 1H), 7.92 (d, *J* = 8.1 Hz, 1H), 7.78 (d, *J* = 8.4 Hz, 1H), 7.67 (t, *J* = 7.7 Hz, 1H), 7.63 (ddd, *J* = 8.2, 7.2, 1.1 Hz, 1H), 7.47–7.55 (m, 3H), 4.28 (br., 1H), 3.34 (br., 1H), 2.92 (dd, *J* = 13.6, 9.7 Hz, 1H), 2.84 (ddd, *J* = 13.6, 10.5, 2.7 Hz, 1H), 2.40 (br, 1H), 2.01 (br d, *J* = 13.6 Hz, 1H), 1.56–1.61 (br, 2H), 1.38–1.43 (m, 1H), 1.18–1.29 (m, 3H), 1.06 (dd, *J* = 13.7, 8.9 Hz, 1H), 0.82–0.93 (m, 2H), 0.71–0.77 (m, 1H), 0.59 (t, *J* = 7.4 Hz, 1H) ppm. ^13^C{^1^H} NMR (151 MHz, CDCl_3_, TMS): δ 152.4, 148.4, 135.8, 133.3, 133.0, 132.1, 130.4, 130.3, 129.1, 128.2, 127.9, 126.9, 126.4, 126.2 (2C overlap), 125.56, 125.51, 125.35, 125.1, 62.7, 57.7, 46.9, 41.5, 37.7, 29.1, 27.6, 27.0, 25.6, 12.0 ppm. HRMS (ESI-TOF) *m*/*z*: calcd for [C_29_H_28_N_2_ + H]^+^ 405.2325, found 405.2334.

## Supporting Information Available

Plots of NMR and EPR spectra, assignment of NMR signals for compounds **1** and **3**, plots of ESI-MS, UV–vis, CD, and fluorescence spectra, micrographs, and computational details (PDF)
FAIR data, including the primary NMR FID files, for compounds **1**–**4** (ZIP)
