# Supplementary material for: Stereoselective Domino Rearrangement peri-Annulation of Cinchona Alkaloid Derivatives with 8-Bromo-1-naphthyl Grignard
Source: J Org Chem. 2022 Aug 23;87(17):11602–7. doi: 10.1021/acs.joc.2c01249 (PMC9442652; doi:10.1021/acs.joc.2c01249)
Supplement: Supplementary file 1 — jo2c01249_si_001.pdf [file jo2c01249_si_001.pdf]

Supporting Information for:

Stereoselective domino rearrangement *peri*-annulation of *Cinchona* alkaloid derivatives with 8-bromo-1-naphthyl Grignard

*Przemysław J. Boratyński**
Department of Organic and Medicinal Chemistry, Wrocław University of Technology,
Wyb. Wyspiańskiego 27, Wrocław 50-370 Poland.
e-mail: przemyslaw.boratynski@pwr.edu.pl

Table of Contents

S1. Atom numbering schemes S-2
S2. Spectral assignment for **1**, **3**, and assignment of relative configuration. S-2
S3. Attempts to identify radicals in the reaction mixture S-10
S4. Assays of Grignard reagents S-12
S5. Peripheral discussion of alternative reaction mechanisms S-14
S6. Plots of ESI-MS spectra S-18
S7. UV-Vis absorption and CD spectroscopy S-25
S8. Fluorescence spectroscopy S-27
S9. Microscopic staining experiments S-31
S10. Plots of NMR spectra S-33
S11. Computational details S-46

S1. Atom numbering schemes

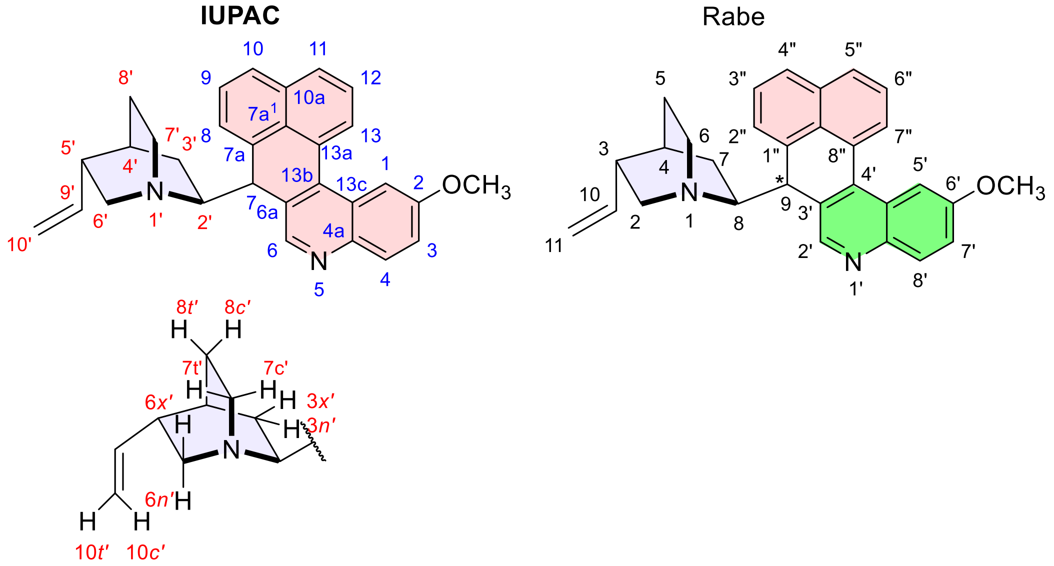

Figure S1. Atom numbering according to IUPAC (left) and traditional atom numbering of cinchonan substrates (right)

S2. Spectral assignment for **1**, **3**, and assignment of relative configuration.

In the NMR spectrum of **1**, the signal of H3' (expected 7.1-7.7 ppm, d, *J* = ca. 4.5 Hz) disappeared and H2' became a singlet (ca. 8 ppm). Initially, it was suspected that cyclization occurred without rearrangement by formation of a carbon bond between C8 of naphthalene and C3' of the quinoline ring because both were quaternary atoms (structure **1a**, Figure S2). This has been ruled out by experimental evidence. NOESY experiments revealed the proximity of H9 atom to H2' of the quinoline ring, and one of the of the beta hydrogens (H2") of the naphthalene ring, while the other beta hydrogen atom (H7") displayed contact with H5' of the quinoline. Should there be no rearrangement and should C4' and C9 atoms remain connected, the interactions between H2' and H9 as well as H7" and H-5' would not be observed in **1a** (Figure S2). The C4 to C3 rearranged connectivity was proven in $^{1}H,^{13}C$ HMBC experiments (Figures S3-S5).

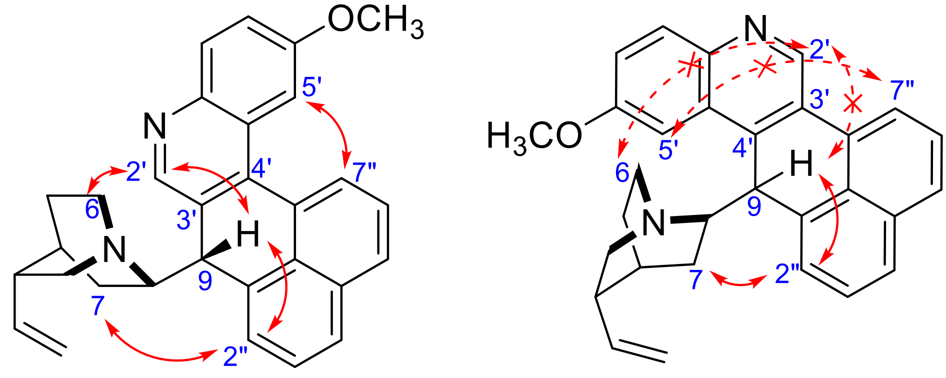

1 (valid structure) **1a** (invalid structure)

Figure S2. Diagnostic NOESY interactions for initially considered products **1** and **1a** prior to structure elucidation. Impossible interactions are drawn with dashed lines, traditional *Cinchona* alkaloid atom numbering is used

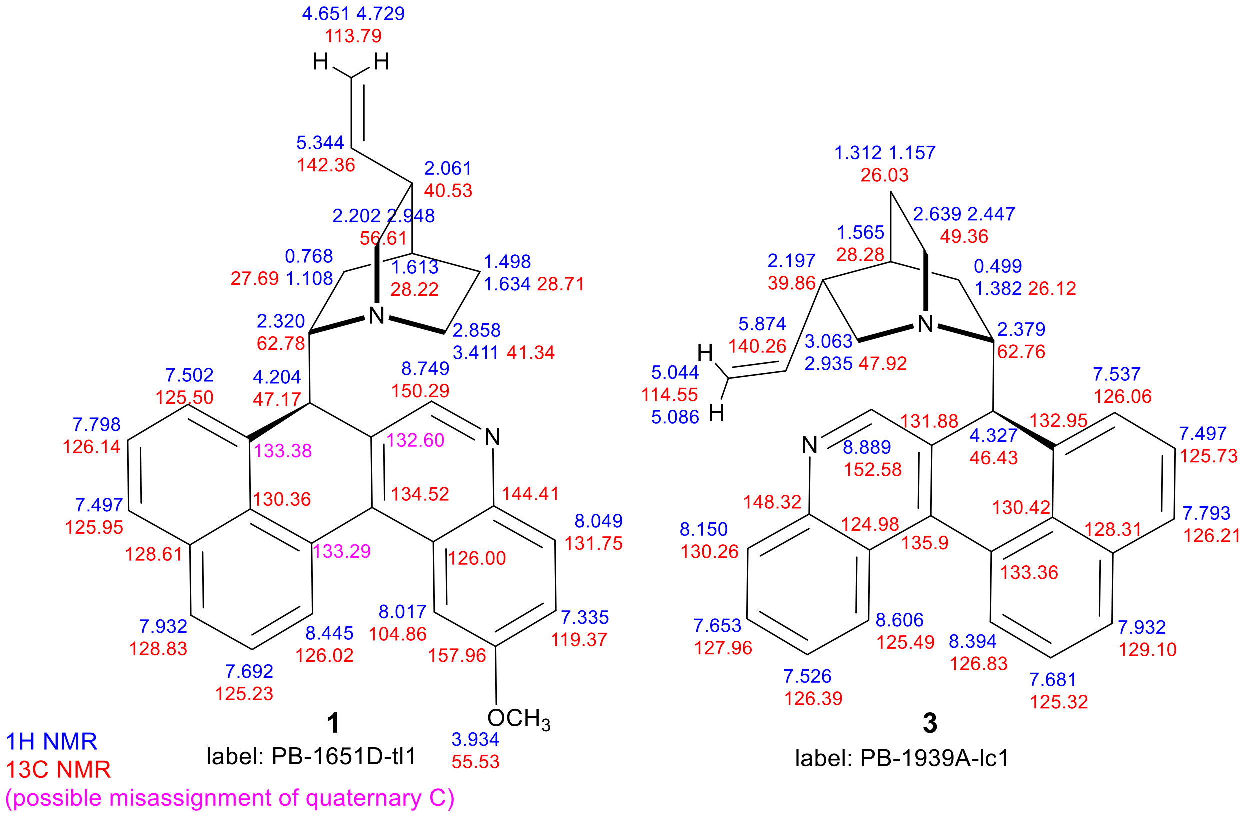

Figure S3. Assignment of ^{1}H (blue) and ^{13}C (red/magenta) NMR signals in $CDCl_3$ for **1** (left) and **3** (right). Up/down and left/right orientation of labels correspond to the assigned diastereotopic protons. For some signals of **1** corresponding to quaternary carbon atoms within 132.6−133.4 ppm, the assignment is tentative due to congestion (magenta). Assignment is based on COSY, HSQC, HMBC, and NOESY experiments. For **1** sample label: PB-1651D, see Figures S30-S32, and Table S1. For **3** sample label: PB-1939A, see Figures S36-S38, and Table S3.

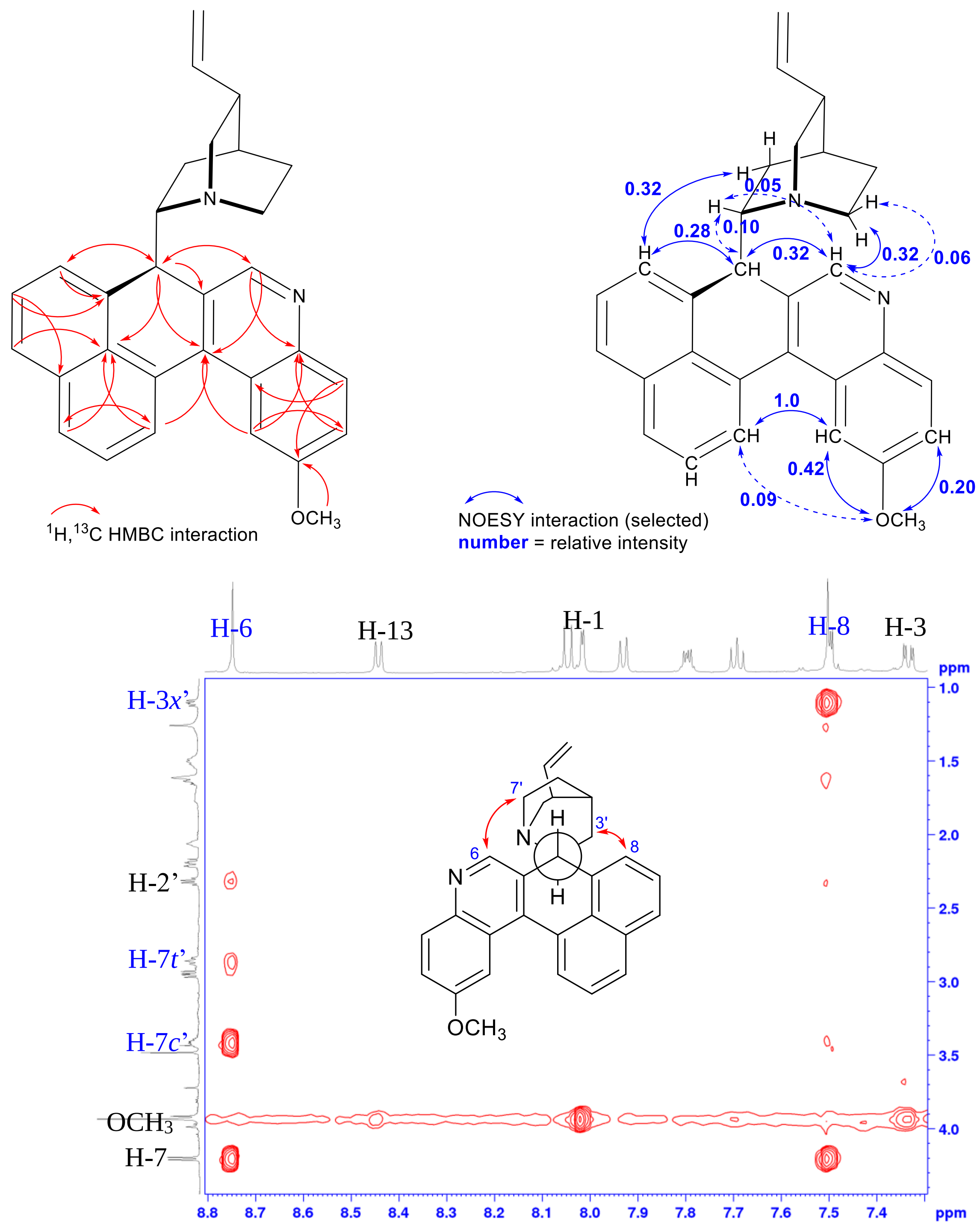

Figure S4. Selected HMBC (top left) and NOESY interactions and their relative integral intensities (top right), and 7.3-8.8 vs. 1.0-4.5 ppm expansion of NOESY spectra for **1** in $CDCl_3$, and relevant Newman projection (bottom). IUPAC atom numbering is used, see Figure S1 for reference. For spectra assignment, see Figure For complete sets of NMR spectra, see Figures S30-S32.

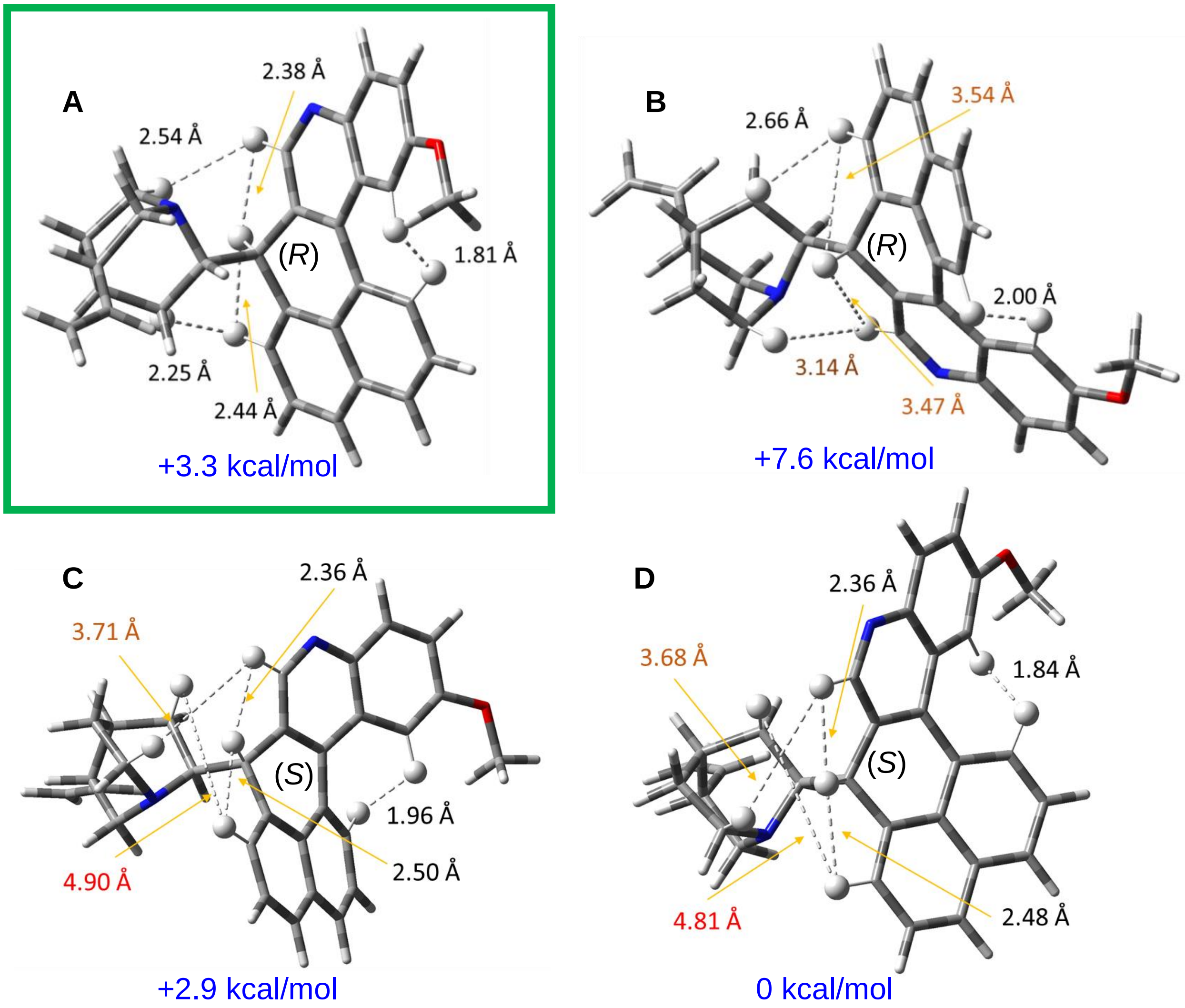

Figure S5. DFT/B3-LYP/CC-pVDZ computed lowest energy geometries of *unlike* (7*R*)-**1**: conformer *P* (Panel **A**), conformer *M* (Panel **B**), and not observed isomer *like* (7*S*)-**1** conformer *P* (Panel **C**) and conformer *M*. Relative energies, and distances corresponding to nontrivial inter-ring interactions observed in the NOESY spectra are shown. Structure, where all corresponding distances are within 3 Å is shown in green frame. See preceding Figure S4 for drawing of NOESY correlations.

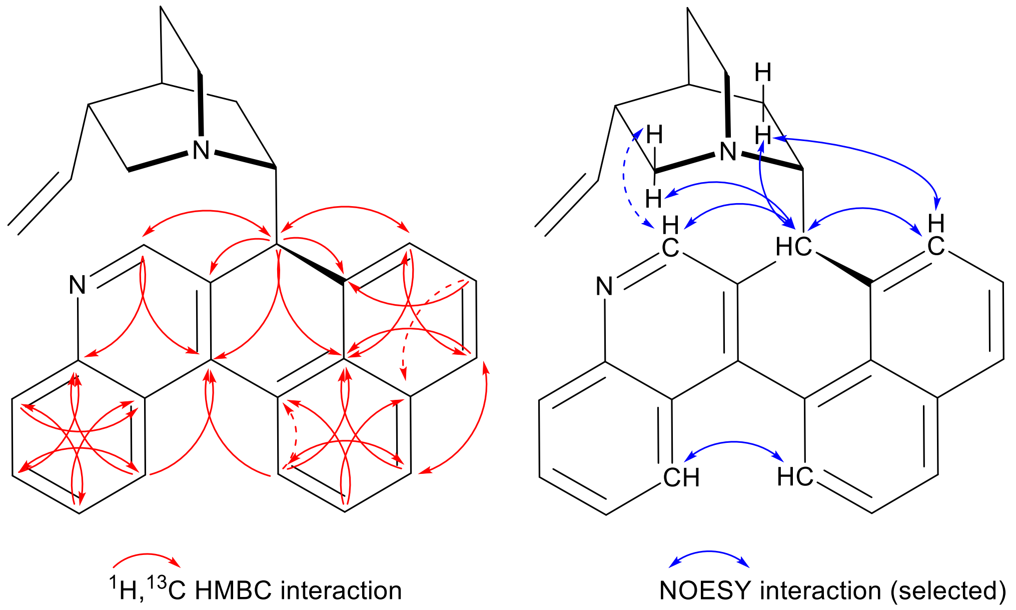

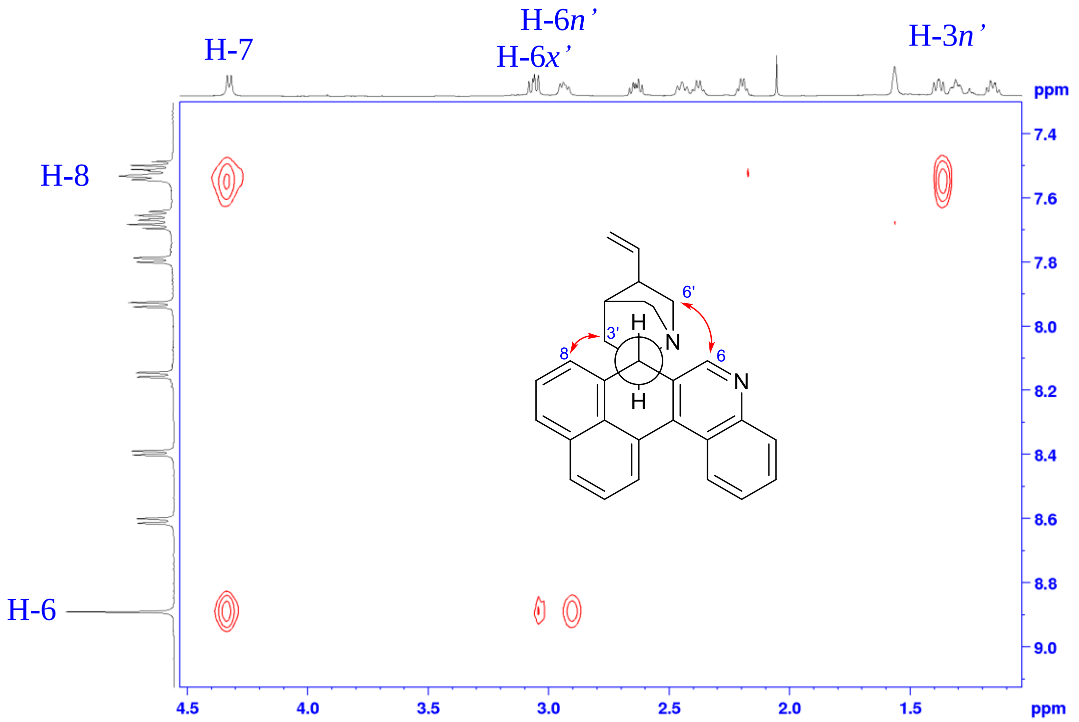

Figure S6. Selected HMBC (top left) and NOESY interactions (top right), and 1.0-4.5 vs. 7.3-9.1 ppm expansion of NOESY spectra for **3** in $CDCl_3$, and relevant Newman (bottom) IUPAC atom numbering is used, see Figure S1 for reference. For complete sets of NMR spectra, see Figures S36-S38.

Table S1. Comparison of experimental chemical shifts (ppm) for **1** in $CDCl_3$ and Boltzmann-averaged scaled isotropic shifts obtained from GIAO/mPW1PW01/6-311+G(2d,p) calculation and SMD solvent model for plausible epimers: *like* (7*S*)-**1** and *unlike* (7*R*)-**1**.

C-atom	δ experiment	δ DFT *like*-**1**	δ DFT *unlike*-**1**
**2'**	62.78	65.05	67.67
**3'**	27.69	28.28	28.22
**4'**	28.22	32.78	32.87
**5'**	40.53	45.62	45.39
**6'**	56.61	56.89	56.91
**7'**	41.34	41.08	41.37
**8'**	28.71	30.55	30.50
**9'**	142.36	146.74	146.92
**10'**	113.79	112.60	112.71
**1**	104.86	103.10	103.07
**2**	157.96	157.44	157.15
**3**	119.37	120.17	119.62
**4**	131.75	132.00	131.93
**6**	150.29	148.77	149.79
**7**	47.17	46.23	47.14
**8**	125.50	129.26	127.14
**9**	126.14	124.82	125.15
**10**	125.95	126.22	126.95
**11**	128.83	130.70	130.04
**12**	125.23	124.20	125.15
**13**	126.02	125.78	125.87
**4a**	144.41	144.38	143.97
**6a**	132.60	129.80	133.95
**7a**	133.38	136.94	136.07
**7a^1**	130.36	127.61	128.50
**10a**	128.61	132.82	133.14
**13a**	133.29	130.22	131.11
**13b**	134.52	137.21	134.45
**13c**	126.00	125.70	126.09
**OCH_3**	55.53	53.24	53.11
**R^2**		0.9985	0.9988
**DP4+**	(^{13}C-data)	0.08%	99.92%
**DP4+**	(all data)	72.45%	27.55%

H-atom	δ experiment	δ DFT *like*-**1**	δ DFT *unlike*-**1**
**2'**	2.320	2.655	2.722
**3*n*'**	0.768	1.028	1.316
**3*x*'**	1.108	1.389	1.793
**4'**	1.613	1.593	1.698
**5'**	2.061	2.212	2.171
**6*n*'**	2.202	2.080	1.946
**6*x*'**	2.948	3.060	2.793
**7*c*'**	3.411	3.525	3.489
**7*t*'**	2.858	2.940	2.801
**8*c*'**	1.634	1.823	1.844
**8*t*'**	1.498	1.654	1.638
**9'**	5.344	5.771	5.779
**10*c*'**	4.729	5.010	5.007
**10*t*'**	4.651	4.843	4.858
**1**	8.017	8.260	8.292
**3**	7.335	7.471	7.437
**4**	8.049	8.129	8.106
**6**	8.749	8.695	8.512
**7**	4.204	4.483	4.525
**8**	7.502	7.556	7.734
**9**	7.798	7.646	7.676
**10**	7.497	7.910	7.994
**11**	7.932	8.025	8.060
**12**	7.692	7.686	7.745
**13**	8.445	9.066	9.172
**OCH_3**	3.934	4.143	4.079
**R^2**		0.9960	0.9957
**DP4+**	(^{1}H-data)	99.97%	0.03%

For IUPAC atom numbering see Figure S1. Scaling factors used for converting computed isotropic shieldings to chemical shifts: slope: −1.0448, intercept: 186.0596.[S1,S2] DP4 probability calculation was performed using ready spreadsheet and indicating similar basis set.[S3,S4]

[S1] M. W.Lodewyk, M. R. Siebert, and D. J. Tantillo *Chem. Rev.* 2012, **112**, 1839.
[S2] http://cheshirenmr.info/Recommendations.htm (Accessed: 31 Oct 2021)
[S3] M. M. Zanardi and A. M. Sarotti, *J. Org. Chem.*, 2021, **86**, 8544–8548.
[S4] https://sarotti-nmr.weebly.com/custom-dp4.html (Accessed 31 Oct 2021)

Table S2. Comparison of experimental chemical shifts (ppm) for **1** in benzene-d_6 and Boltzmann-averaged scaled isotropic shifts obtained from GIAO/mPW1PW01/6-311+G(2d,p) calculation and SMD solvent model for plausible epimers: *like* (7*S*)-**1** and *unlike* (7*R*)-**1**.

C-atom	*δ* experiment	*δ* DFT *like*-**1**	*δ* DFT *unlike*-**1**
**2'**	62.97	65.11	67.65
**3'**	27.75	28.45	28.51
**4'**	28.25	32.78	32.90
**5'**	40.43	45.79	45.55
**6'**	56.51	57.13	57.12
**7'**	41.00	41.28	41.54
**8'**	28.51	30.83	30.82
**9'**	142.06	145.86	146.04
**10'**	113.42	113.01	113.12
**1**	104.86	102.49	102.41
**2**	158.11	157.35	157.03
**3**	119.25	119.96	119.39
**4**	132.47	132.48	132.44
**6**	150.71	148.40	149.66
**7**	47.01	46.43	47.42
**8**	125.87	129.43	126.74
**9**	125.31	124.70	124.84
**10**	125.98	126.13	126.86
**11**	128.50	130.31	129.45
**12**	125.14	123.85	124.97
**13**	125.92	125.22	125.34
**4a**	145.29	144.70	144.36
**6a**	132.75	129.20	133.35
**7a**	133.79	136.60	135.98
**$7a^1$**	130.63	127.69	128.59
**10a**	129.11	132.91	133.29
**13a**	133.57	130.42	131.50
**13b**	133.83	136.85	133.85
**13c**	126.09	125.69	126.06
**OCH_3**	54.58	53.11	52.98

R^2		0.9970	0.9979
**DP4+**	(^{13}C-data)	0.02%	99.98%
**DP4+**	(all data)	1.75%	98.25%

H-atom	*δ* experiment	*δ* DFT *like*-**1**	*δ* DFT *unlike*-**1**
**2'**	2.501	2.609	2.665
**3*n*'**	0.770	1.026	1.323
**3*x*'**	0.921	1.368	1.738
**4'**	1.363	1.585	1.685
**5'**	1.749	2.150	2.109
**6*n*'**	2.172	2.073	1.934
**6*x*'**	2.737	3.019	2.761
**7*c*'**	3.054	3.479	3.433
**7*t*'**	2.551	2.914	2.788
**8*c*'**	1.290	1.800	1.809
**8*t*'**	1.117	1.623	1.611
**9'**	5.017	5.683	5.692
**10*c*'**	4.569	4.969	4.967
**10*t*'**	4.414	4.808	4.823
**1**	8.021	8.141	8.163
**3**	7.249	7.393	7.359
**4**	8.367	8.107	8.087
**6**	9.010	8.638	8.474
**7**	3.932	4.379	4.411
**8**	7.199	7.503	7.623
**9**	7.337	7.560	7.580
**10**	7.602	7.812	7.896
**11**	7.619	7.912	7.940
**12**	7.306	7.580	7.647
**13**	8.312	8.989	9.108
**OCH_3**	3.379	4.078	4.060

R^2		0.9961	0.9940
**DP4+**	(^{1}H-data)	98.95%	1.05%

For IUPAC atom numbering see Figure S1. Scaling factors used for converting computed isotropic shieldings to chemical shifts: slope: −1.0448, intercept: 186.0596.[S1,S2] DP4+ probability calculation was performed using ready spreadsheet and indicating similar basis set.[S3,S4]

Table S3. Comparison of experimental ^{1}H and ^{13}C chemical shifts for **3** and PCM/GIAO/mPQ1PW01/6-311+G(d,p) computed isotropic shieldings for lowest energy structures of plausible epimers**:** *like* (7*R*)-**3** and *unlike* (7*S*)-**3** optimized at DFT/B3LYP/CC-pVDZ level.

C-atom	$\delta_{experiment}$	σ_{DFT} *like*-**3**	σ_{DFT} un*like*-**3**
**2'**	62.76	119.11	121.13
**3'**	26.12	158.43	158.53
**4'**	28.28	153.20	153.25
**5'**	39.86	139.89	140.14
**6'**	47.92	136.34	136.25
**7'**	49.36	135.74	135.72
**8'**	26.03	158.29	158.48
**9'**	140.26	36.88	37.19
**10'**	114.55	66.97	67.57
**1**	125.49	55.83	56.33
**2**	126.39	55.48	56.00
**3**	127.96	53.93	54.20
**4**	130.26	50.27	50.04
**6**	152.58	29.32	28.25
**7**	46.43	138.60	137.60
**8**	126.06	51.18	55.98
**9**	125.73	56.35	55.92
**10**	126.21	54.90	55.56
**11**	129.10	50.13	52.03
**12**	125.32	56.99	56.77
**13**	126.83	53.88	54.71
**4a**	148.32	31.75	32.52
**6a**	131.88	52.70	48.91
**7a**	132.95	44.95	46.80
**7a^1**	130.42	53.82	51.28
**10a**	128.31	48.70	49.11
**13a**	133.36	51.43	51.97
**13b**	135.90	42.65	44.99
**13c**	124.98	57.31	57.60
**R^2**		0.9973	0.9985
**DP4+**	(^{13}C-data)	0.00%	100.00%
**DP4+**	(all data)	0.00%	100.00%

H-atom	$\delta_{experiment}$	σ_{DFT} *like*-**3**	σ_{DFT} un*like*-**3**
**2'**	2.379	29.371	29.544
**3*n*'**	1.382	30.230	30.215
**3*x*'**	0.499	31.439	31.611
**4'**	1.565	30.446	30.438
**5'**	2.197	29.533	29.572
**6*n*'**	2.935	28.835	28.734
**6*x*'**	3.063	28.736	28.707
**7*c*'**	2.447	29.592	29.517
**7*t*'**	2.639	29.113	29.173
**8*c*'**	1.157	30.634	30.747
**8*t*'**	1.312	30.529	30.560
**9'**	5.874	25.037	25.098
**10*c*'**	5.044	26.313	26.324
**10*t*'**	5.086	26.398	26.399
**1**	8.606	22.460	22.702
**2**	7.526	23.708	23.803
**3**	7.653	23.629	23.694
**4**	8.150	23.170	23.215
**6**	8.889	22.527	22.558
**7**	4.327	27.342	27.391
**8**	7.537	23.913	23.854
**9**	7.497	23.812	23.783
**10**	7.793	23.536	23.490
**11**	7.932	23.427	23.364
**12**	7.681	23.777	23.623
**13**	8.394	22.509	22.914
**R^2**		0.9969	0.9977
**DP4+**	(^{1}H-data)	0.05%	99.95%

For IUPAC atom numbering see Figure S1. DP4+ probability calculation was performed using ready spreadsheet.[S3,S4]

S3. Attempts to identify radicals in the reaction mixture

Experiments with radical trapping generally disturb the formation of **Int.A**-type products. Instead of running the reaction in the presence of radical trap, the reaction was run as usual in a manner described in the experimental section for a defined incubation period. 15 minutes after adding a solution of 9*S*-chloroquinine to the Grignard from 1,8-dibromonaphthalene and stirring at 90 °C a sample (0.2 mL, up to 40 µmol for each reactant in THF/toluene) was withdrawn with a syringe and added under argon to solid 2,2,6,6-tetramethylpiperidinyloxyl (TEMPO, 5 mg, 32 µmol). After 1 minute of incubation, the solution was diluted with methanol then acetonitrile and analyzed with ESI-MS (Figure S20). For **Int.B•–Int.F•** adducts with TEMPO calculated formula is $C_{29}H_{48}N_3O_2$ and $[M+H]^+$ *m/z* 590.3741, found 590.3733.

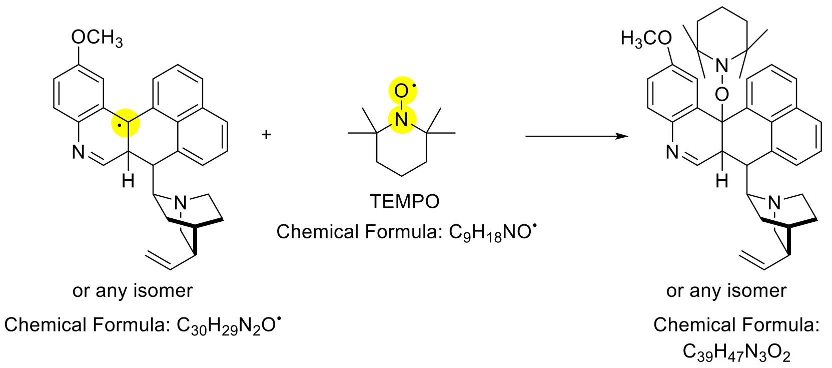

From the same initial reaction mixture solution stirred for 2.5 hours at 90 °C, another sample was withdrawn to a storage container. After additional 30 minutes the sample was placed in an EPR tube, partly exposed to air, and X-band EPR spectrum was recorded. Faint presence of paramagnetic species was observed (Figure S7). In another experiment, sample was withdrawn after 0.5 h reaction time, and placed in a quartz EPR capillary with limited air exposure, this time nearly no EPR signal was observed.

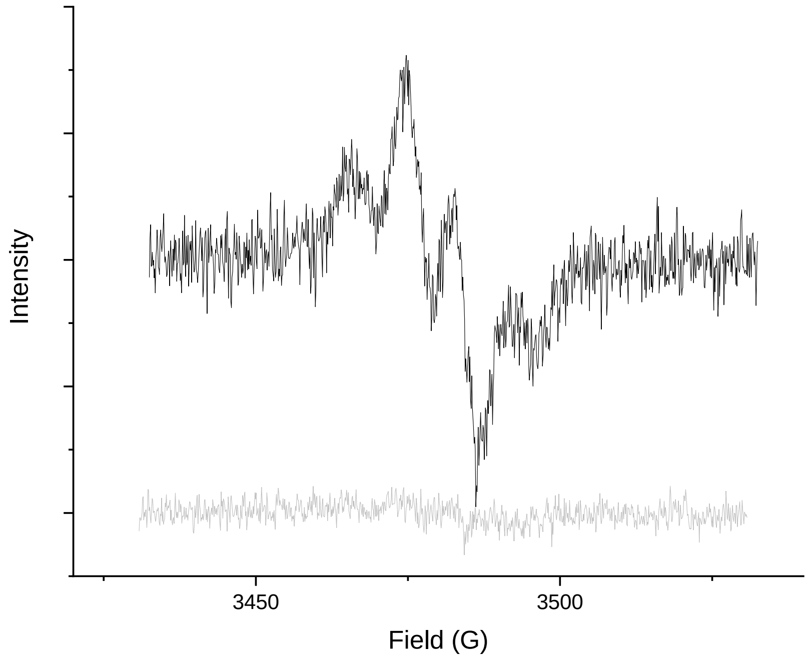

Figure S7. Room temperature X-band EPR spectra of a dissolved sample obtained from the reaction of 1,8-dibromonaphthalene with magnesium followed by addition of 9*S*-chloro-9-deoxyquinine and run for 150 min at 85-90 °C in THF / toluene (1:1). Spectrum was recorded after additional 0.5 h storage at room temperature in reaction solvent. The observed signal is centered on g-factor = 2.0030. Grey trace corresponds to a sample withdrawn after 30 min reaction time of another experiment. As indicated by the reviewer, the observed signals may originate from anti-oxidant impurities, and may not be conclusive.

In another experiment, sample of reaction mixture in THF/toluene was withdrawn and treated with neat DMPO. Then X-band EPR spectrum was recorded (Figure S8). The observed strong EPR trace was not interpreted as a single species.

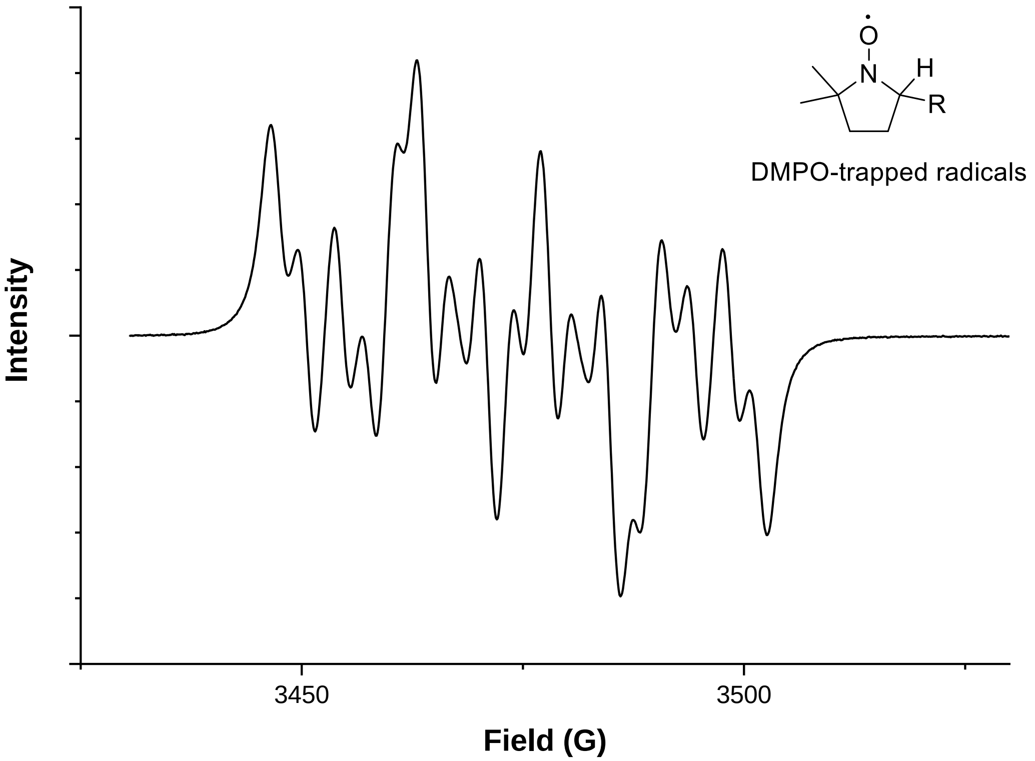

Figure S8. Room temperature X-band EPR spectra of a dissolved sample obtained from the reaction of DMPO (5,5-dimethyl-1-pyrroline-*N*-oxide) with a sample mixture taken from the reaction between 8-bromo-1-naphthylmagnesium bromide and 9*S*-chloro-9-deoxyquinine run for 30 min at 85-90 °C in THF / tolene (1:1).

S4. Assays of Grignard reagents

Identification of reactive magnesium species. In order to estimate the composition of Grignard reagent, i.e. 8-bromo-1-naphthylmagnesium bromide vs. 1,8-naphthalene-di(magnesium bromide (both including species related by Schlenk equilibrium), acid quenching experiment was performed. Expected reaction products included 1-bromonaphthalene and naphthalene, respectively, as well as unreacted 1,8-dibromonaphthalene. Retention times of expected products and relative responses measured from a mixture of three commercial compounds (Figure S9)

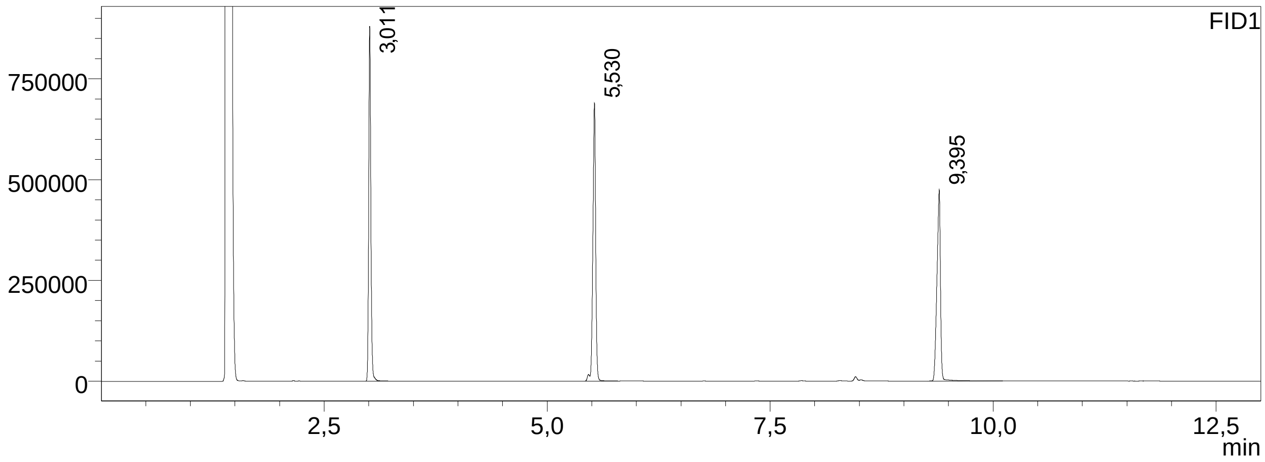

Peak#	Ret. Time	Area	Area %	Name
1	3,011	1456312	34,103	**Naphthalene, 49.1 mM**
2	5,530	1505586	35,257	**1-Bromonaphthalene, 52.5 mM**
3	9,395	1308470	30,641	**1,8-Dibromonaphthalene, 43.3 mM**

Figure S9. GC chromatography of naphthalene, bromonaphthalene and dibromonaphthalene reference solution in DCM, initial temperature 120°C, rate 10 °C/min.

Quenching was performed directly before addition of chloroquinine solution. A uniform sample of Grignard solution or suspension in THF (0.1-0.2 mL) was withdrawn and added to 1M aqueous $NaHSO_4$ solution (0.75 mL) and vigorously shaken for 1 min. Then ca. 0.75 mL DCM was added and shaken for 0.5 min. Part of DCM solution was withdrawn and diluted with fresh DCM before injecting to the GC instrument (Figure S10). Samples **A** and **B** correspond to experiments where magnesium was reacted in a sealed container for 18 h, while representative sample **C** was obtained under atmospheric pressure within 1 h.

Reaction **A**: 2.05 equiv Mg in THF for 18 h at 85 °C (Schlenk vessel)

Reaction **B:** 1.1 equiv Mg in THF for 18 h at 80 °C (Schlenk vessel)

Reaction **C:** 1.1 equiv Mg, 0.1 equiv $C_2H_4Br_2$ in THF for 1 h at reflux temperature (argon bubbler on top of a reflux condenser).

Sample of reaction mixture prepared similarly to reaction **B**, was diluted with benzene-d_6 and NMR spectrum was collected (Figure S41). Following exposure to air the sample displays NMR spectrum of 1-bromonaphthalene (Figure S42).

Experimental data for major organomagnesium species:

^{1}H NMR (600 MHz, THF/benzene-d_6) δ = 8.24 (d, J = 6.3 Hz, 1H), 7.66 (d, J = 7.4 Hz, 1H), 7.64 (d, J = 8.0 Hz, 1H), 7.49 (d, J = 8.0 Hz, 1H), 7.34 (dd, J = 6.3, 8.0 Hz, 1H), 7.01 (t, J = 7.7 Hz, 1H) ppm.

^{13}C NMR (151 MHz, THF/benzene-d_6) δ = 177.6, 141.0, 139.6, 135.7, 133.8, 129.9, 126.8, 126.0, 124.7, 124.2 ppm.

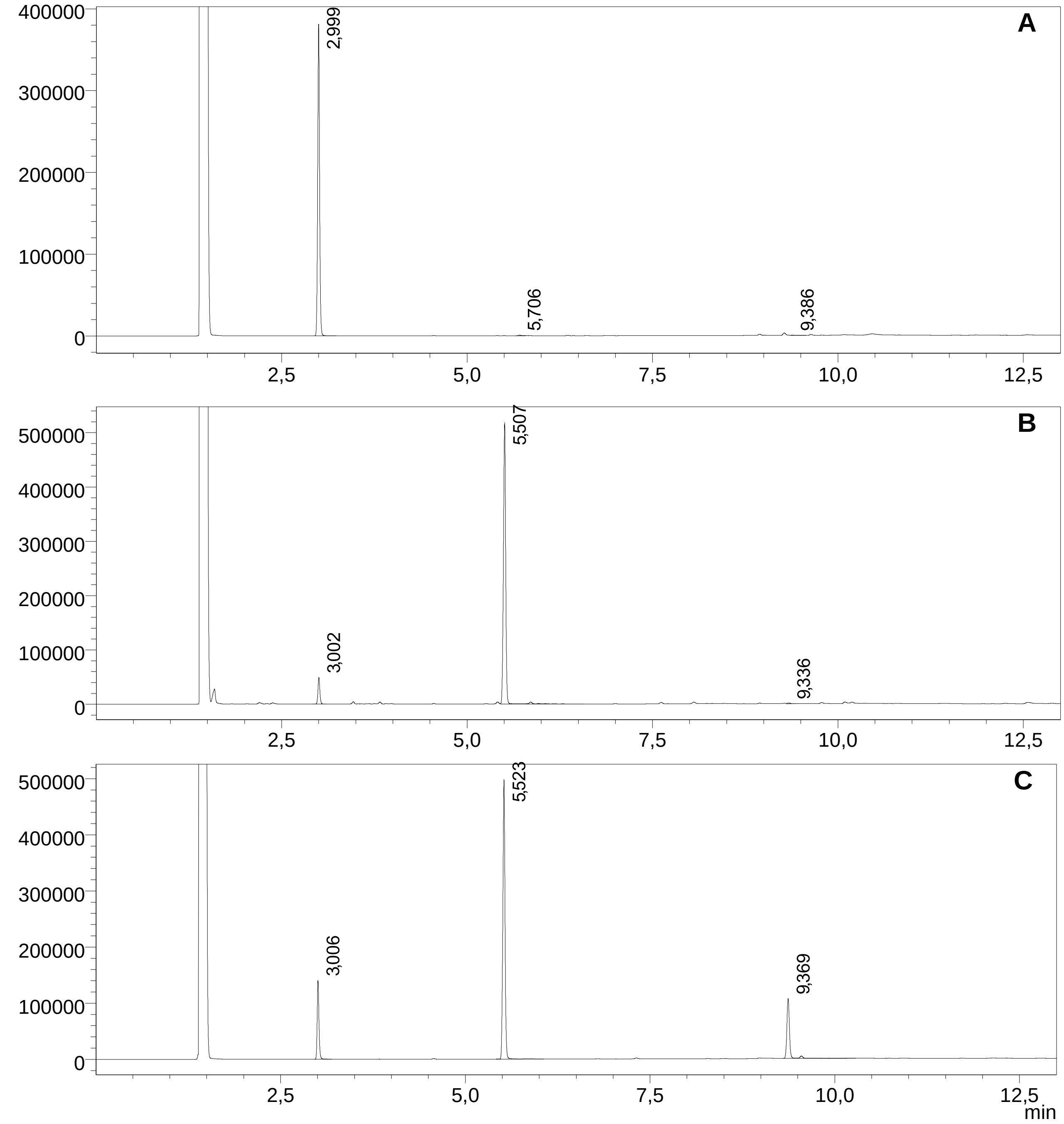

Peak#	Ret. Time, min	A		B		C		Compound
		Area	%	Area	%	Area	%	
1	2.99 - 3.01	667032	99.433	87017	7.262	233346	15.381	**Naphthalene**
2	5.51 - 5.70	2289	0.341	1106971	92.378	1018387	67.127	**1-Bromonaphthalene**
3	9.34 - 9.40	1512	0.225	4324	0.361	265378	17.492	**1,8-Dibromonaphthalene**

Figure S10. GC chromatography of two samples of Grignard reagents prepared from 1,8-dibromonaphthalene and quenched with 1M aqueous $NaHSO_4$. Reaction **A** (top): 2.05 equiv Mg in THF for 18 h at 85 °C (sealed Schlenk container); Reaction **B** (center) 1.1 equiv Mg in THF for 18 h at 80 °C (sealed Schlenk container); Reaction **C** (bottom) typical result for reaction with 1.1 equiv Mg (0.1 equiv CH_2Br_2) in THF for 1 h at reflux temperature (atmospheric pressure, inert gas flow). For chromatogram of reference compounds, see the preceding Figure S9.

S5. Peripheral discussion of alternative reaction mechanisms

Considered other pathways toward **1** included electrophilic / carbocation chemistry, carboanions, as well as charged and electrically neutral radicals. Lack of transition metals precludes such chemistry.[S5]

For the radical pathways the process which delivers the highest energy species **Int.B$^\bullet$** can be initiated by electron transfer from the Grignard reagent to the intermediate **Int.A**. However, the initial radical may also be considered in the first aryl-Grignard and 9-chloro-alkaloid coupling (Figure 11). Both processes will benefit from oligomeric nature of the Grignard reagents and coordination to the alkaloid scaffold.

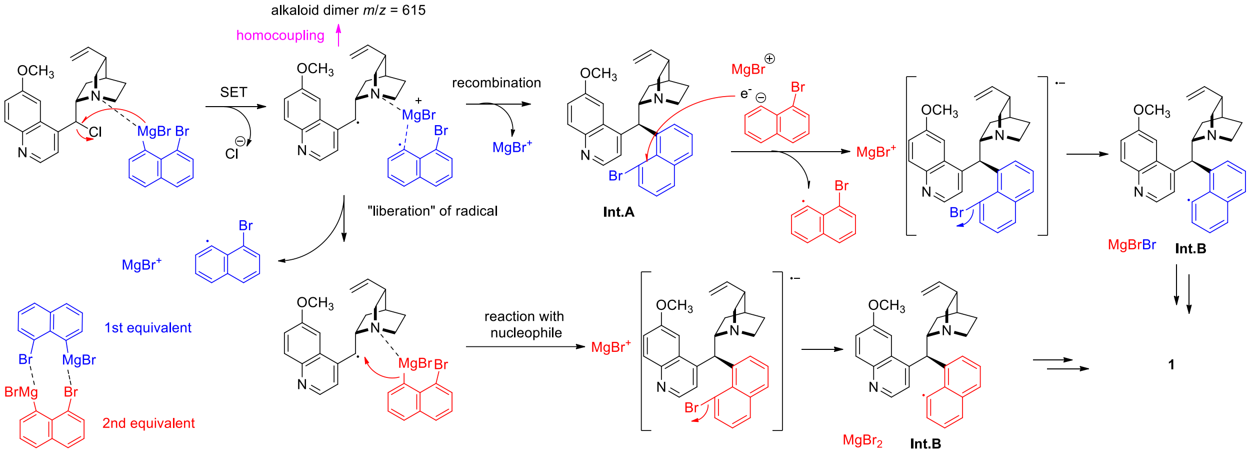

Figure S11. Outline of the plausible pathways SET pathways toward **Int.B$^\bullet$**

Plausible radical cation pathway, similar to the electrically neutral radical sequence was considered (Figure S12). Protonated (cationic) radicals can be obtained by protonation of radicals in the aqueous medium. Grignard reagents are incompatible with such conditions, and radicals like **Int.B$^\bullet$** can no longer be produced. However, **Int.D$^{\bullet+}$** can enter the process by aerobic one-electron oxidation of products such as **Int.I** belonging to the nucleophilic pathway. The major benefit of radical cation pathway is the differentiation of 9-epimeric radicals due to favorable intramolecular addition involving quinuclidine nitrogen atom. However, the same needed electron withdrawing effect can be achieved by coordination of magnesium ions. In the presence of any of these electrophiles neutral radical and radical cation pathways can interweave because **Int.C$^{+\bullet}$**, **Int.D$^{+\bullet}$**, **Int.E$^{+\bullet}$** correspond to the protonated forms of **Int.C$^\bullet$**, **Int.D$^\bullet$**, **Int.E$^\bullet$**, respectively.

[S5] (a) Shankar, M.; Rit, R. K.; Sau, S.; Mukherjee, K.; Gandon, V.; Sahoo, A. K. *Chem. Sci.* **2020**, *11*, 10770–10777. (b) Moon, S.; Nishii, Y.; Miura, M. *Org. Lett.* **2019**, *21*, 233–236. (c) Zhang, X.; Si, W.; Bao, M.; Asao, N.; Yamamoto, Y.; Jin, T. *Org. Lett.* **2014**, *16*, 4830–4833. (d) Dooley, J. D.; Reddy Chidipudi, S.; Lam, H. W. *J. Am. Chem. Soc.* **2013**, *135*, 10829–10836. (e) Thirunavukkarasu, V. S.; Donati, M.; Ackermann, L. *Org. Lett.* **2012**, *14*, 3416–3419.

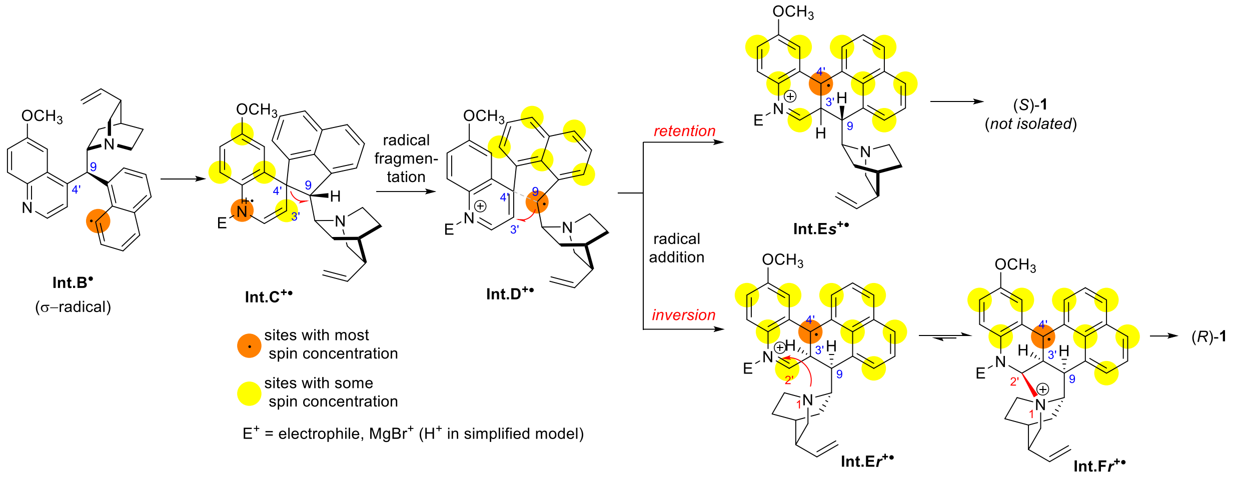

Figure S12. Outline of considered intermediates in the substitution-rearrangement reaction involving formal radical cations, and similarity with coordination of magnesium species.

For non-coordinated neutral radical pathway investigation of the proposed intermediates *i.e.*, isomeric unpaired electron species **Int.B•–Int.E•** was carried out by calculation at the DFT/B3LYP/CC-pVDZ and M06-2X/CC-pVDZ levels of theory (Figure S13). Intermediate **Int.B•** lacking one carbon-carbon bond and with localized unpaired spin is the highest energy particle (+28 kcal/mol), while **Int.C•** has 5-8 kcal/mol energy excess compared to **Int.E•**. Thus, energy substantially decreases along the reaction pathway. The relative energy of relaxed **Int.D•** is greatly dependent on the calculation method (from +5.0 to −1.7 kcal/mol vs. **Int.E•**). Unlike for the radical cation, there is no apparent difference in energies consistent with the observed stereoselectivity.

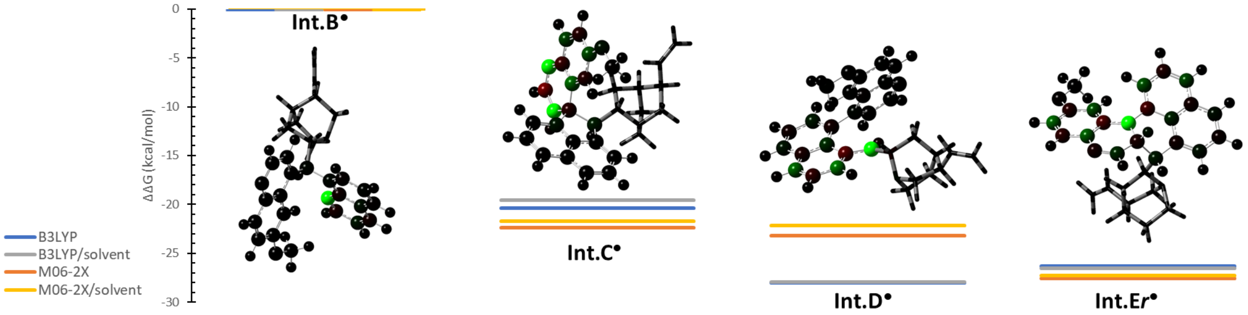

Figure S13. Structures and computed ground state free energies at the DFT/B3LYP or M06-2X levels of theory (gas phase and SMD solvent model for THF; energies relative to **Int.B•**) with CC-pVDZ basis set of intermediate radicals **Int.B•**, **Int.C•**, **Int.D•** and **Int.*Er*•**. Calculated spin density represented by green color intensity.

It can be speculated that poorer reaction outcomes with 5,6-dibromoacenaphthene may be partly caused by radical quenching through H-abstraction from position 1 of acenaphthene. This may be supported by unusually high abundance of 6-methoxycinchonan (*m*/*z* = 309, monocharged, *cf.* Figure S19).

Carbocation process, is consistent with the reactivity of quinoline at position 3 for electrophilic aromatic displacement. However, the carbocation is unlikely to form under acid free conditions even during workup and the product of such reaction is expected to be significantly epimerized at the migrating stereogenic center.

Carboanion process can explain nucleophilic addition at position 4 of the quinoline, analogously to the report by Hintermann (Ref. 4, main text) and lead to intermediate **Int.H**. (Figure S14). This sequence

would be the expected for bis-Grignard reagents (naphthalen-1,8-diyldimagnesium[S6] compounds). After workup of reactions run with pure bis-Grignard ESI-MS identified base peak at *m/z* 435, which could be attributed to hydrolyzed **Int.G** and/or **Int.I**. There are no reports of reactions that are consistent with nucleophilic migration[S7] that could originate from the formal anionic intermediates such as **Int.H**. However spirocyclic acenaphthene derivatives similar to **Int.I** are prone to oxidation and bond homolysis and could converge to the radical cation or neutral radical pathways.[S8] This is unlikely to constitute a major reaction pathway.

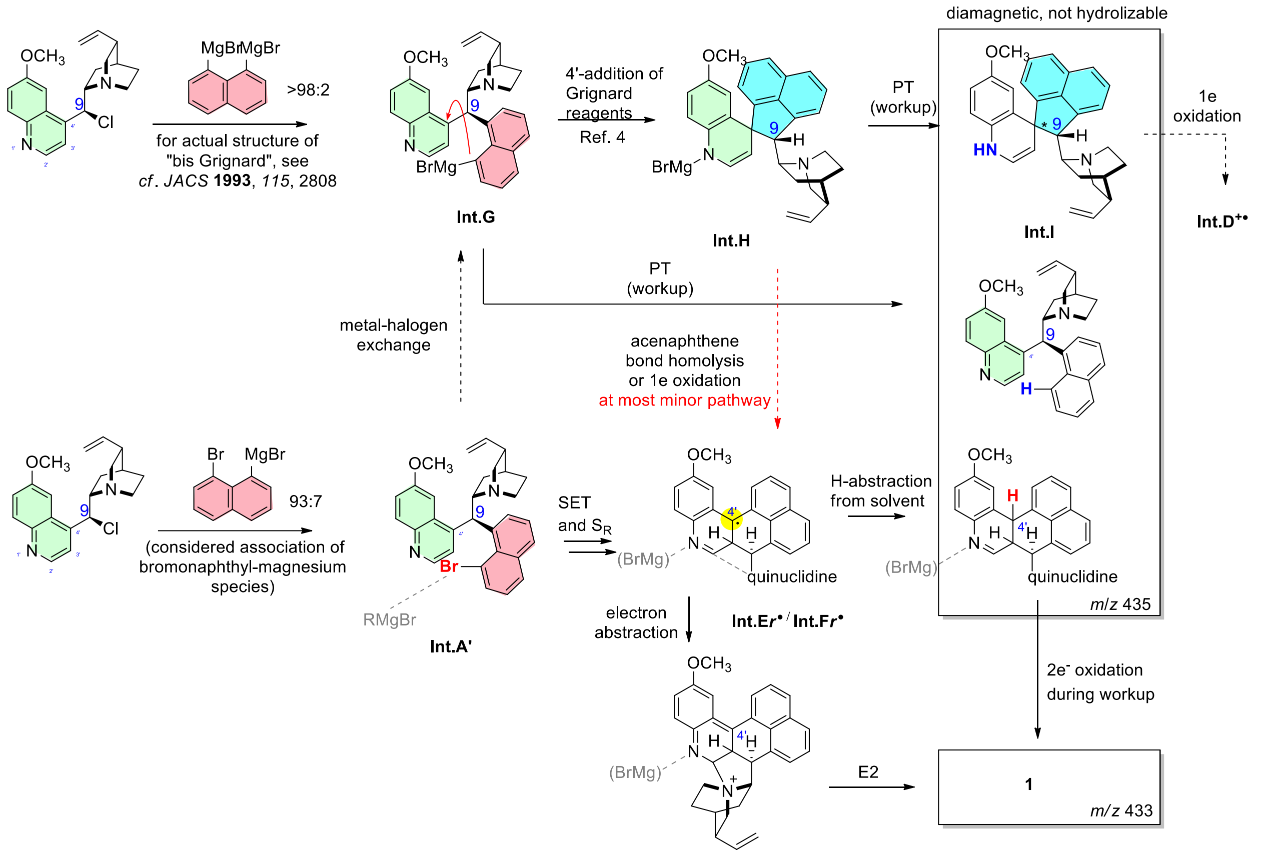

Figure S14. Outline of plausible carbanionic process, and its relation to other considered mechanisms.

For the other observed byproducts (Figure S15, and for ESI-MS *cf.* Figures S16-S18) following pathways are proposed:

[S6] Tinga, M. A. G. M.; Schat, G.; Akkerman, O. S.; Bickelhaupt, F.; Horn, E.; Kooijman, H.; Smeets, W. J. J.; Spek, A. L. *J. Am. Chem. Soc.* **1993**, *115*, 2808–2817.
[S7] T. S. Stevens, *Electrophilic Molecular Rearrangements. In Progress in Organic Chemistry*: Volume 7; Cook, J., Carruthers, W., Eds.; Springer US: Boston, MA, 1968; pp 48–74.
[S8] (a) Kawai, H.; Takeda, T.; Fujiwara, K.; Wakeshima, M.; Hinatsu, Y.; Suzuki, T. *Chem. Eur. J.* **2008**, *14*, 5780–5793. (b) Kawai, H.; Takeda, T.; Fujiwara, K.; Suzuki, T. *Tetrahedron Lett.* **2004**, *45*, 8289–8293

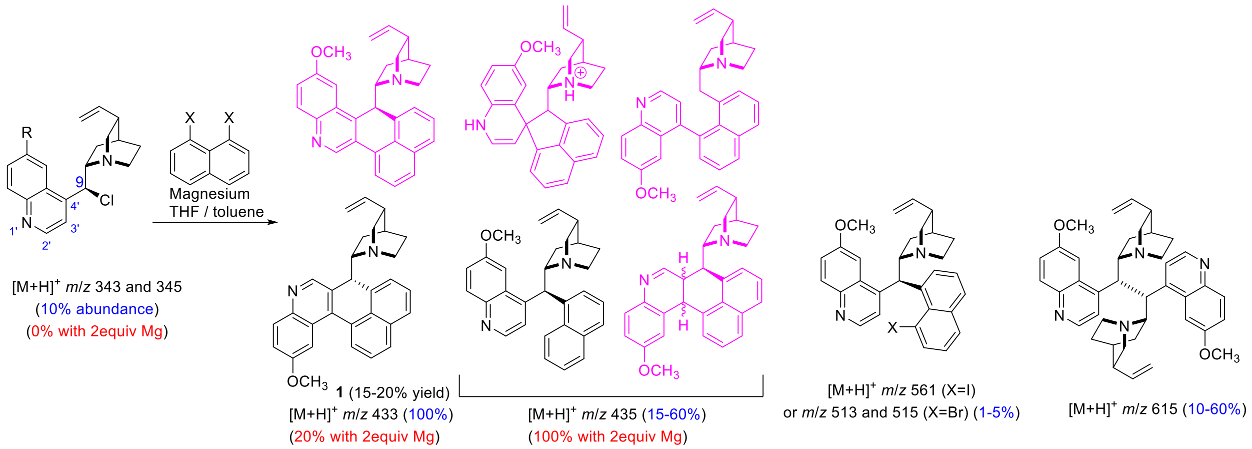

Figure S15. Outline of identified (black), and plausible (magenta) components observed in the crude MS spectra and their approximate relative abundance (*cf*. Section S6)

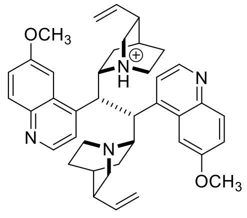

Exact Mass: 615.369

Dimer (for quinine *m/z* = 615), the mechanism for dimer formation as a radical recombination process occurring in the reaction of 9-chloroalkaloids with Grignard or organolithium reagents was described in earlier work[S9]

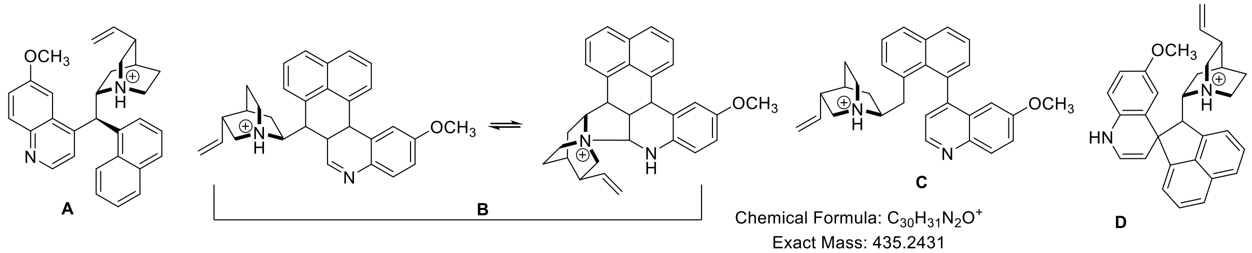

Products incorporating naphthyl residue (for quinine *m/z* = 435) with 2 extra hydrogen atoms compared to the rearranged product **1**: known 9*S*-naphth-1-yl-quinine (Structure **A**) was observed in the spectra of some chromatographic fractions. Its formation is likely a result of single bromine to metal exchange followed by the reaction with a proton source. This may precede or follow the Wurtz-type coupling. Alternatively, the product could be produced by hydrogen abstraction from solvent to any of the proposed isomeric radical intermediates **Int.B•**−**Int.F•** or similar radical.

Substantial contribution of this mass is associated with the reaction of bis-Grignard and subsequent hydrolysis of organomagnesium compound **Int.G** (*cf*. Figure S14; structure **A**) and possibly cyclized product **Int.I** (structure **D**).

[S9] P. J.Boratyński, I. Turowska-Tyrk, and J. Skarżewski *J. Org. Chem.* 2008, **73**, 7357.

S6. Plots of ESI-MS spectra

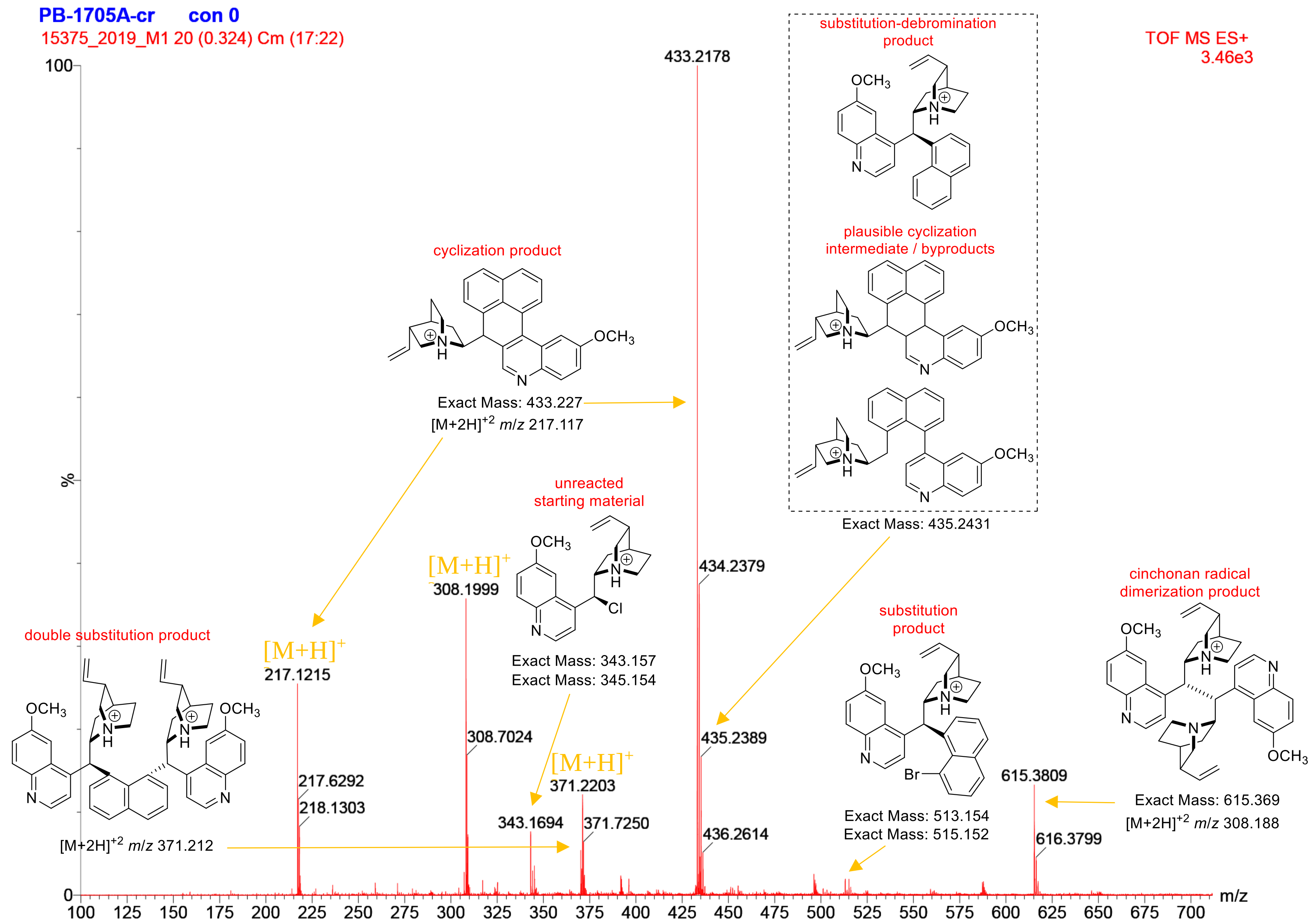

Figure S16. ESI-MS (methanol) of a reaction mixture obtained from the completed reaction of 1,8-dibromonaphthalene with magnesium and 9*S*-chloroquinine. Plausible structures corresponding to some of the observed signals were drawn. For the reaction mixture obtained from the reaction mixture before completion (3 h) see the following Figure S16. For spectrum of purified material, see Figure S18.

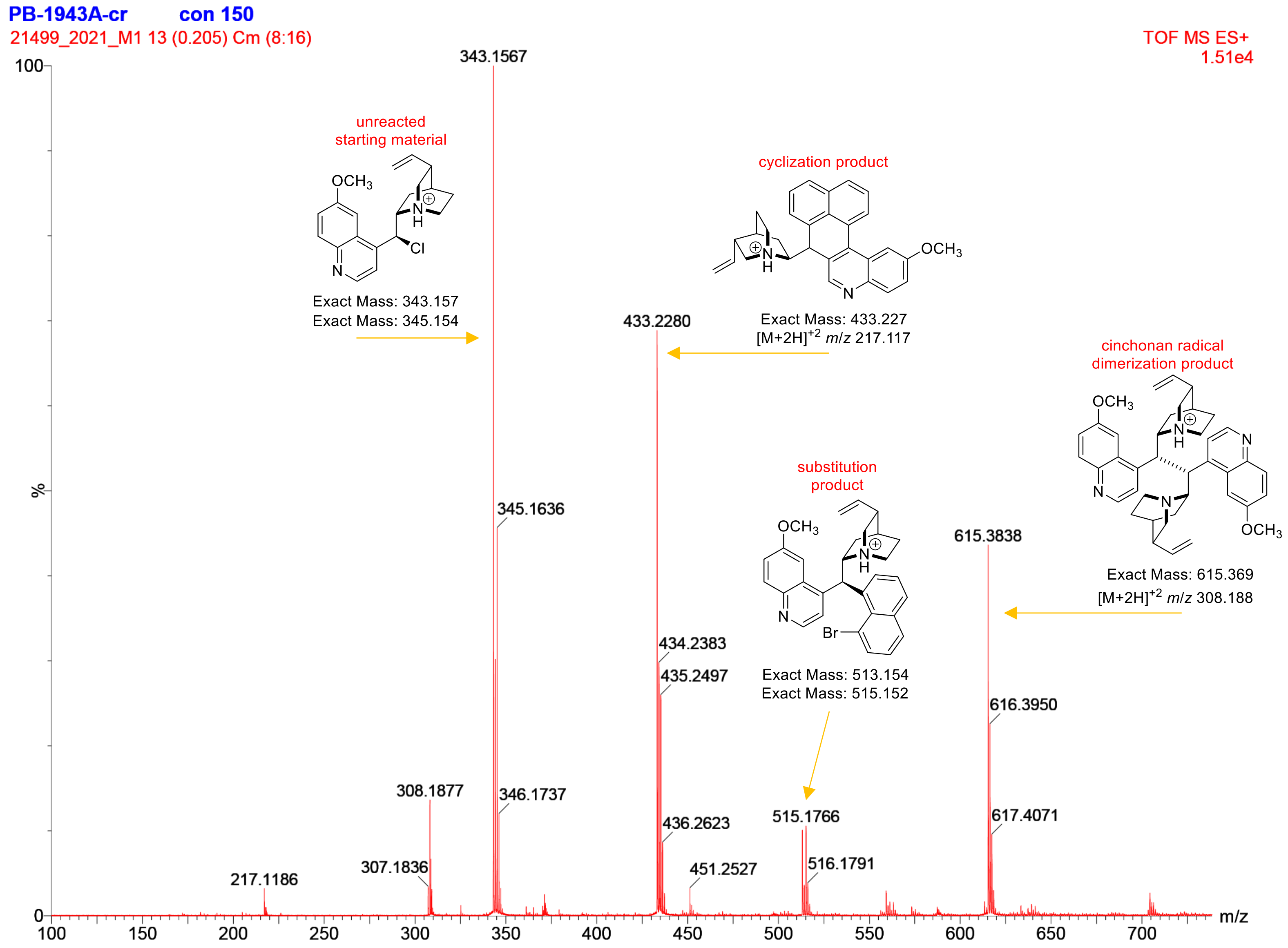

Figure S17. ESI-MS (methanol) of a reaction mixture obtained from the reaction of 1,8-dibromonaphthalene with magnesium and 9*S*-chloroquinine run for only 3 h at 85 °C. For a similar spectrum obtained from reaction run for 18 h at 85-90 °C, see the preceding Figure S16.

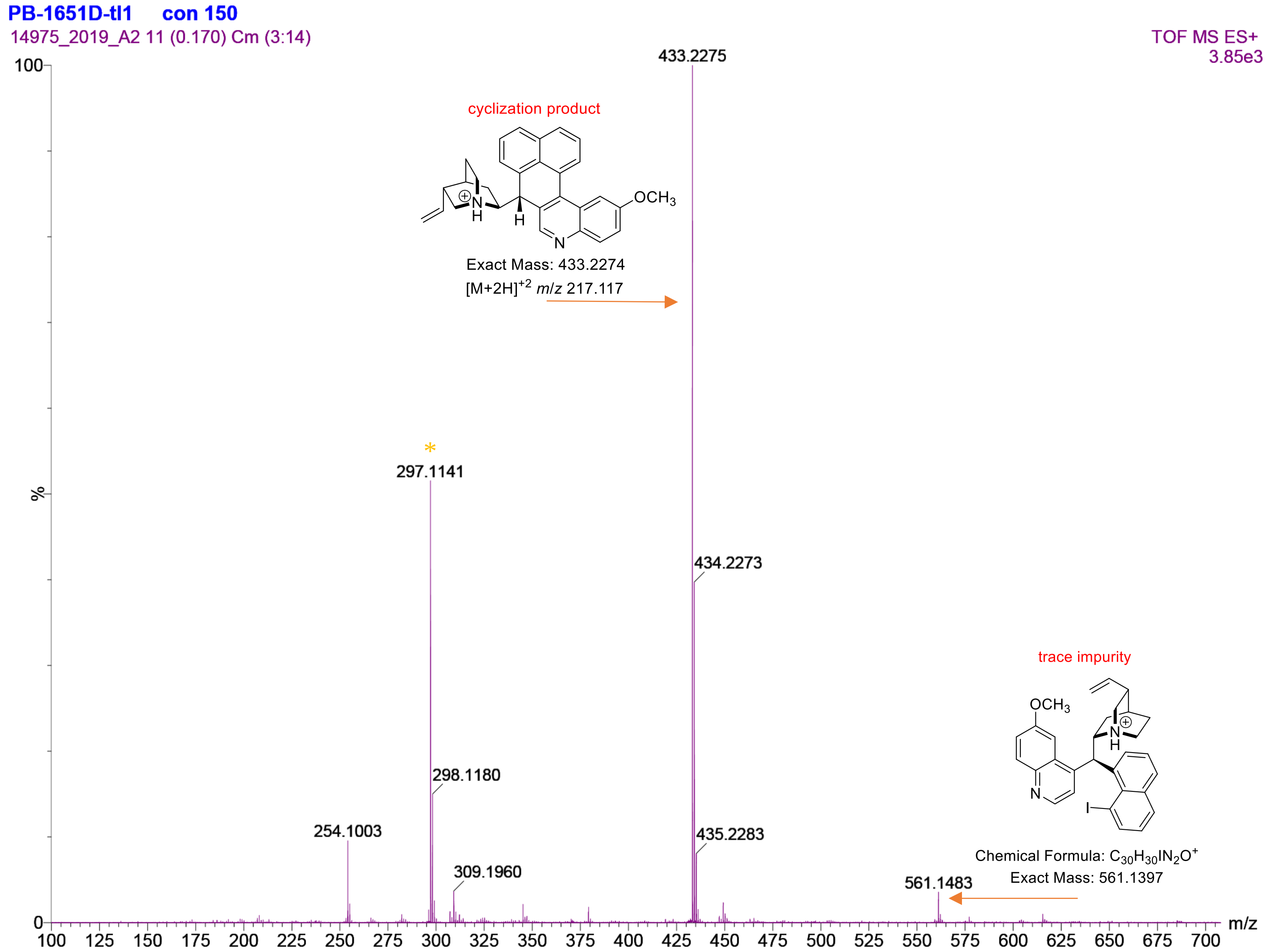

Figure S18. Example of ESI-HRMS (acetonitrile) of a purified reaction product **1**. *fragmentation ion.

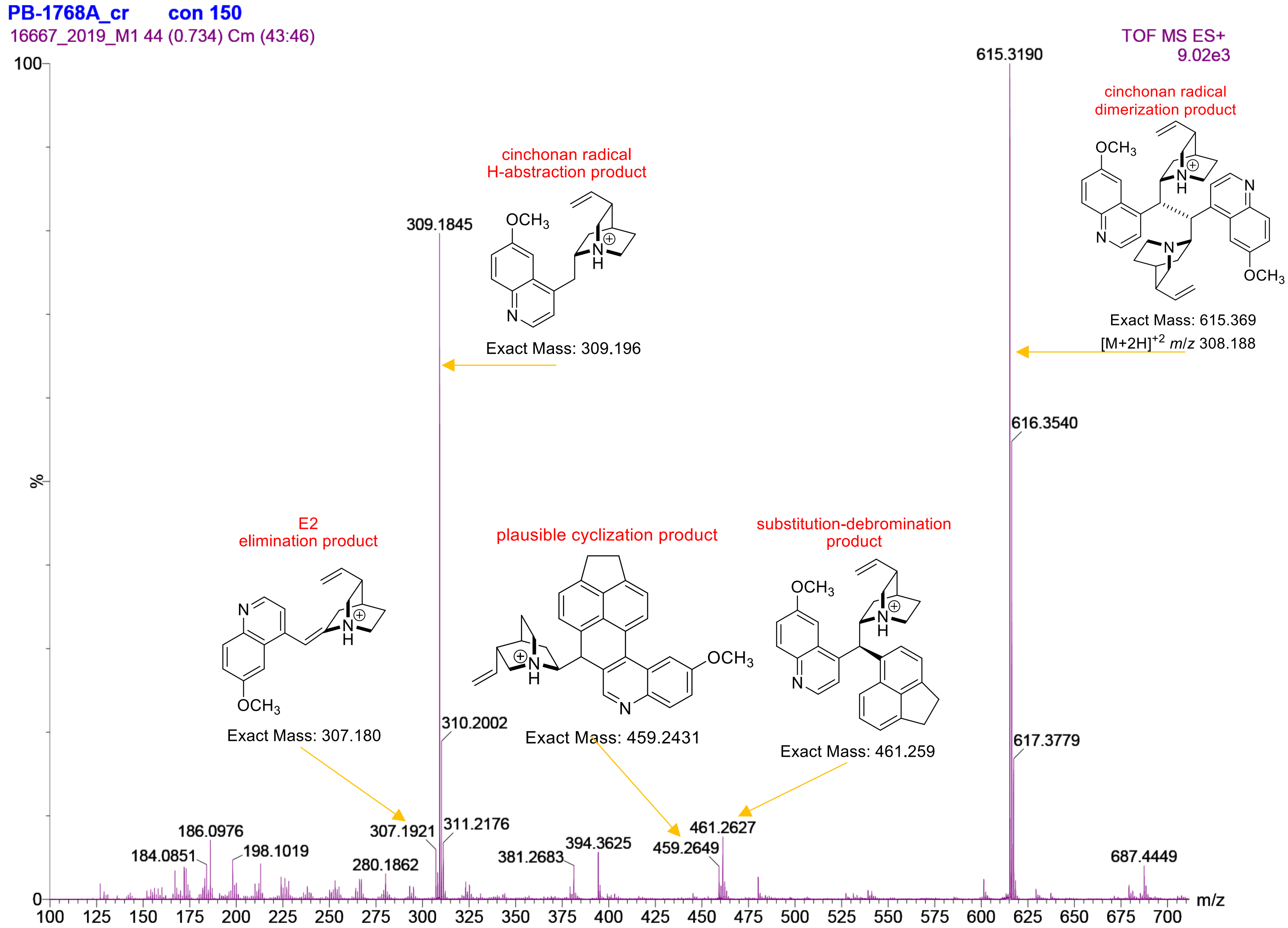

Figure S19. ESI-MS (methanol) of a reaction mixture obtained from the reaction of 5,6-dibromoacenaphthene with magnesium and 9*S*-chloroquinine. Plausible structures corresponding to some of the observed signals were drawn.

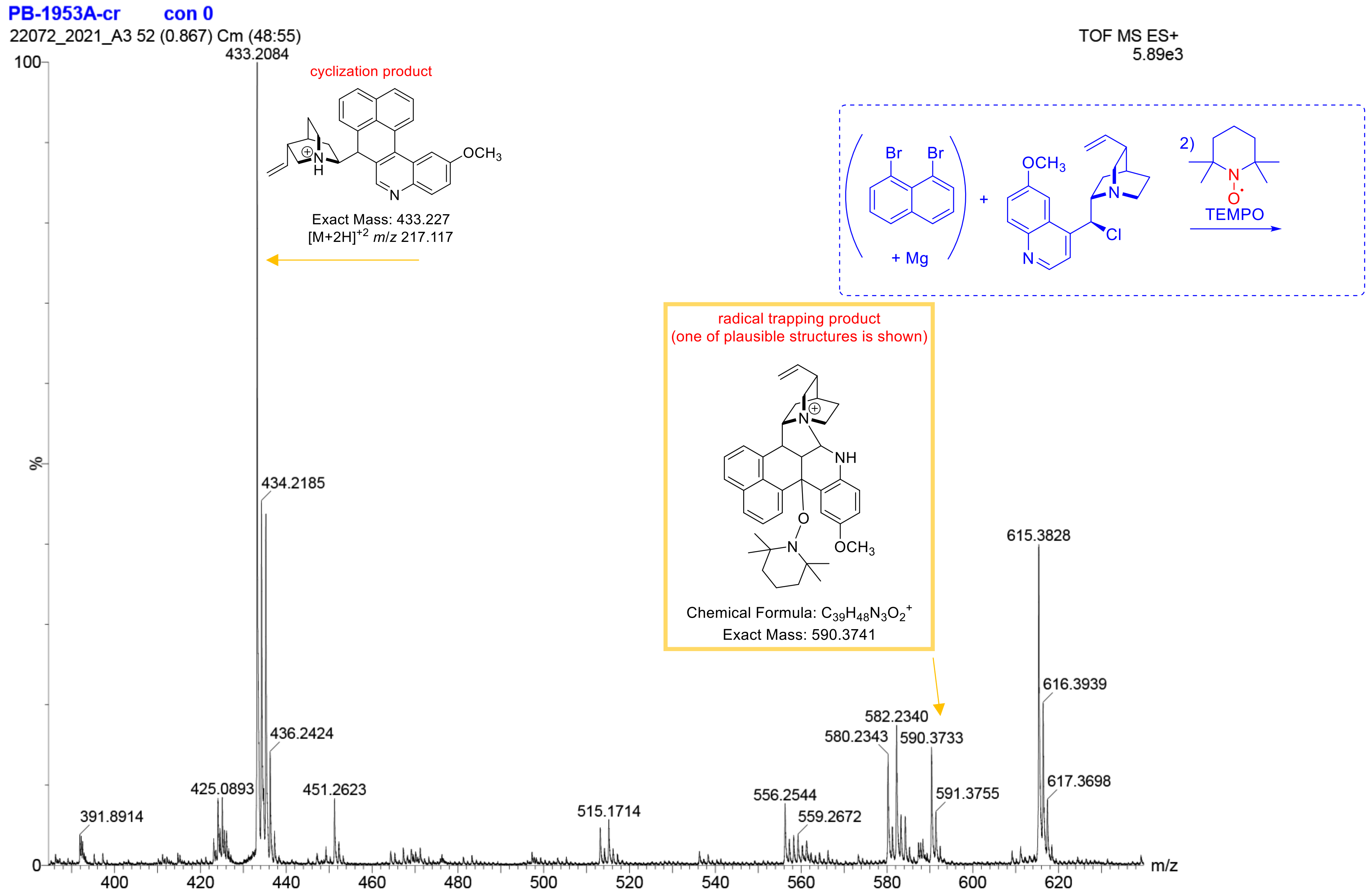

Figure S20. ESI-MS (acetonitrile) of a reaction mixture obtained from the reaction of 1,8-dibromonaphthalene with magnesium and 9*S*-deoxy-9-chloroquinine run for 15 min at 85-90 °C followed by quenching of a 0.2 mL sample with excess of solid TEMPO (2,2,6,6-tetramethylpiperidinyloxyl, 5 mg). Recombination of radicals **Int.B•** − **IntF•** with TEMPO is expected to produce *m*/*z* 590.3741 (Δ*m*/*z* 1.4 ppm). The most abundant signal of the starting material (*m*/*z* 343 and 345) is not shown.

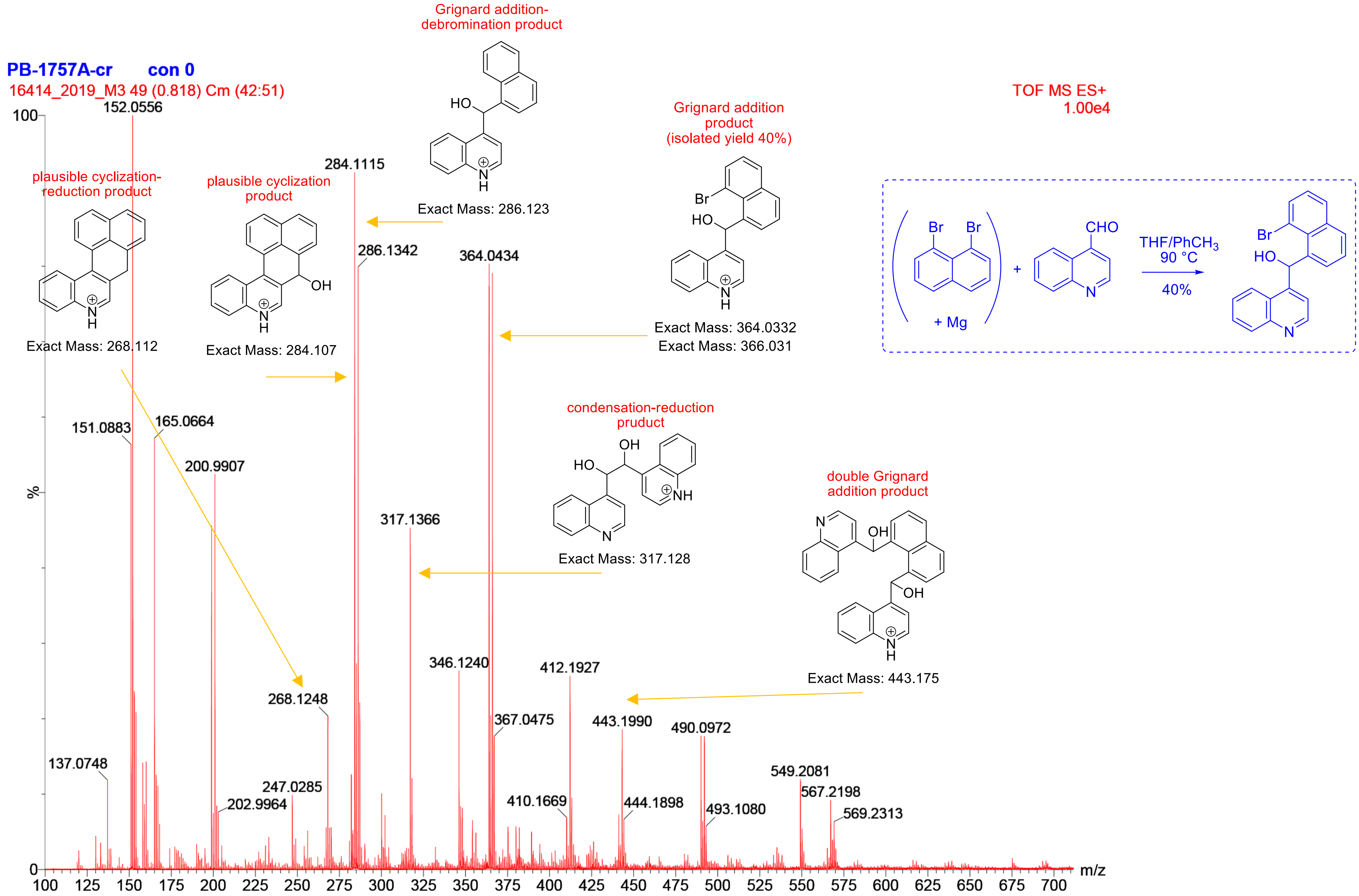

Figure S21. ESI-MS (methanol) of a crude mixture obtained from the reaction of 1,8-dibromonaphthalene with magnesium and 4-quinoline carbaldehyde (frame). Plausible structures corresponding to some of the observed signals were drawn.

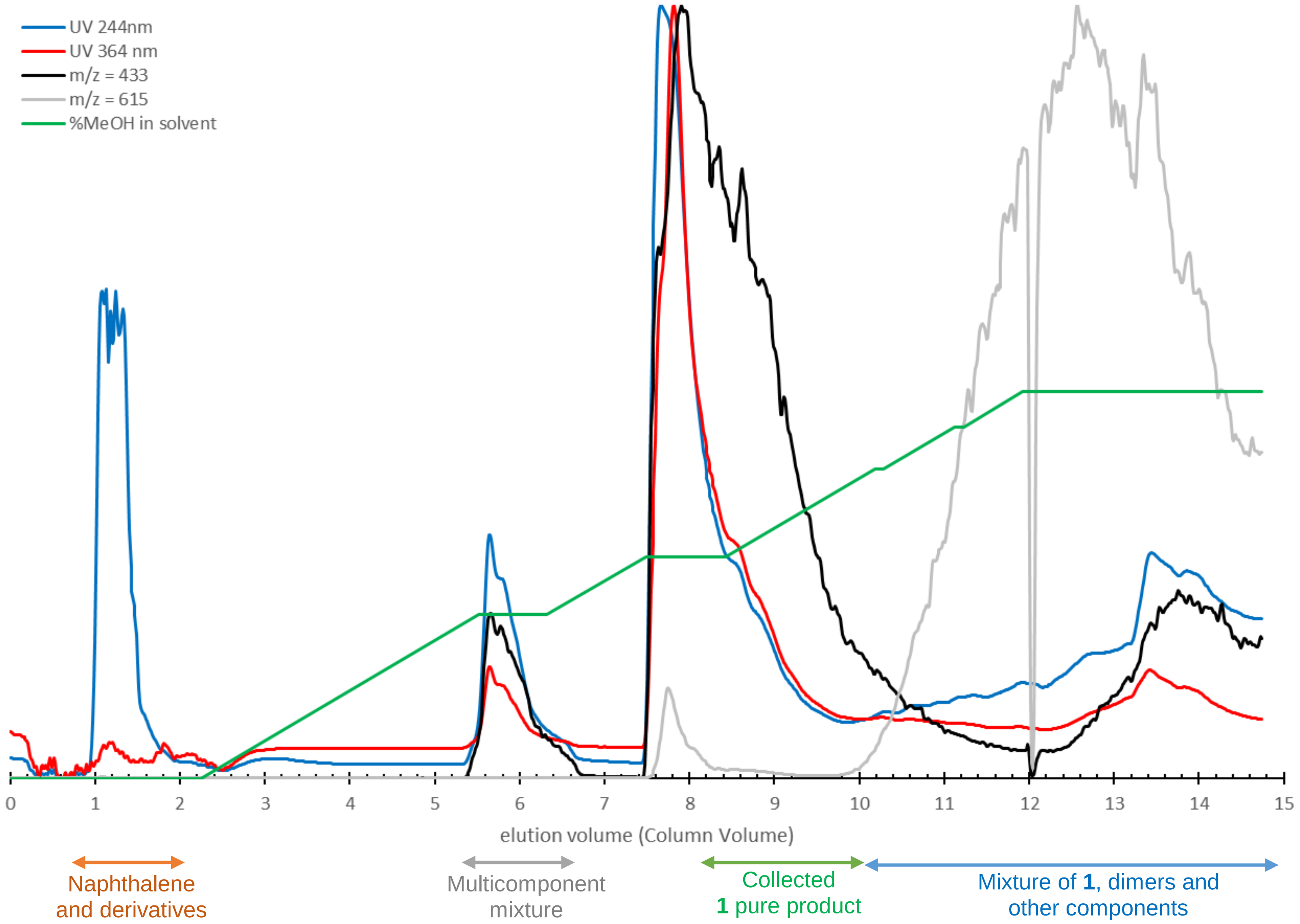

Figure S22. Example of silica gel flash chromatography profile for separation of **1** from crude mixture: monitored by UV absorbance at 244 (blue line) and 364 nm (red line), and mass detection at *m/z* = 433 (expected product, black line) and *m/z* = 615 (quinine dimer, grey line). Each plotted signal is normalized. Eluent composition: dichloromethane/methanol (0% to 10%, green line)

S7. UV-Vis absorption and CD spectroscopy

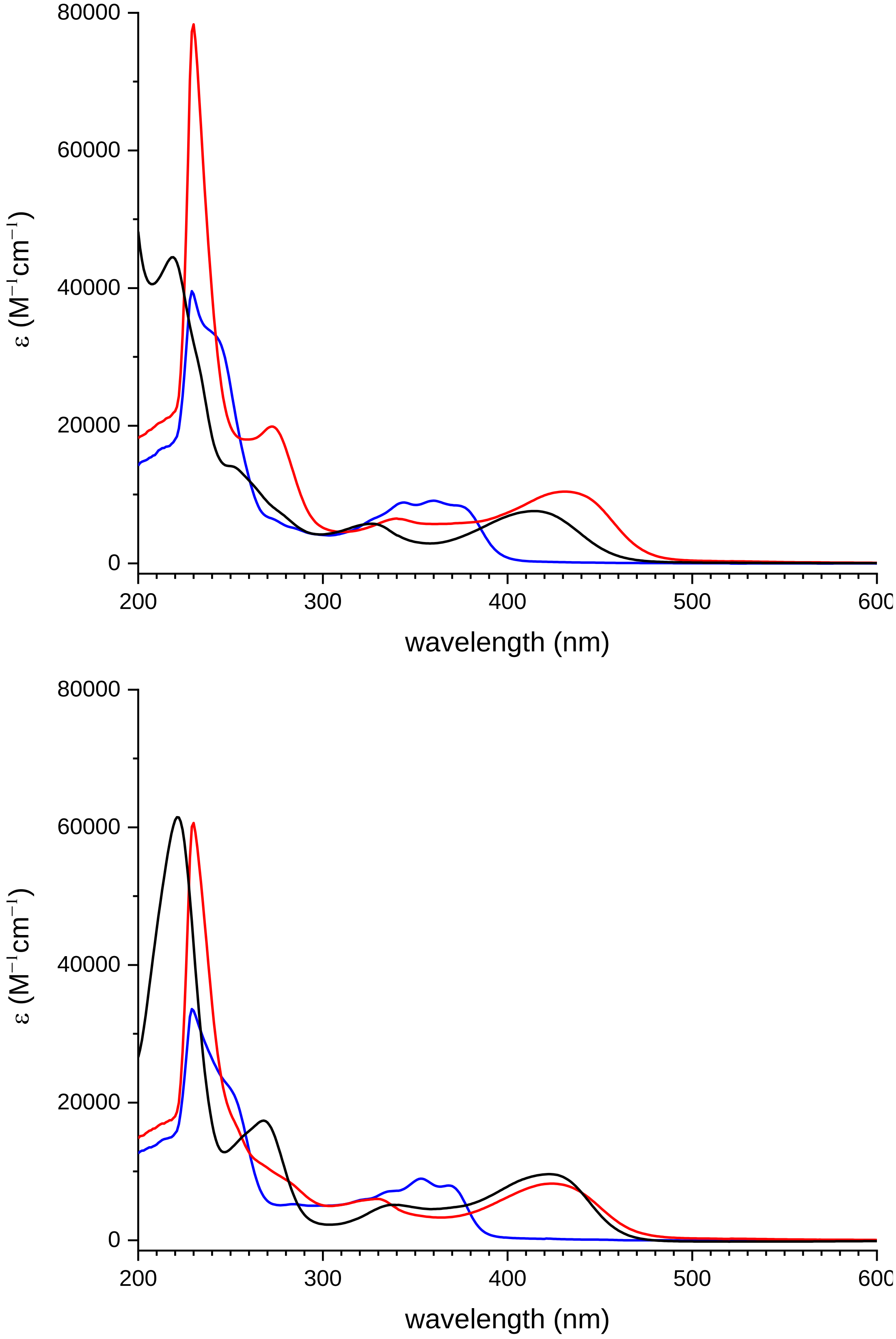

Figure S23. UV-Vis spectra for **1** (top) and **3** (bottom) in DCM in the absence of acid (blue lines), in 15×10^{-3} M TFA / DCM (red lines), and in aqueous 0.5 M H_2SO_4

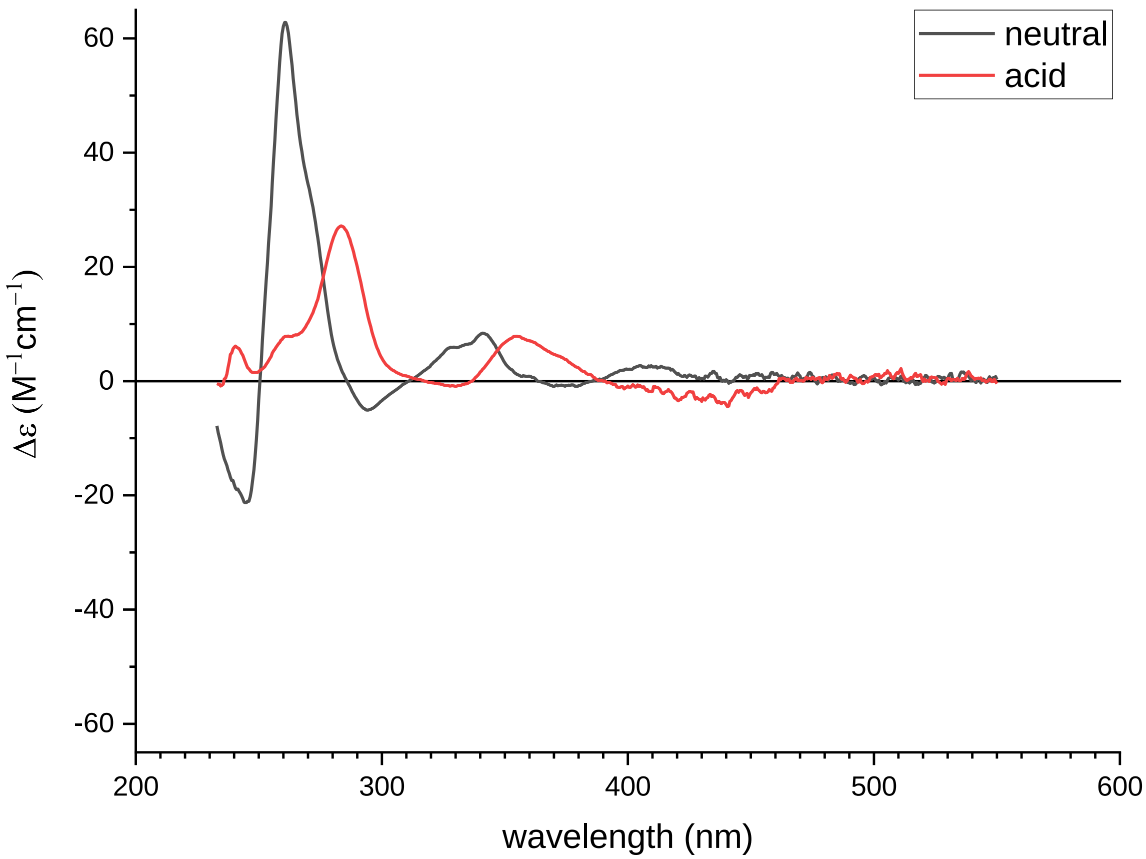

Figure S24. Circular dichroism (CD) spectra of 10^{-4} M solution of **1** in DCM in the absence of acid (black lines) and in $15{\times}10^{-3}$ M TFA (red lines)

S8. Fluorescence spectroscopy

Fluorescence measurements for solutions in DCM, DCM with 0.015 M TFA, and 0.5 M aqueous H_2SO_4 revealed significant differences only in spectra for neutral/basic solutions of **1** and **3**. In acidic solutions the spectra essentially converge with similar both maximum excitation (λ_{EX} = 413 − 428 nm) and emission wavelength (λ_{EM} = 522 − 524 nm) for **1** in DCM/TFA, **1** in H_2O/H_2SO_4, and **3** in sulfuric acid. Slightly lower maximum emission wavelength was observed for **3** in DCM/TFA (Figure S25).

Estimated fluorescence yield for the cinchonine-derived **3** remains essentially constant $\Phi_F = 0.53 \pm 0.06$ regardless of the nature of the solution. For quinine derivative **1**, with the methoxy substituent in the quinoline ring, there is noticeable variation in fluorescence yield Φ_F = 0.32 – 0.45 in different solutions. In H_2O/H_2SO_4 the values of Φ_F for **1** and **3** are almost identical (Table S4).

Table S4. Fluorescent properties of 10^{-5} M solutions of **1** and **3**

Solution \ Compound	**1**	**3**
Maximum excitation wavelength (λ_{EX}, nm)		
DCM	380	372
DCM, 0.015 M TFA	428[a]	426
0.5 M H_2SO_4 in H_2O	420	413
Maximum emission wavelength (λ_{EM}, nm)		
DCM	436	410
DCM, 0.015 M TFA	522[a]	510
0.5 M H_2SO_4 in H_2O	524	522
Stokes shift ($\lambda_{EM}-\lambda_{EX}$, nm)		
DCM	56	38
DCM, 0.015 M TFA	94[a]	84
0.5 M H_2SO_4 in H_2O	104	109
Estimated fluorescence quantum yield (Φ_F)[b]		
DCM	0.419	0.541
DCM, 0.015 M TFA	0.321	0.582
0.5 M H_2SO_4 in H_2O	0.452	0.475

[a]For 10^{-4} M; [b]Fluorsecence quantum yield was estimated from single point measurement using quinine sulfate ca. 10^{-5} M in 0.5 M aqueous H_2SO_4 (excitation wavelength 350 (5) nm) as reference assuming quinine $\Phi_{F\,Ref.}$ = 0.54, n_D(0.5M H_2SO_4) = 1.346, n_D(DCM) = 1.424. No compensation for different excitation wavelengths corresponding to excitation maxima for measured samples may result in inaccurate estimation of quantum yield.

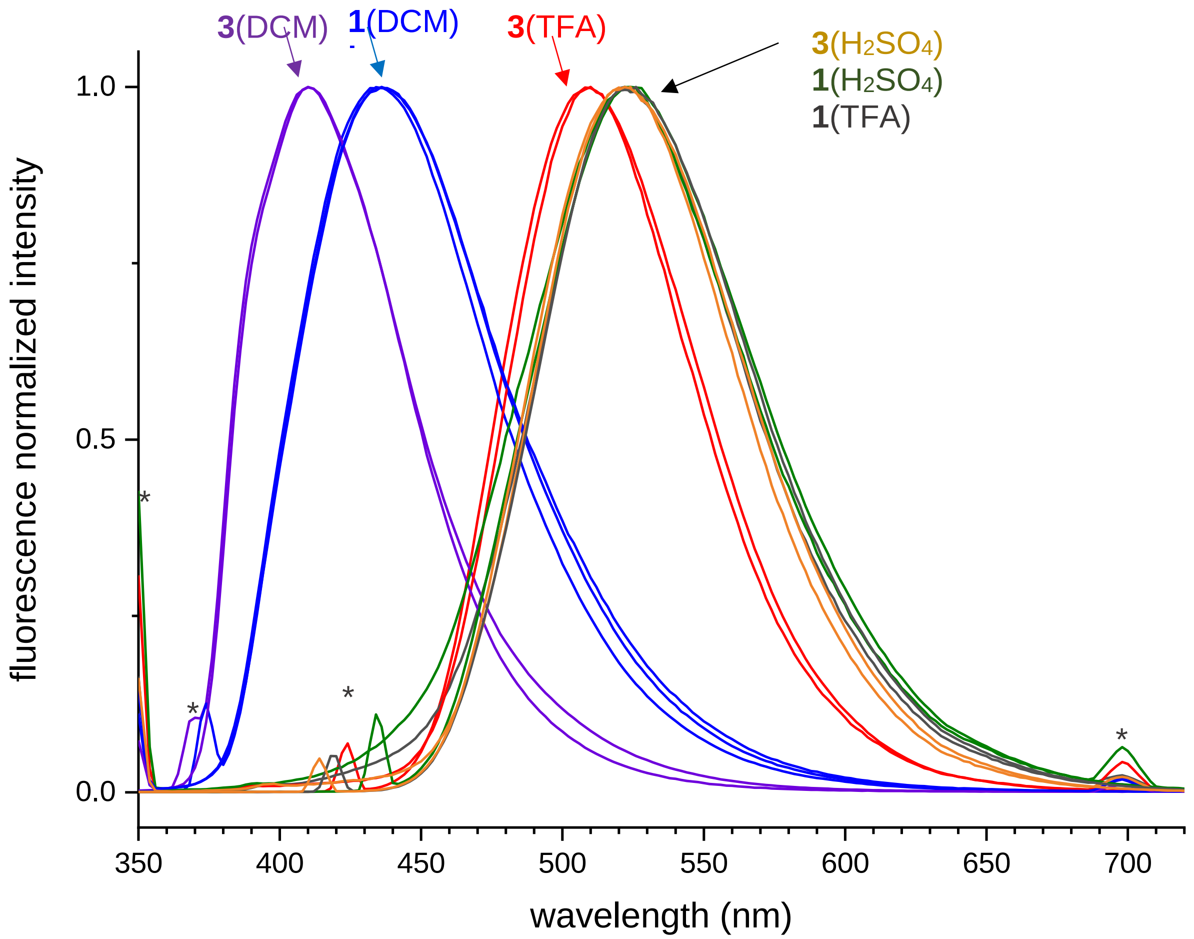

Figure S25. Comparison of emission spectra obtained at different excitation wavelengths of compounds **1** and **3** in DCM, DCM/TFA, and aqueous H_2SO_4. *Scattering artifacts are marked with asterisk.

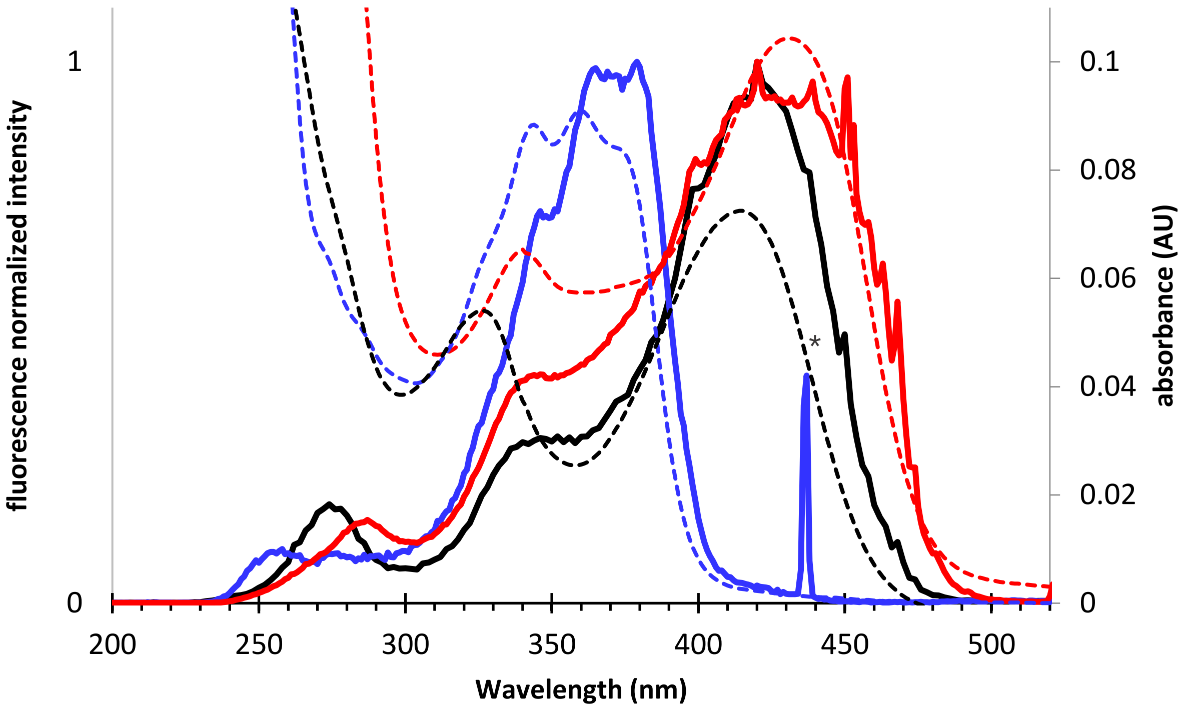

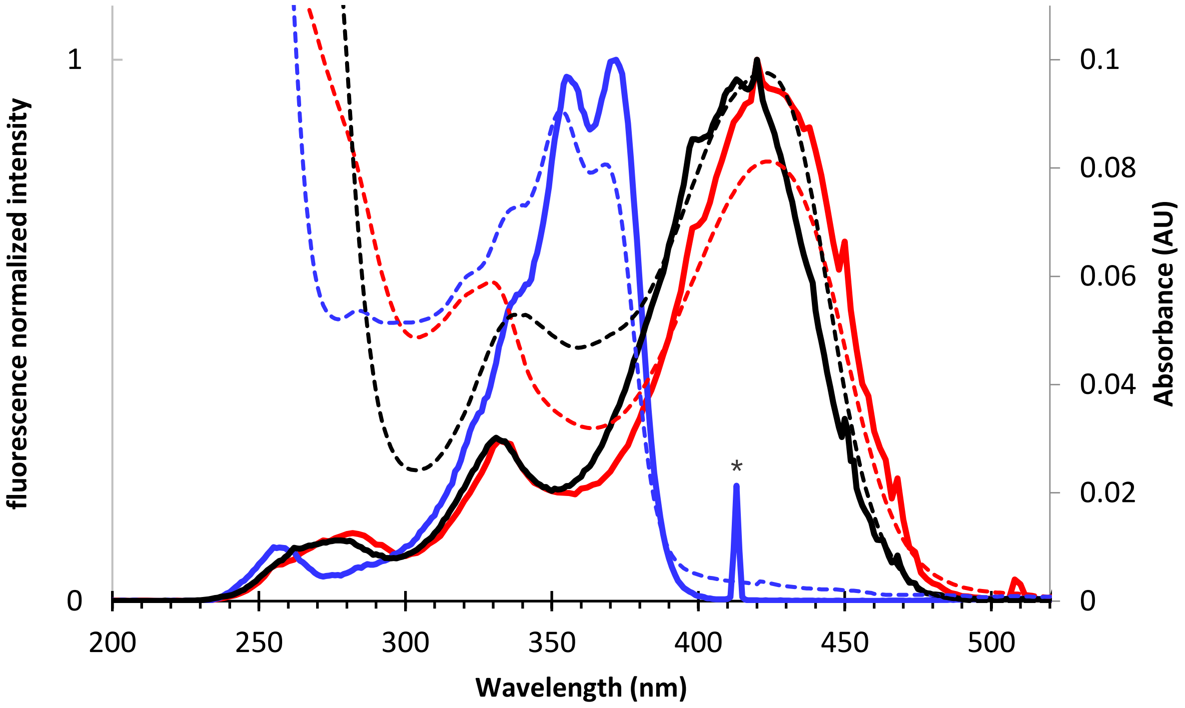

Figure S26. Overlay of UV absorbance (dashed lines) and uncorrected excitation spectra (solid lines) for 10^{-5} M solutions of **1** (top) and **3** (bottom) in DCM (blue lines), DCM/TFA (red lines) and H_2O/H_2SO_4 (black lines) for. (excitation spectrum for **1** in DCM/TFA is shown for 10^{-4} M solution). *Scattering artifacts are marked with asterisk.

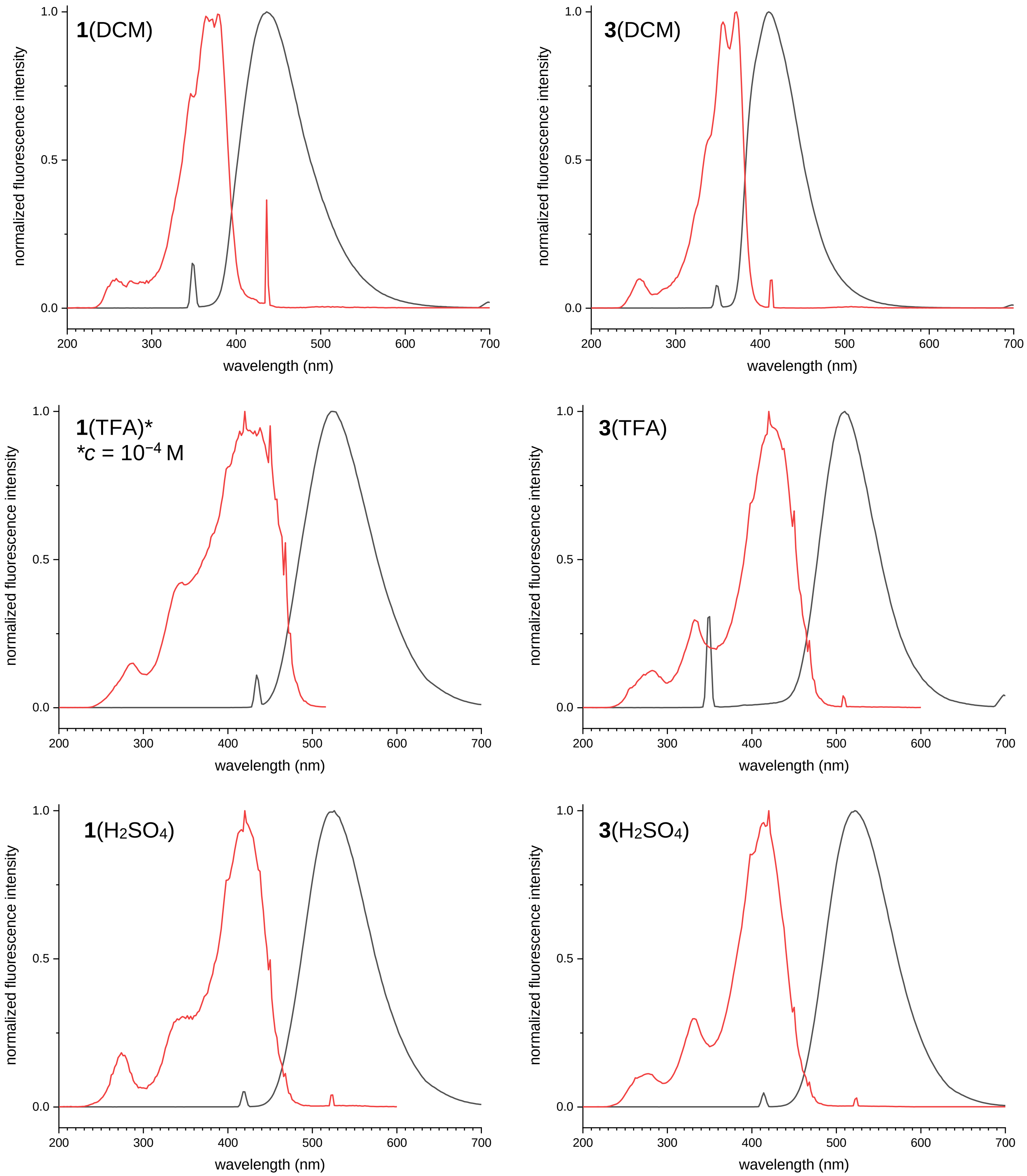

Figure S27. Excitation (red lines) and emission (black lines) for 10^{-5} M solutions of **1** and **3**. Spectra for **1** in DCM/TFA are shown for 10^{-4} M solution

S9. Microscopic staining experiments

A solution of **1** (ca. 1 mg) in DMSO (0.5 mL) was diluted with 0.9% saline water (10 mL) and a specimen was placed in the solution. The specimen was washed with plenty of water and placed on a microscope slide. Microscope observation was performed using bright field and with suboptimal fluorescence setting (setup for FITC, excitation wavelength 460-490 nm / long pass 515 nm filter) (Figures S28-S29).

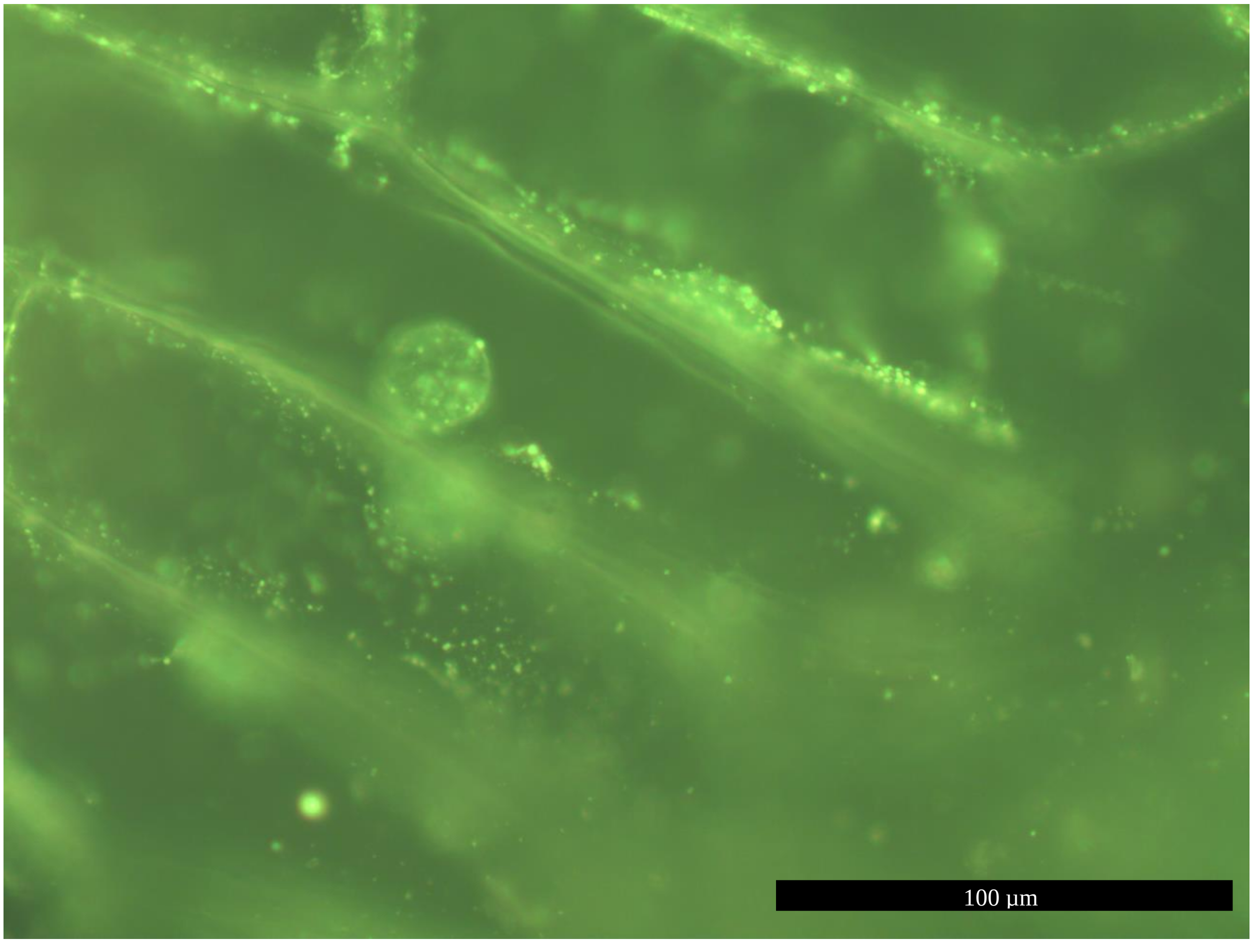

Figure S28. Fluorescent (EX 460-490 nm) light micrograph of live plant skin from onion (*Alium ceba L.*) stained with a solution of **1** in DMSO/water taken with Plan-achromat lens 40×/0.65. This is an expanded and uncropped version of Figure 3 from the main text. Scale bar represents 100 µm. For a wider field of view at lower magnification, see the following Figure S28.

Figure S29. (↓on the following page). Light micrographs of the same section of live plant skin from onion (*Alium ceba L.*) stained with a solution of **1** in DMSO/water taken with Plan-achromat lens 20×/0.4: bright field micrography (top) and fluorescent micrography (bottom) using top illumination at 460-490 nm. Scale bar represents 100µm

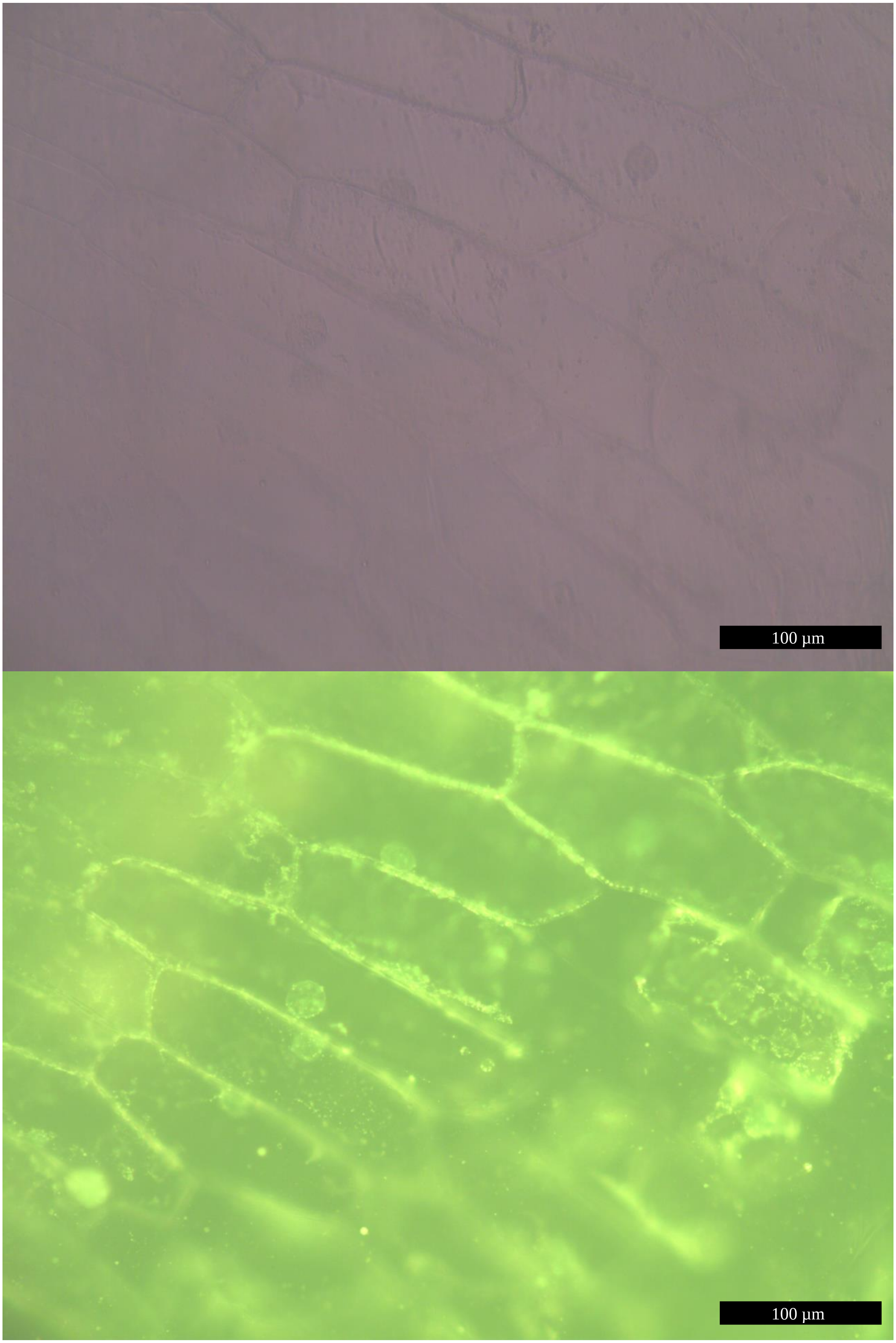

Figure caption is on the preceding page

S10. Plots of NMR spectra

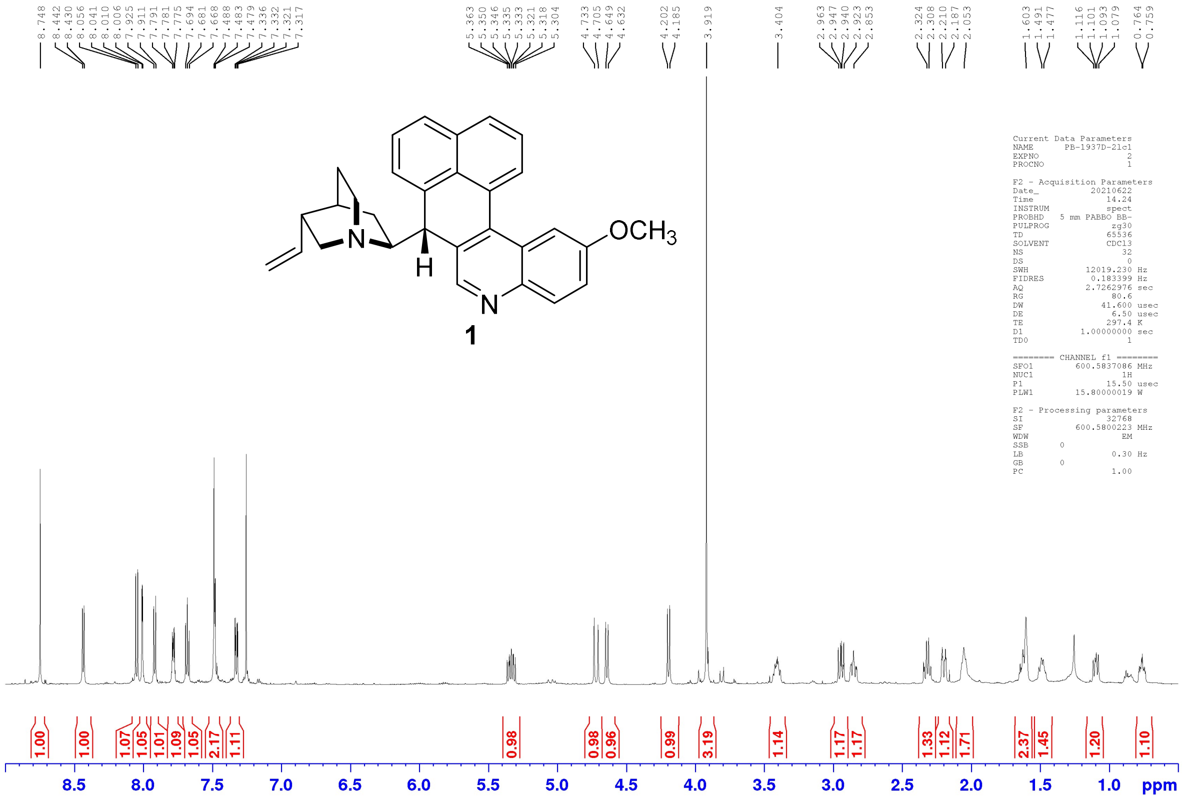

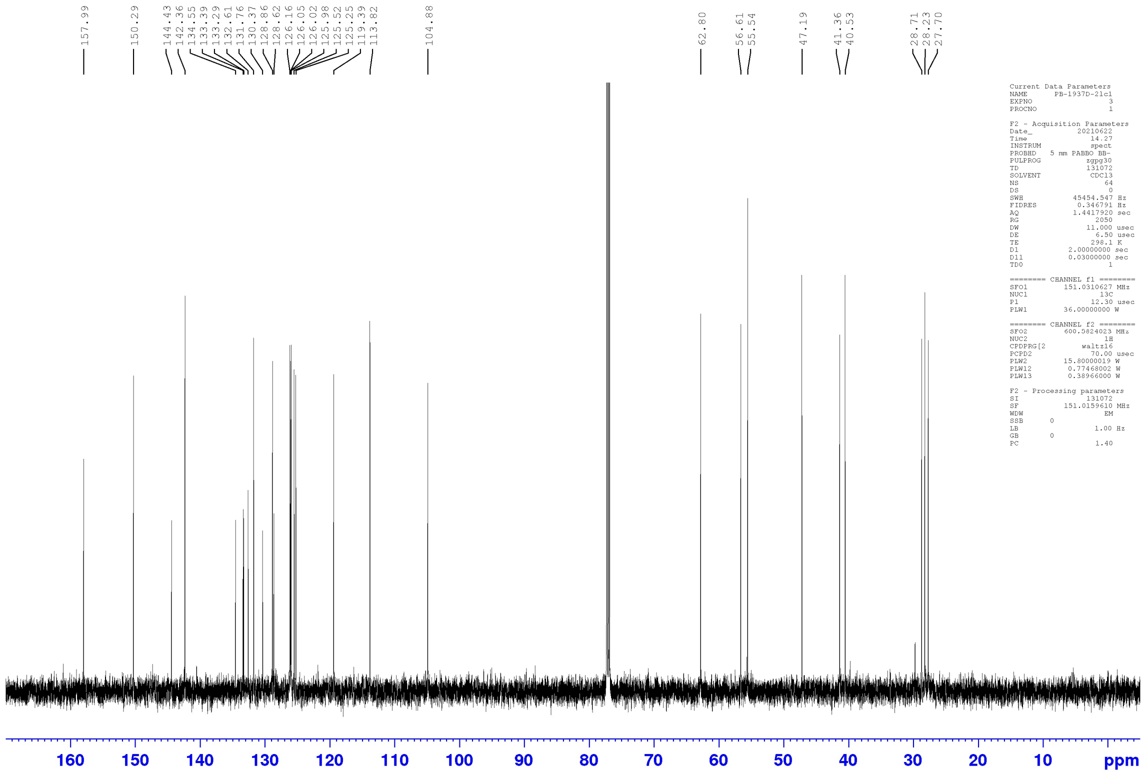

Figure S30. ^{1}H (600 MHz) and ^{13}C{^{1}H} (151 MHz) NMR spectra of **1** in $CDCl_3$.

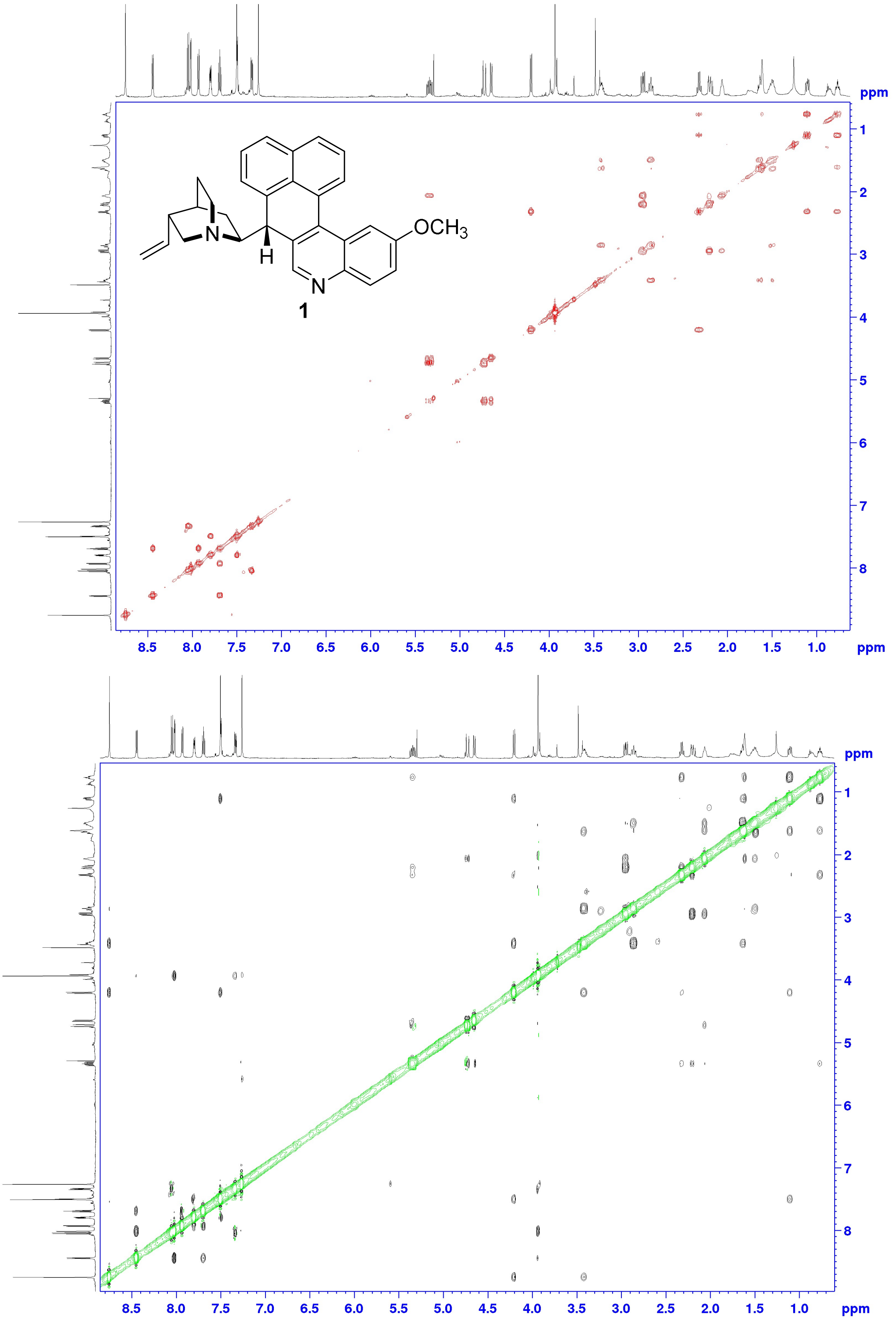

Figure S31. ^{1}H COSY (top) and NOESY (bottom) NMR experiments (600 MHz) for **1** in $CDCl_3$.

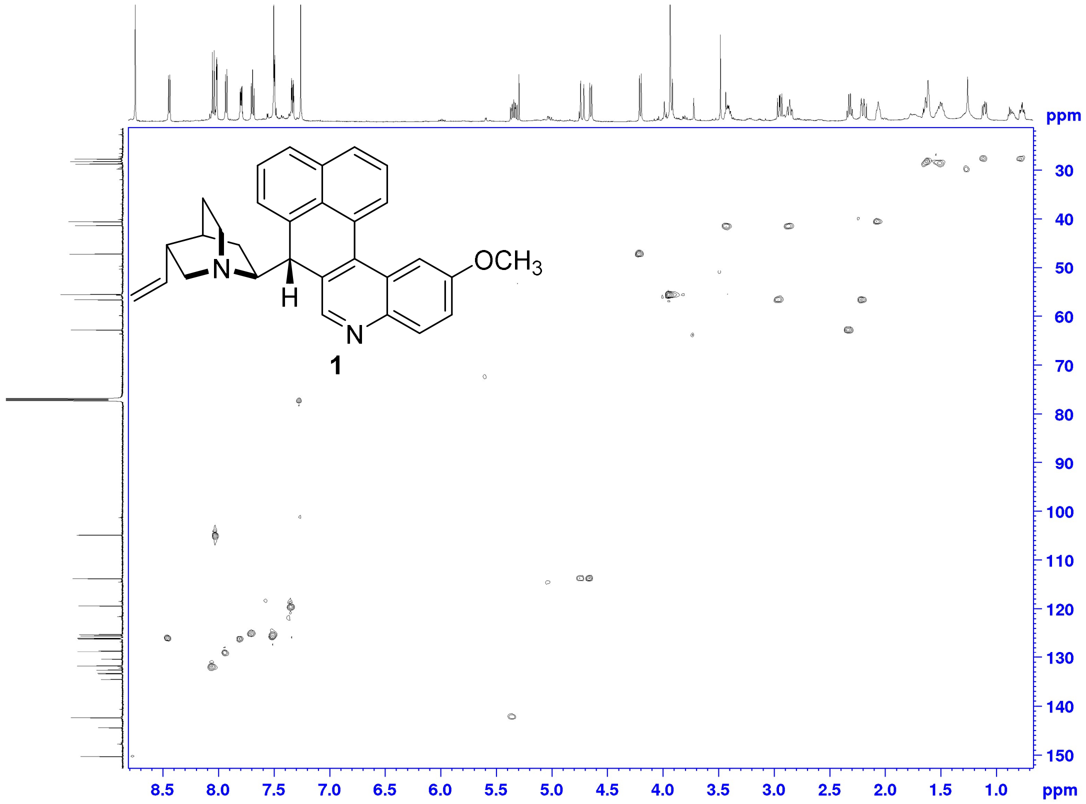

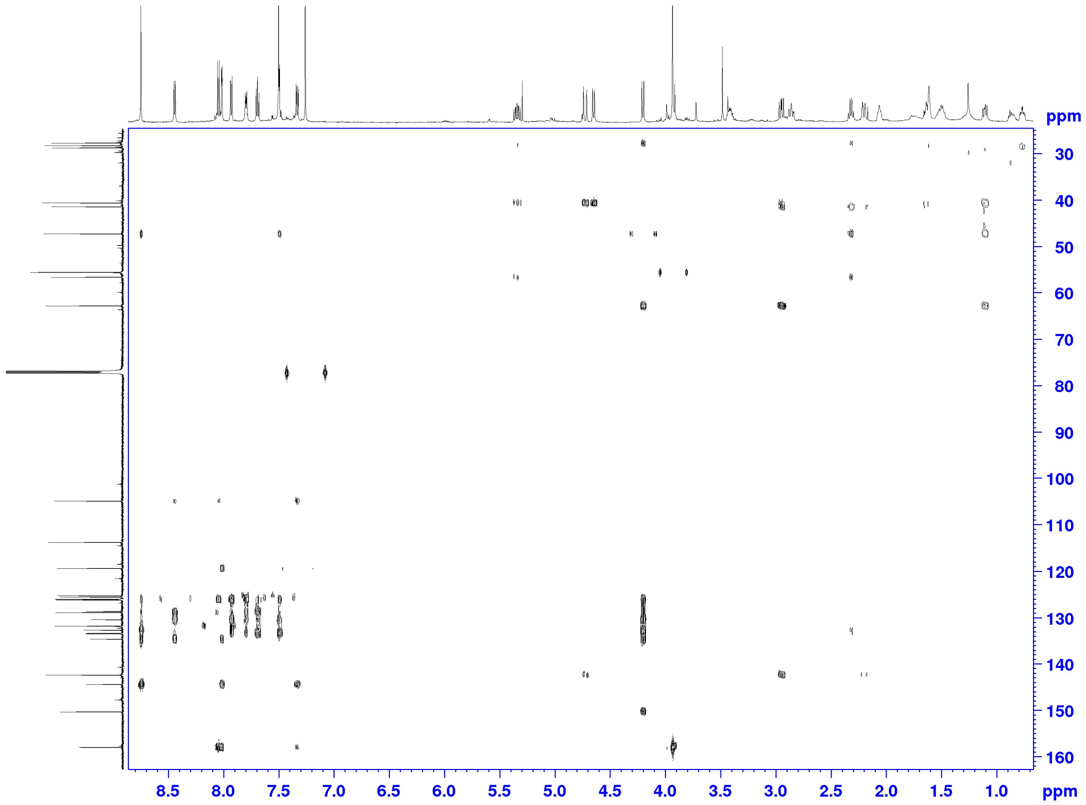

Figure S32. ^{1}H,^{13}C HSQC (top) and HMBC (bottom) NMR experiments (600/151 MHz) for **1** in $CDCl_3$.

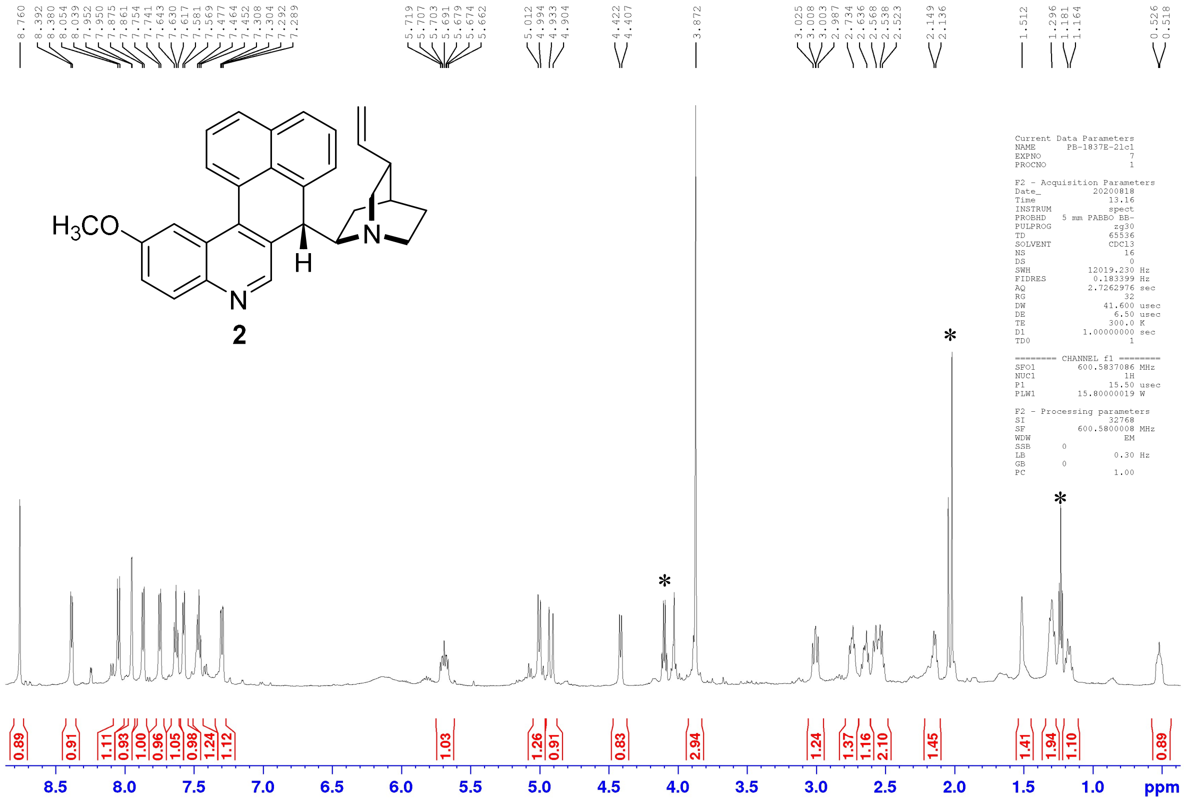

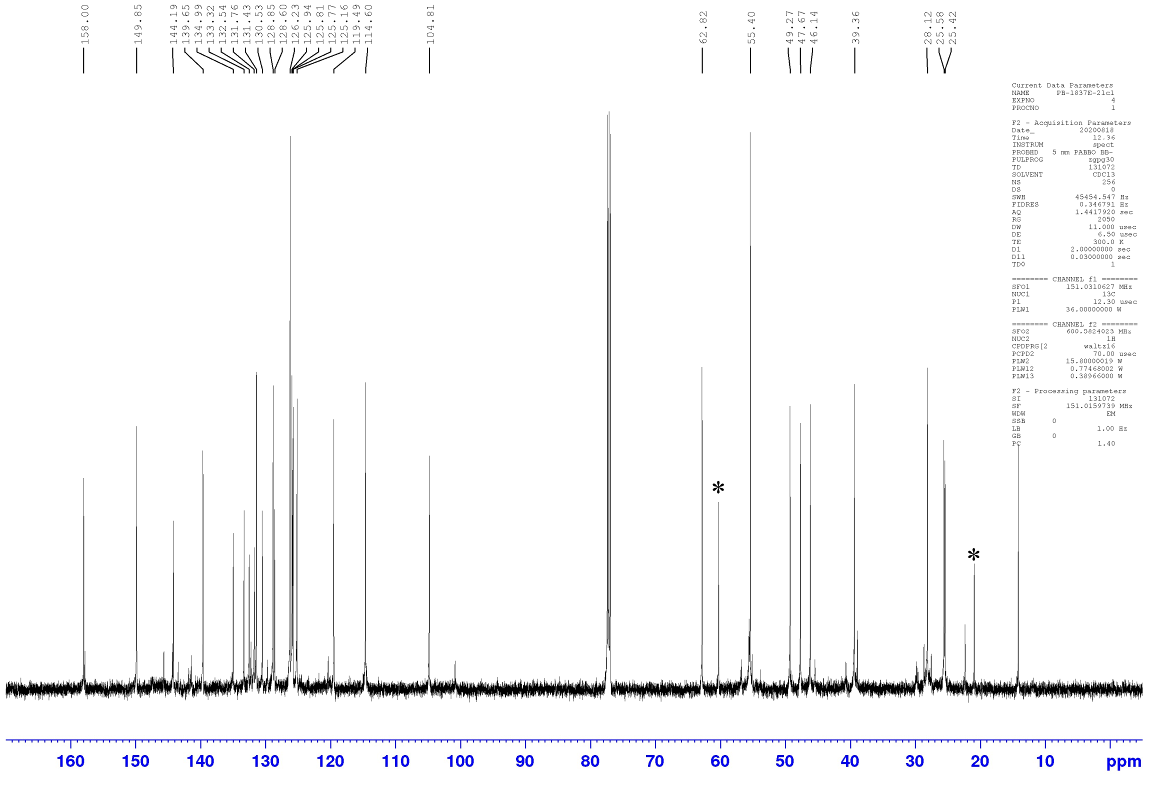

Figure S33. ^{1}H (600 MHz) and $^{13}C\{^{1}H\}$ (151 MHz) NMR spectra of **2** in $CDCl_3$. (*trace of EtOAc remained in the sample, 9% by integration)

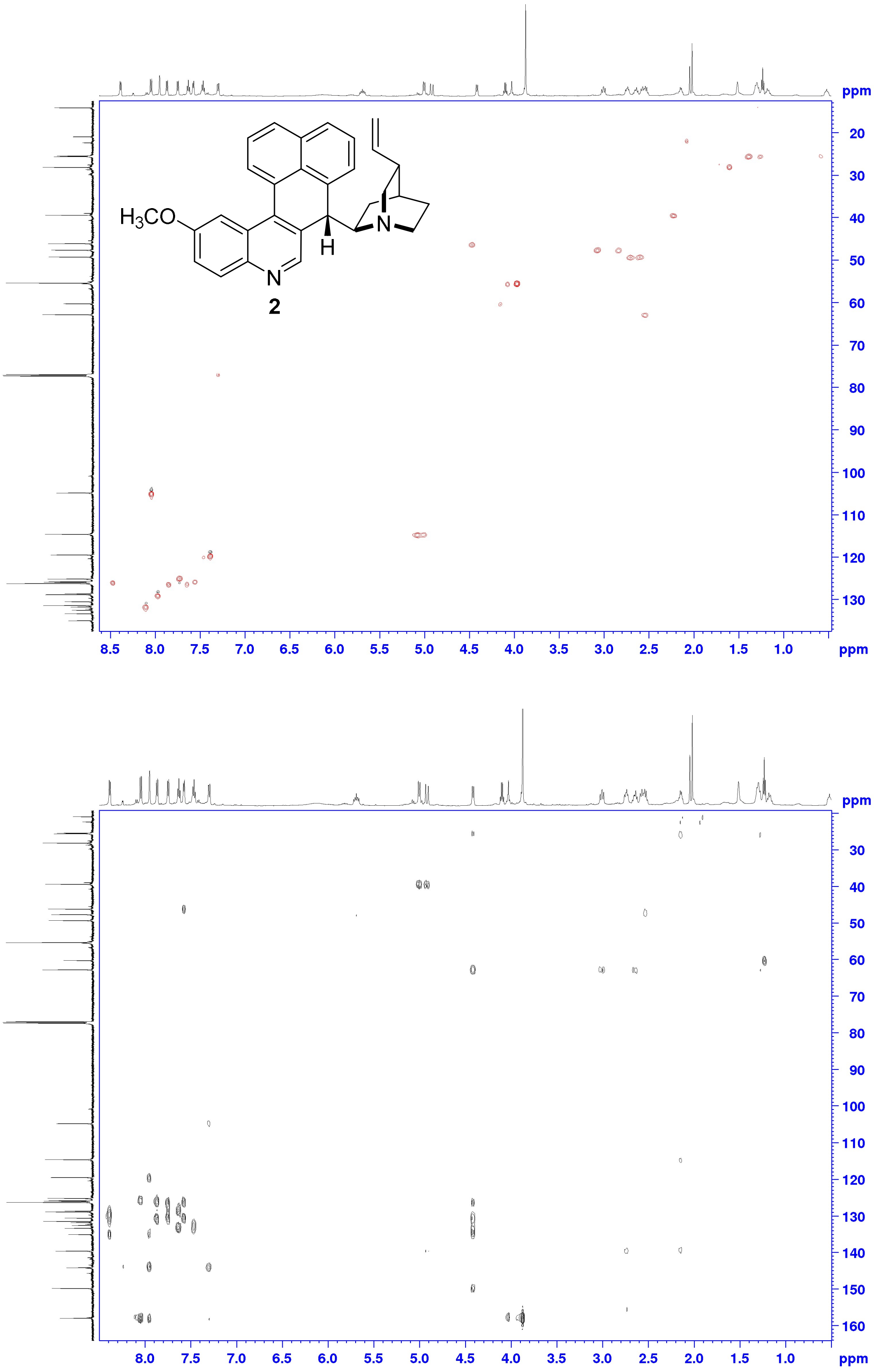

Figure S34. ^{1}H,^{13}C HSQC (top) and HMBC (bottom) NMR experiments (600/151 MHz) for **2** in $CDCl_3$.

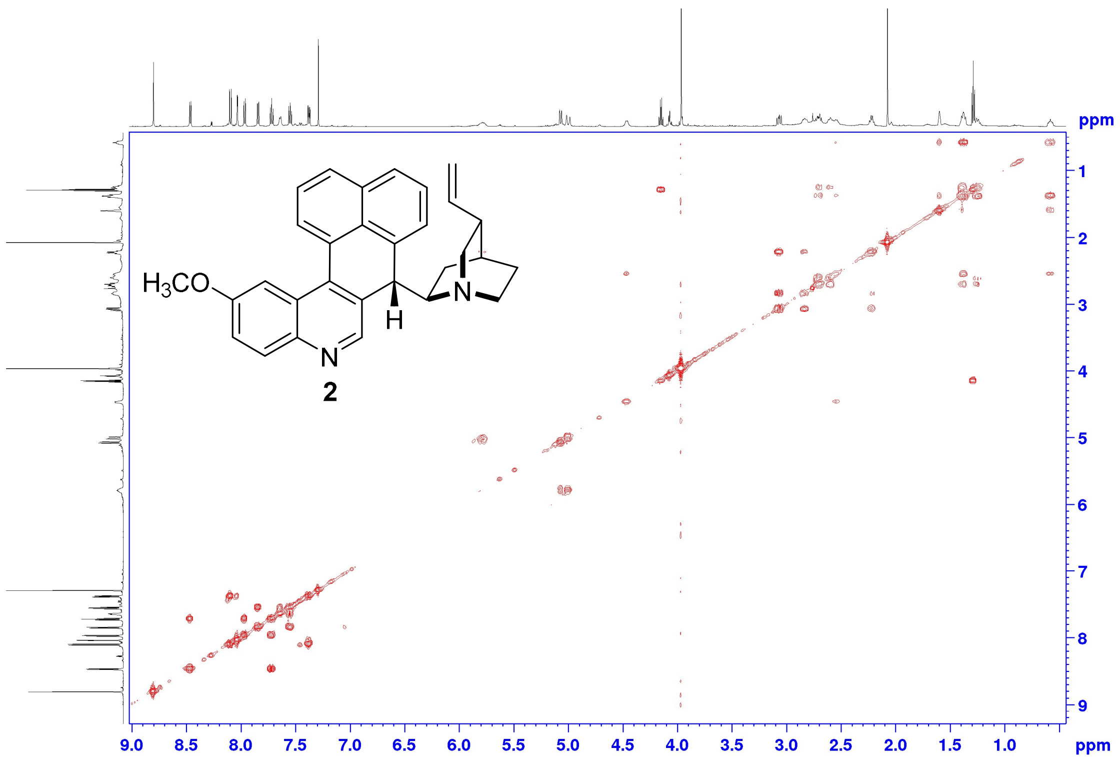

Figure S35. ^{1}H COSY NMR experiment (600 MHz) for **2** in $CDCl_3$.

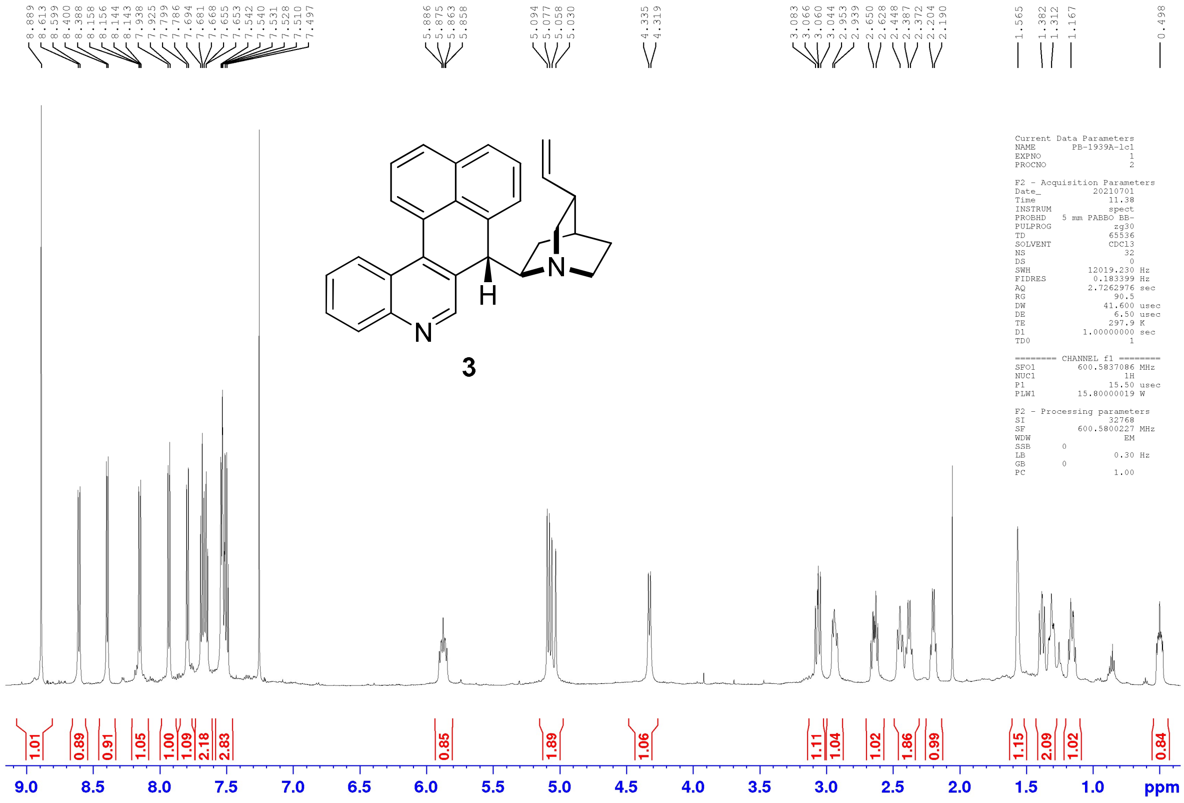

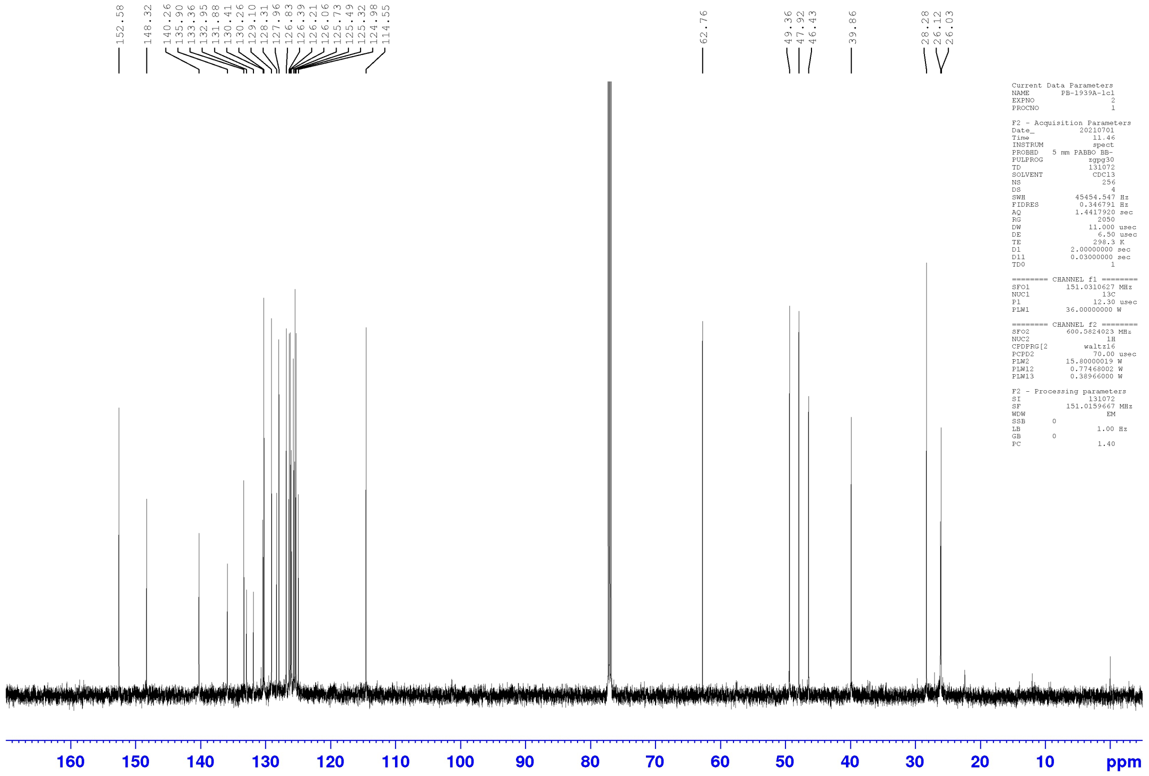

Figure S36. ^{1}H (600 MHz) and ^{13}C{^{1}H} (151 MHz) NMR spectra of **3** in $CDCl_3$.

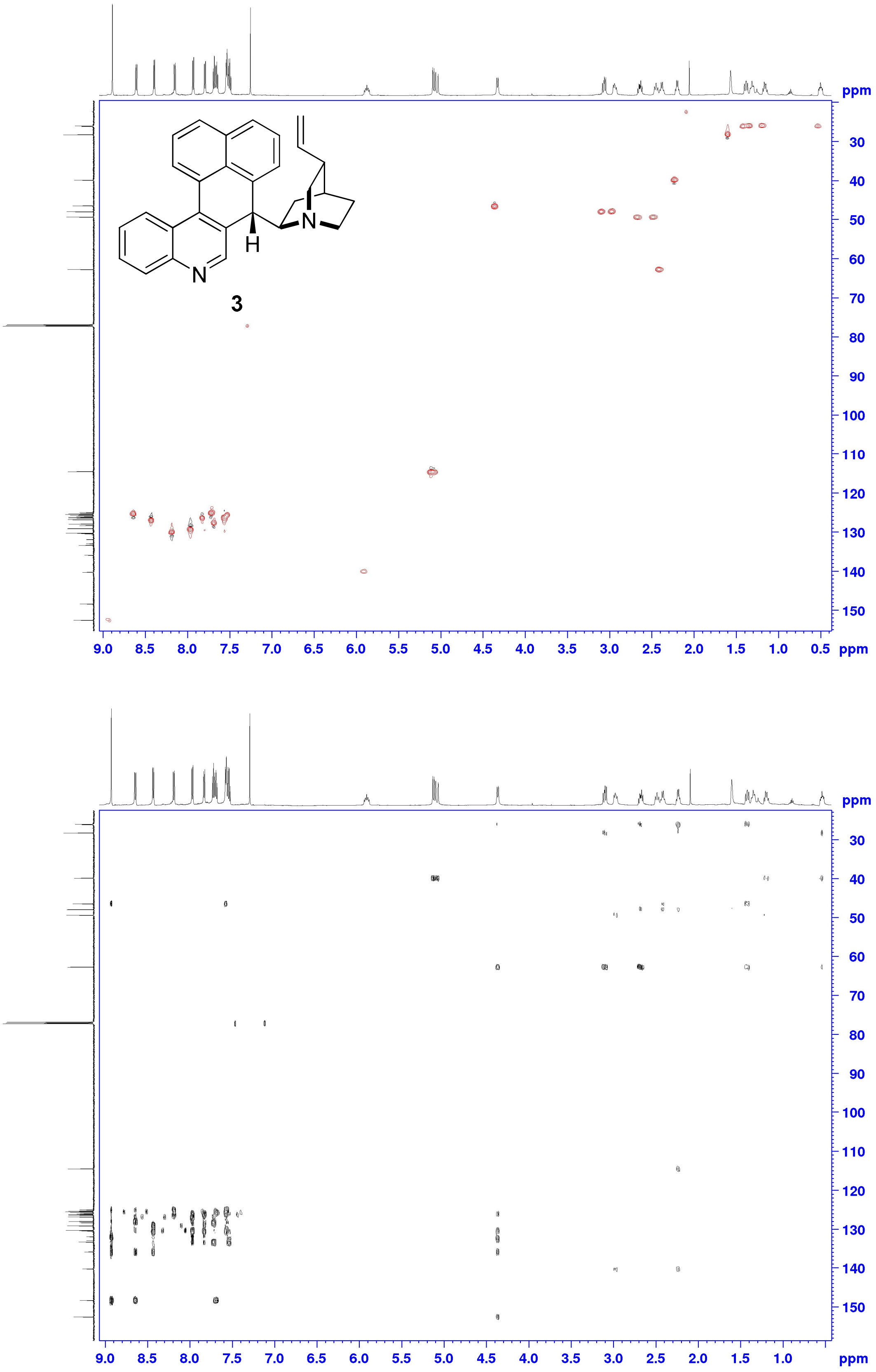

Figure S37. $^{1}H,^{13}C$ HSQC (top) and HMBC (bottom) NMR experiments (600/151 MHz) for **3** in $CDCl_3$.

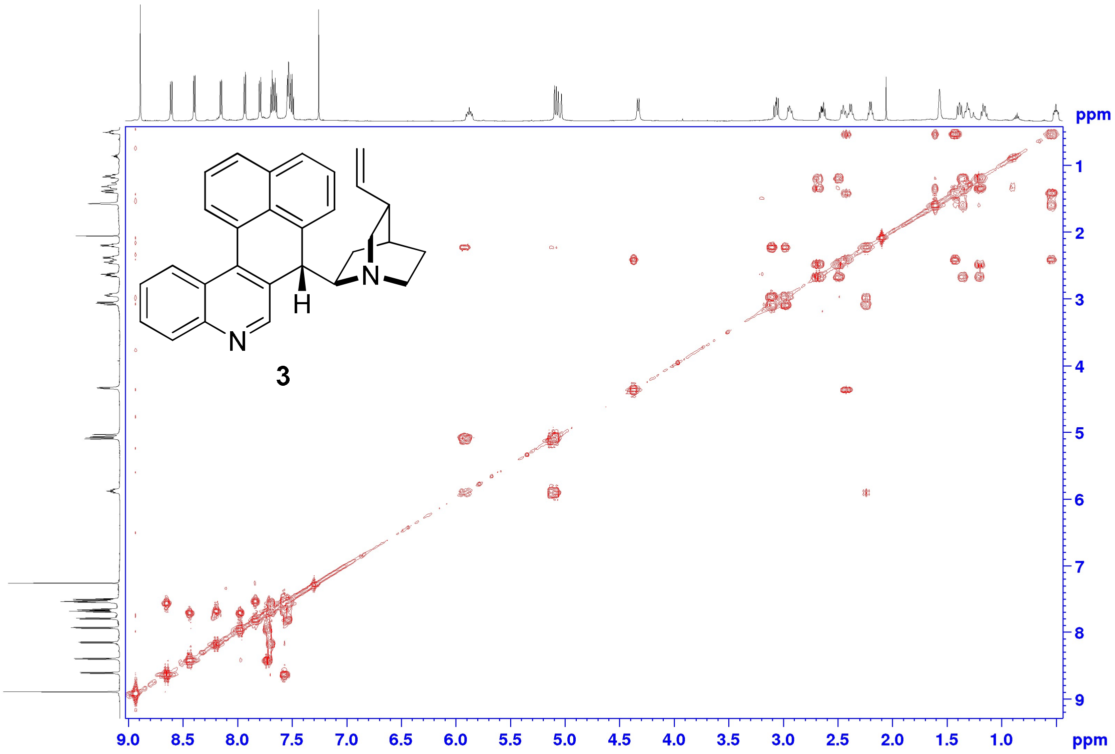

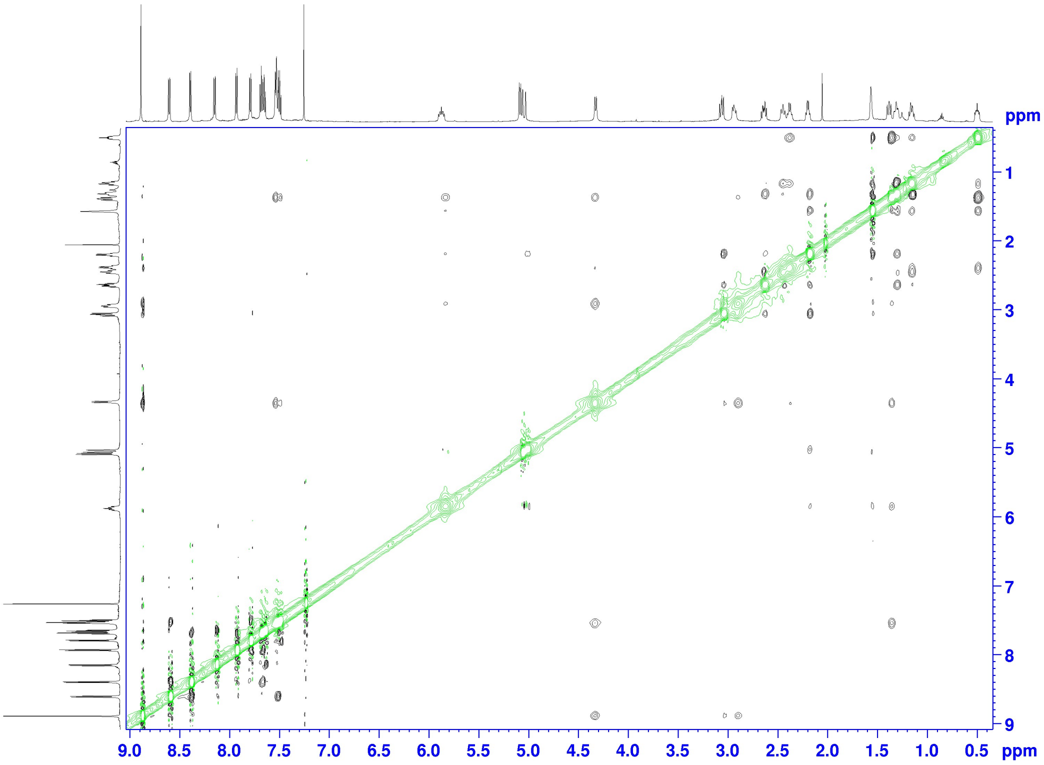

Figure S38. H COSY (top) and NOESY (bottom) NMR experiments (600 MHz) for **3** in $CDCl_3$.

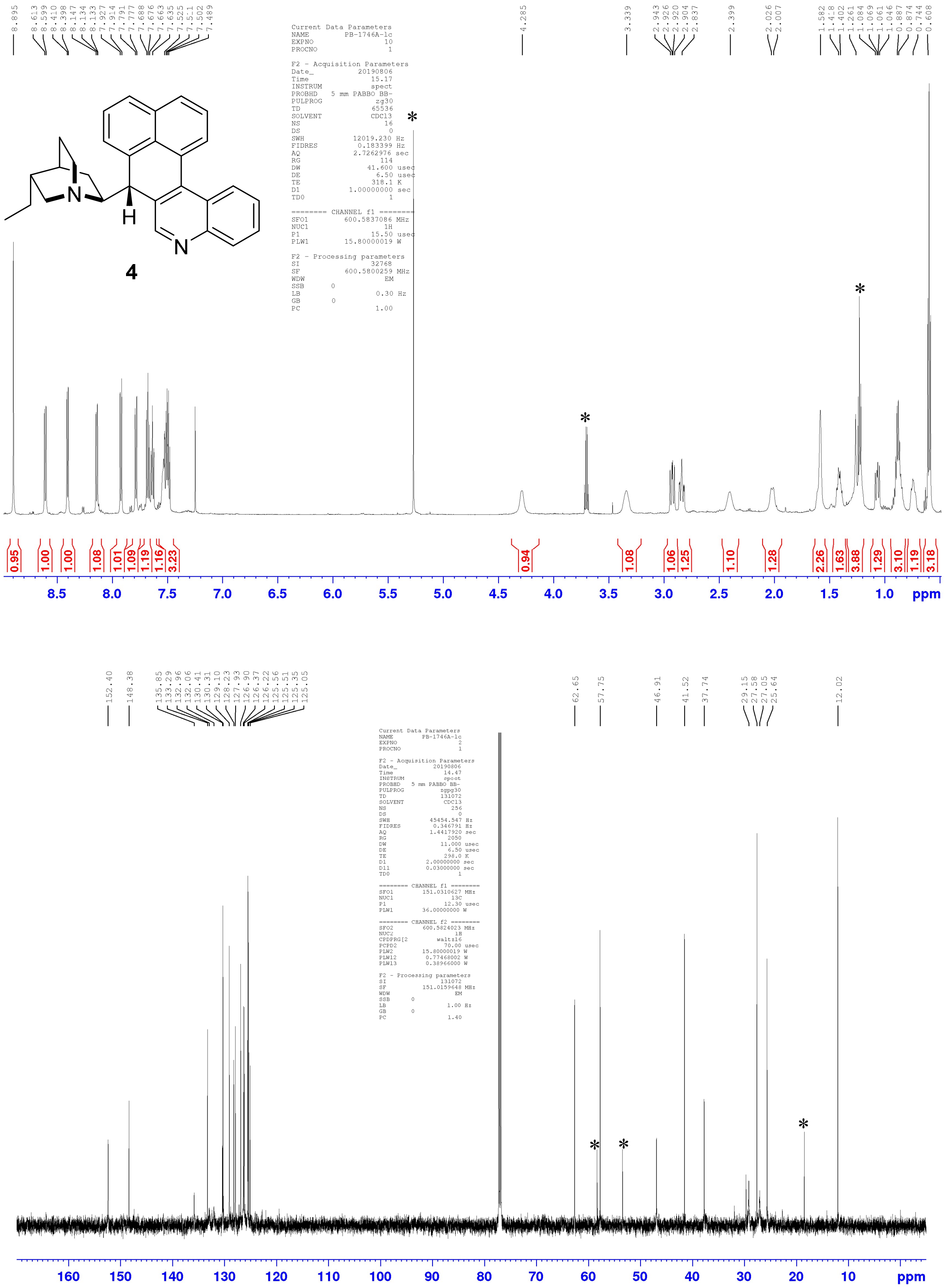

Figure S39. ^{1}H (600 MHz) and ^{13}C{^{1}H} (151 MHz) NMR spectra of **4** in $CDCl_3$. (*trace of EtOH and CH_2Cl_2 remained in the sample, 11% by integration)

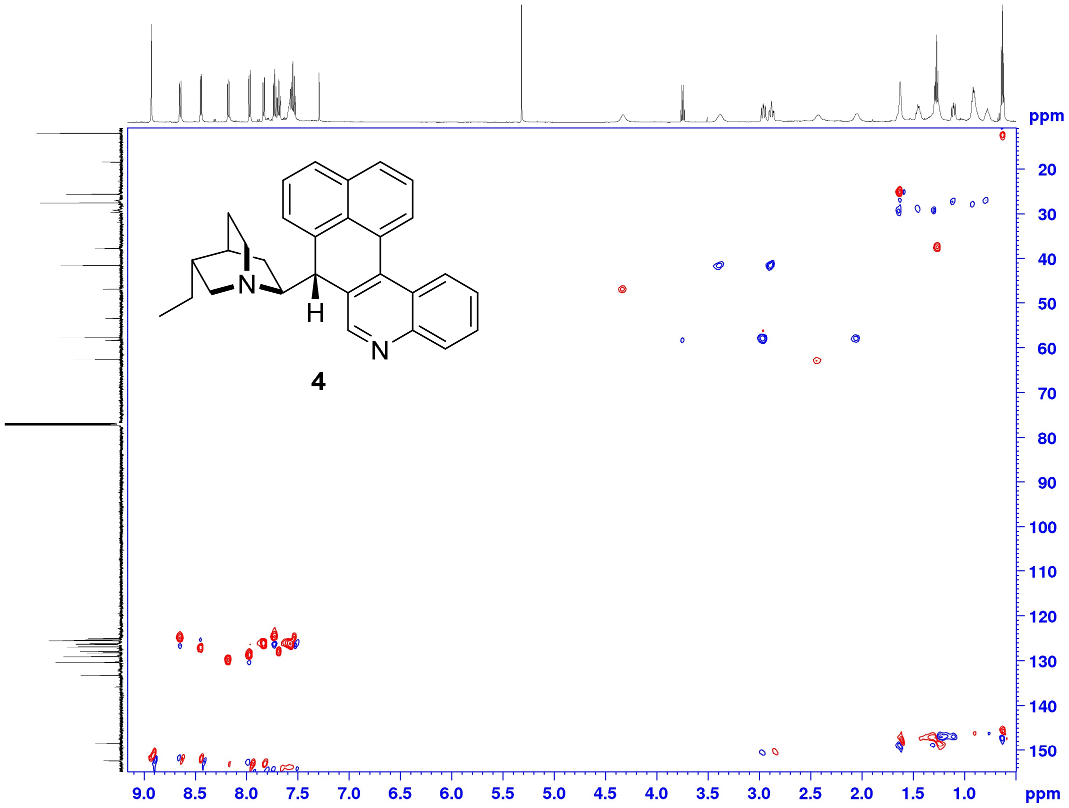

Figure S40. Multiplicity-edited ^{1}H,^{13}C HSQC experiment (600/151 MHz) for **4** in $CDCl_3$. Correlations of CH/CH_3 (red) and CH_2 (blue) are revealed in opposite phase.

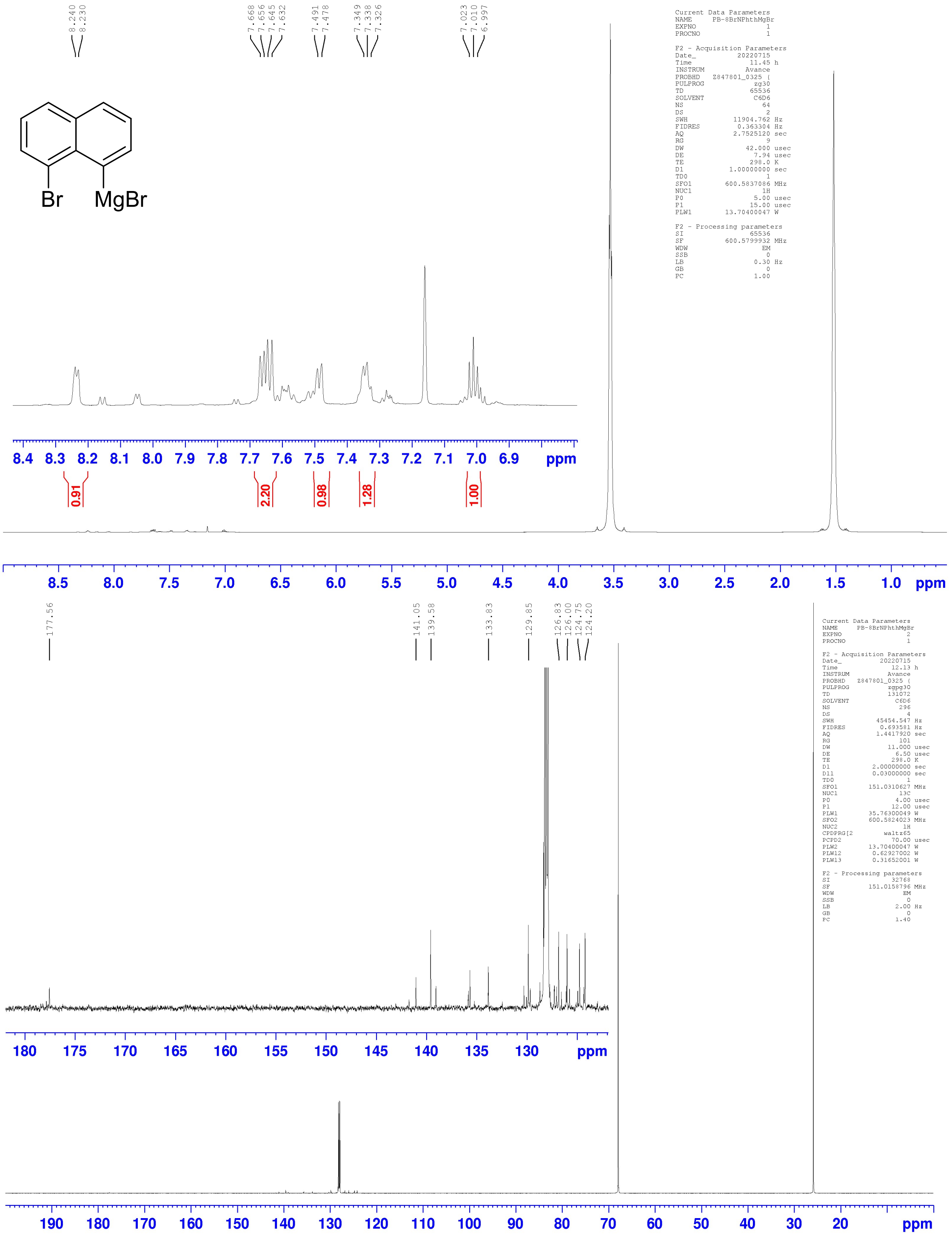

Figure S41. ^{1}H (600 MHz) and ^{13}C{^{1}H} (151 MHz) NMR spectra of a 0.14 M solution of 8-bromo-1-naphthylmagnesium bromide in THF (non-deuterated) / benzene-d_6 1:1 v/v. Sample was prepared with partial exclusion of air and moisture. Spectra were internally calibrated for benzene-d_5 (δ_H = 7.16 ppm) and benzene-d_6 (δ_C = 128.06 ppm). Inset plots show magnified sections containing signals of the products. Signals of major component (approx. 65%) are labeled and integrated. See the following Figure S42 for help with identification of minor species.

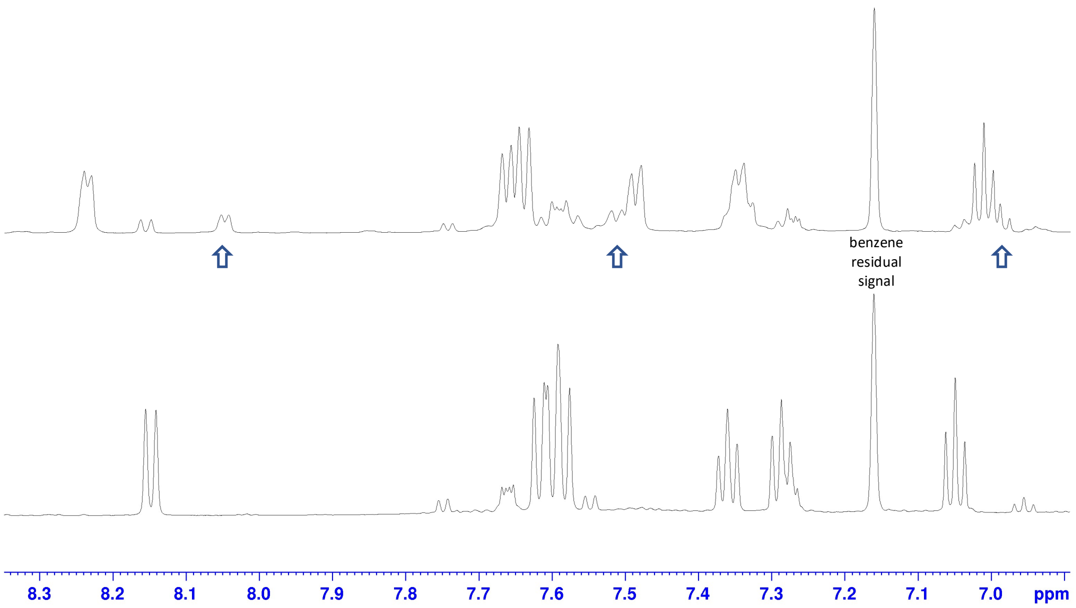

Figure S42. ^{1}H (600 MHz) and $^{13}C\{^{1}H\}$ (151 MHz) NMR spectra of a 0.14 M solution of 8-bromo-1-naphthylmagnesium bromide in THF (non-deuterated) / benzene-d_6 1:1 v/v (expanded version of Figure S41) – top plot, and the spectrum of the same sample after exposition to air via a punctured septum for 4 days, displaying mostly 1-bromonaphthalene – bottom plot. Distinct signals of disappearing minor organometallic species are marked with blue arrows.

S11. Computational details

All calculations were performed using Gaussian 16 code.[S10] Geometries of **1** and its (7*R*)-epimer were evaluated assuming two major degrees of freedom: rotation along the C7-C2' bond (or C8-C9 according to traditional numbering) affording 3 possible conformations and relative tilt of two aromatic rings that is *M* and *P* transient helicity (Figure S43). From these 6 geometries optimizations were carried at DFT/B3LYP/CC-pVDZ level of theory. For product of 7*R* configuration the lowest energy structure (7*R*,*P*)-**1** was by 4.6 kcal/mol lower in energy than (7*R*,*M*)-**1**. For the unobserved isomer (7*S*)-**1** two lowest energy conformations of **1** were within 3 kcal/mol (7*S*,*P*)-**1** and (7*S*,*M*)-**1**. All structures converged to minimum as confirmed by no imaginary frequencies.

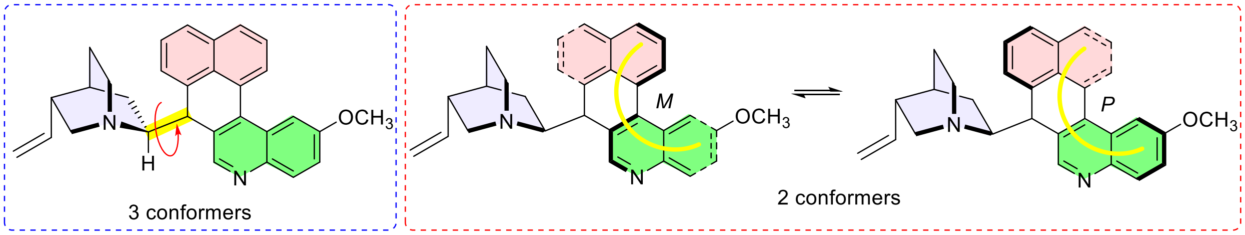

Figure S43. Depiction of major degrees of freedom for rearranged products.

For the computed gas-phase geometries of **1** GIAO calculations using mPW1PW91/6-311+G(2d,p) in SMD (Solvation Model Based on Density) solvent model (benzene and chloroform) were further calculated. The results were shown in Tables S1 and S2.

For compound **3**, models of 7*S* and 7*R* epimers were first optimized at the DFT/B3LYP/6-31+G(d,p) and subsequent GIAO calculations using mPW1PW91/6-311+G(d,p) in PCM (Polarizable Continuum Model) solvent model for chloroform. Only one lowest energy structure was considered.

Simplified radical cations **Int.C**$^{+\bullet}$ – **Int.F**$^{+\bullet}$ (magnesium ion replaced by proton) and their parent neutral radicals **Int.B**$^{\bullet}$ – **Int.F**$^{\bullet}$ were optimized at the unrestricted DFT/B3LYP/CC-pVDZ level of theory and further optimized at unrestricted M06-2X level with the same basis set. Calculations were performed both in the gas phase and SMD solvent models. For neutral radicals, toluene and THF models were used and relative energies showed insignificant differences. For radical cations, THF model was used. Treatment of transient stereogenic centers emerging in hydro-pyridine ring at positions 6 and 6a (IUPAC numbering, *cf*. Figure S1) in **Int.E*r***$^{\bullet}$, and **Int.F*r***$^{+\bullet}$: initial models were constructed observing *syn*-orientation for ring closures followed by optimization of lowest energy stereoisomers. Atom coordinates and computed energies are listed for one isomer.

[S10] Gaussian 16, Revision C.01, M. J. Frisch, G. W. Trucks, H. B. Schlegel, G. E. Scuseria, M. A. Robb, J. R. Cheeseman, G. Scalmani, V. Barone, G. A. Petersson, H. Nakatsuji, X. Li, M. Caricato, A. V. Marenich, J. Bloino, B. G. Janesko, R. Gomperts, B. Mennucci, H. P. Hratchian, J. V. Ortiz, A. F. Izmaylov, J. L. Sonnenberg, D. Williams-Young, F. Ding, F. Lipparini, F. Egidi, J. Goings, B. Peng, A. Petrone, T. Henderson, D. Ranasinghe, V. G. Zakrzewski, J. Gao, N. Rega, G. Zheng, W. Liang, M. Hada, M. Ehara, K. Toyota, R. Fukuda, J. Hasegawa, M. Ishida, T. Nakajima, Y. Honda, O. Kitao, H. Nakai, T. Vreven, K. Throssell, J. A. Montgomery, Jr., J. E. Peralta, F. Ogliaro, M. J. Bearpark, J. J. Heyd, E. N. Brothers, K. N. Kudin, V. N. Staroverov, T. A. Keith, R. Kobayashi, J. Normand, K. Raghavachari, A. P. Rendell, J. C. Burant, S. S. Iyengar, J. Tomasi, M. Cossi, J. M. Millam, M. Klene, C. Adamo, R. Cammi, J. W. Ochterski, R. L. Martin, K. Morokuma, O. Farkas, J. B. Foresman, and D. J. Fox, Gaussian, Inc., Wallingford CT, 2016.

(*unlike*) (7*R*,*P*)-**1**

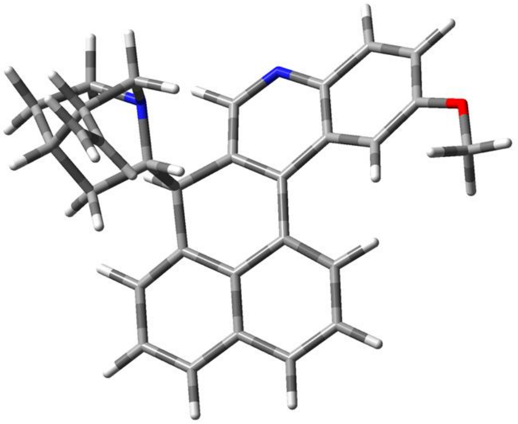

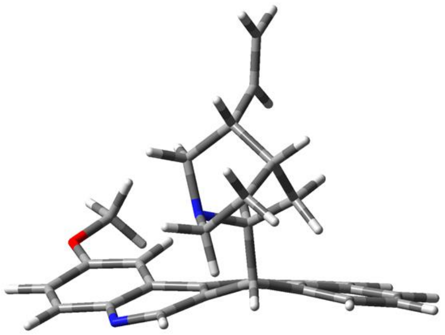

RB3LYP / CC-pVDZ
Imaginary Freq = 0
Dipole Moment = 3.4475191 Debye
Polarizability (α) = 355.514 a.u.

Electronic Energy (EE) =	-1344.8244	Hartree
Zero-point Energy Correction =	0.509644	Hartree
Thermal Correction to Energy =	0.535313	Hartree
Thermal Correction to Enthalpy =	0.536257	Hartree
Thermal Correction to Free Energy =	0.453672	Hartree
EE + Zero-point Energy =	-1344.3148	Hartree
EE + Thermal Energy Correction =	-1344.2891	Hartree
EE + Thermal Enthalpy Correction =	-1344.2882	Hartree
EE + Thermal Free Energy Correction =	-1344.3708	Hartree

E (Thermal) = 335.914 kcal/mol
Heat Capacity (Cv) = 106.893 cal/mol-kelvin
Entropy (S) = 173.815 cal/mol-kelvin

Atomic coordinates:

C	1.138442	1.936310	-0.927036
C	0.018293	2.438892	-0.198749
C	0.057906	3.800830	0.258561
C	1.190772	4.609451	-0.028839
H	1.205471	5.638758	0.337605
C	2.226046	4.119295	-0.792924
H	3.075572	4.755762	-1.049951
C	2.186800	2.783975	-1.250492
H	3.004357	2.410392	-1.870743
C	1.173330	0.476156	-1.307650
H	1.727258	0.367800	-2.253847
C	-1.267709	0.303076	-0.640695
C	-0.210679	-0.112308	-1.456517
C	-0.407728	-1.181285	-2.364956
N	-1.519039	-1.873358	-2.485216
C	-2.489342	-1.629641	-1.559308
C	-3.622782	-2.492212	-1.580407
H	-3.658669	-3.236791	-2.377090
C	-4.597350	-2.417950	-0.618945
H	-5.458438	-3.088076	-0.611054
C	-4.463474	-1.475812	0.434928
C	-3.402191	-0.583955	0.454623
C	-2.405008	-0.594591	-0.567704
O	-5.435768	-1.551245	1.388972
C	-5.381381	-0.651252	2.482879
H	-5.440593	0.400497	2.148893
H	-6.254055	-0.881020	3.108149
H	-4.461691	-0.787000	3.079995
C	1.926776	-0.367169	-0.207030
H	1.281435	-0.308502	0.683951

C	2.222336	-2.569176	0.667787
H	1.361499	-2.435182	1.341451
H	2.260802	-3.637403	0.403144
C	3.558315	-2.131172	1.378404
H	4.229024	-3.003534	1.459278
C	4.243611	-1.109147	0.426866
H	5.208773	-0.798820	0.855817
C	3.349520	0.126808	0.184684
H	3.307536	0.779881	1.068971
H	3.805684	0.727553	-0.618177
C	4.453967	-1.801534	-0.931907
H	5.031079	-2.731724	-0.799639
C	3.044701	-2.091630	-1.529476
H	2.944827	-3.146026	-1.831625
H	2.869045	-1.492013	-2.435382
N	1.972715	-1.802608	-0.560413
C	-1.178113	1.646667	-0.003716
C	-2.263655	2.273126	0.606382
C	-2.198621	3.593056	1.105768
C	-1.057878	4.343690	0.951199
H	-1.005887	5.372071	1.316641
H	-3.079259	4.019004	1.591741
H	0.402156	-1.436649	-3.056411
H	-3.285651	0.075976	1.306517
H	-3.222183	1.769158	0.660305
H	5.041942	-1.151628	-1.601080
C	3.332858	-1.603194	2.768641
C	3.904042	-2.087011	3.876472
H	2.632444	-0.763196	2.865944
H	3.693240	-1.662983	4.861537
H	4.603872	-2.928345	3.837632

(*unlike*) (7*R*,*M*)-**1**

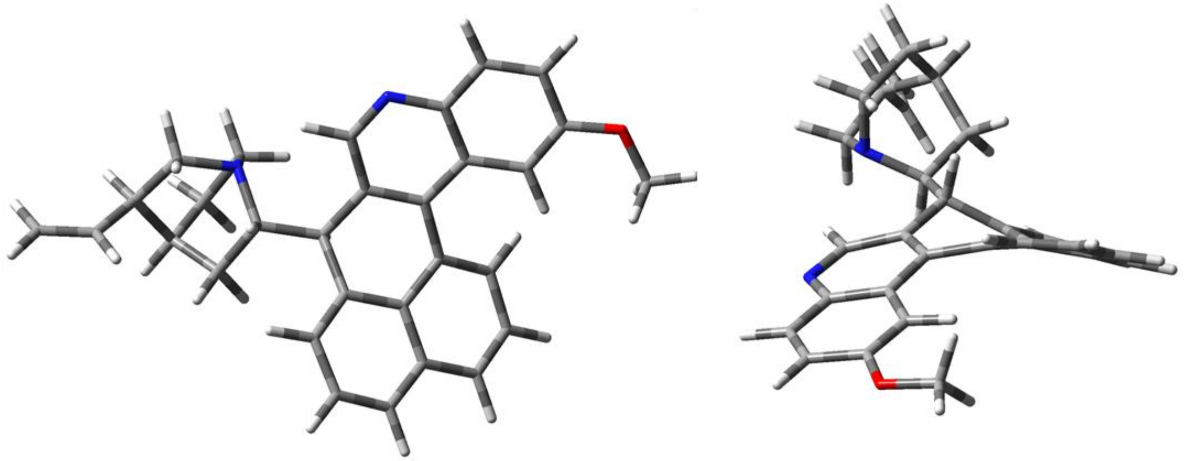

B3LYP / CC-pVDZ
Imaginary Freq = 0
Dipole Moment = 3.8051034 Debye
Polarizability (α) = 357.93333 a.u.

Electronic Energy (EE) =	-1344.8177	Hartree
Zero-point Energy Correction =	0.509639	Hartree
Thermal Correction to Energy =	0.535209	Hartree
Thermal Correction to Enthalpy =	0.536153	Hartree
Thermal Correction to Free Energy =	0.454377	Hartree
EE + Zero-point Energy =	-1344.308	Hartree
EE + Thermal Energy Correction =	-1344.2825	Hartree
EE + Thermal Enthalpy Correction =	-1344.2815	Hartree
EE + Thermal Free Energy Correction =	-1344.3633	Hartree

E (Thermal) = 335.849 kcal/mol
Heat Capacity (Cv) = 106.868 cal/mol-kelvin
Entropy (S) = 172.111 cal/mol-kelvin
Atomic coordinates:

C	-0.577475	1.601794	-0.529388
C	0.676926	2.102042	-0.064181
C	0.974397	3.494892	-0.166436
C	0.112803	4.318484	-0.945550
H	0.348135	5.379909	-1.055150
C	-0.999497	3.776587	-1.549596
H	-1.644600	4.399857	-2.172880
C	-1.367222	2.425637	-1.309946
H	-2.309506	2.067406	-1.721636
C	-0.971639	0.243785	0.060022
H	-1.000623	0.451667	1.149795
C	1.490014	-0.231379	0.167989
C	0.207474	-0.717601	-0.125076
C	0.065980	-2.060358	-0.560269
N	1.065745	-2.913392	-0.690587
C	2.321559	-2.457761	-0.445017
C	3.391293	-3.381958	-0.634296
H	3.117964	-4.401489	-0.910524
C	4.698821	-2.995719	-0.501418
H	5.527873	-3.688318	-0.655835
C	5.000197	-1.641027	-0.189918
C	3.987142	-0.723915	0.030145
C	2.613649	-1.109339	-0.052235
O	6.333799	-1.356135	-0.146037
C	6.728643	-0.024923	0.140029
H	6.352228	0.685263	-0.617948
H	7.826403	-0.023854	0.123626

H	6.379652	0.298022	1.137590
C	-2.403587	-0.224537	-0.308643
H	-2.456420	-0.322710	-1.406245
C	-2.769546	-1.599188	1.690026
H	-1.760048	-1.402590	2.079850
H	-3.018957	-2.630333	1.985701
C	-3.812049	-0.585466	2.247794
H	-3.321294	0.205734	2.838848
H	-4.533951	-1.083698	2.915556
C	-4.540234	0.044077	1.046147
H	-5.309562	0.751396	1.392456
C	-3.502455	0.788187	0.183903
H	-3.053155	1.595578	0.783825
H	-4.002114	1.277976	-0.665344
C	-5.211652	-1.097461	0.233859
H	-5.823287	-1.686896	0.938124
C	-4.036784	-1.989084	-0.305979
H	-4.198291	-3.043524	-0.033264
H	-3.991981	-1.945087	-1.406222
N	-2.730116	-1.571892	0.218149
C	-6.120883	-0.617206	-0.862297
H	-5.648306	-0.047313	-1.673449
C	-7.436314	-0.847494	-0.928165
H	-7.956114	-1.418058	-0.151488
H	-8.041564	-0.477194	-1.759472
C	1.631299	1.203811	0.525247
C	2.670325	1.758606	1.266391
C	2.882480	3.157776	1.297047
C	2.099629	4.003933	0.538404
H	2.315654	5.074219	0.493178
H	3.708472	3.558247	1.889094
H	-0.932118	-2.444326	-0.763916
H	4.238024	0.312033	0.226002
H	3.347938	1.112991	1.823531

(*like*) (7*S*,*P*)-**1**

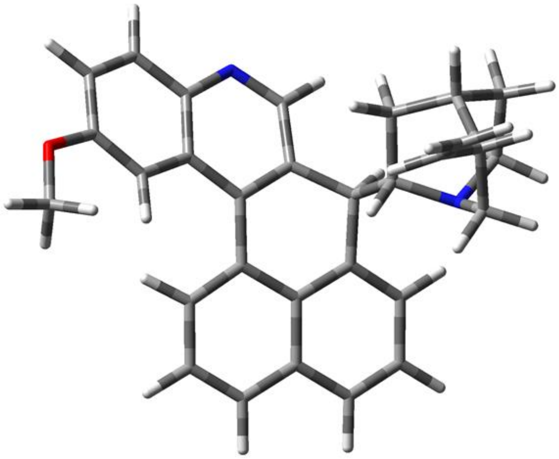

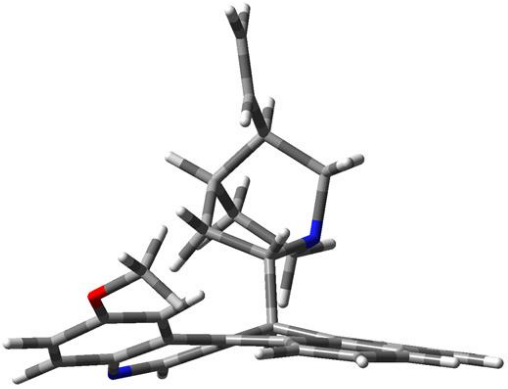

B3LYP / CC-pVDZ
Imaginary Freq = 0
Dipole Moment = 2.736533 Debye
Polarizability (α) = 356.75167 a.u.

Electronic Energy (EE) =	-1344.8251	Hartree
Zero-point Energy Correction =	0.509615	Hartree
Thermal Correction to Energy =	0.535274	Hartree
Thermal Correction to Enthalpy =	0.536218	Hartree
Thermal Correction to Free Energy =	0.453622	Hartree
EE + Zero-point Energy =	-1344.3154	Hartree
EE + Thermal Energy Correction =	-1344.2898	Hartree
EE + Thermal Enthalpy Correction =	-1344.2888	Hartree
EE + Thermal Free Energy Correction =	-1344.3714	Hartree

E (Thermal) = 335.89 kcal/mol
Heat Capacity (Cv) = 106.902 cal/mol-kelvin
Entropy (S) = 173.839 cal/mol-kelvin
Atomic coordinates:

C	-1.152783	1.916625	-0.808913
C	-0.017487	2.417581	-0.105393
C	-0.072246	3.757032	0.414071
C	-1.243356	4.538609	0.225418
H	-1.268132	5.551480	0.634861
C	-2.313164	4.038050	-0.482931
H	-3.204046	4.648823	-0.645253
C	-2.260509	2.726709	-1.002453
H	-3.113447	2.336140	-1.557042
C	-1.164563	0.477593	-1.267431
H	-1.709935	0.399189	-2.221618
C	1.292347	0.322021	-0.639253
C	0.227396	-0.077648	-1.455573
C	0.452127	-1.066254	-2.446636
N	1.575748	-1.730829	-2.614256
C	2.540778	-1.535003	-1.672437
C	3.686974	-2.378461	-1.742724
H	3.732478	-3.078821	-2.578053
C	4.663447	-2.339072	-0.781495
H	5.534387	-2.995708	-0.811181
C	4.523211	-1.452030	0.319379
C	3.449134	-0.578419	0.389792
C	2.444885	-0.556999	-0.624957
O	5.503107	-1.559933	1.260956
C	5.444566	-0.715046	2.398593
H	5.485873	0.352229	2.115718
H	6.325461	-0.962505	3.005136

H	4.531643	-0.893868	2.994511
C	-1.945551	-0.388121	-0.201077
H	-1.506092	-0.105128	0.768776
C	-4.168838	-0.599407	-1.192932
H	-3.748846	-0.287595	-2.161514
H	-5.181191	-0.167952	-1.139717
C	-4.207478	-2.152233	-1.074140
H	-3.933871	-2.629274	-2.029621
H	-5.218703	-2.507496	-0.815180
C	-3.204985	-2.552480	0.023312
H	-3.118956	-3.647906	0.087280
C	-1.840349	-1.934000	-0.351442
H	-1.603607	-2.227890	-1.386085
H	-1.026088	-2.331601	0.272724
C	-3.734531	-1.993763	1.375166
H	-4.729275	-2.439377	1.546661
C	-3.891566	-0.438443	1.179975
H	-4.952959	-0.150098	1.239169
H	-3.363682	0.108910	1.976610
N	-3.366831	0.010480	-0.117061
C	-2.870441	-2.348952	2.554038
H	-1.856332	-1.928167	2.562561
C	-3.249928	-3.116237	3.581056
H	-4.251971	-3.555553	3.626251
H	-2.575815	-3.333328	4.413514
C	1.197712	1.643306	0.036874
C	2.290834	2.266365	0.637038
C	2.219860	3.570749	1.174206
C	1.058605	4.300452	1.081480
H	0.997091	5.313573	1.486311
H	3.107176	3.999371	1.645321
H	-0.347498	-1.275127	-3.167562
H	3.328117	0.042149	1.270580
H	3.256337	1.772293	0.654750

(*like*) (7*S*,*M*)-**1**

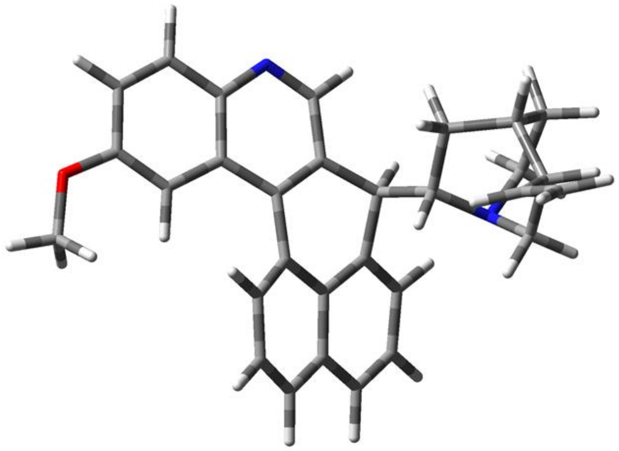

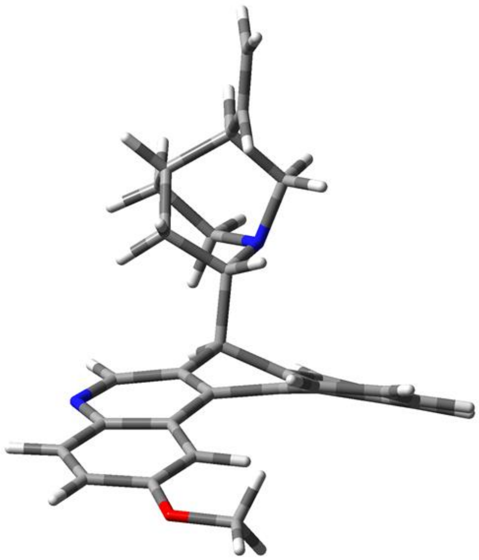

B3LYP / CC-pVDZ
Imaginary Freq = 0
Dipole Moment = 2.6558855 Debye
Polarizability (α) = 353.63367 a.u.

Electronic Energy (EE) =	-1344.8296	Hartree
Zero-point Energy Correction =	0.509515	Hartree
Thermal Correction to Energy =	0.535167	Hartree
Thermal Correction to Enthalpy =	0.536111	Hartree
Thermal Correction to Free Energy =	0.453559	Hartree
EE + Zero-point Energy =	-1344.3201	Hartree
EE + Thermal Energy Correction =	-1344.2944	Hartree
EE + Thermal Enthalpy Correction =	-1344.2935	Hartree
EE + Thermal Free Energy Correction =	-1344.376	Hartree

E (Thermal) = 335.822 kcal/mol
Heat Capacity (Cv) = 106.897 cal/mol-kelvin
Entropy (S) = 173.745 cal/mol-kelvin
Atomic coordinates:

C	-0.814048	1.524640	-1.287429
C	0.129687	2.046399	-0.355544
C	0.086824	3.437516	-0.017246
C	-0.778520	4.296448	-0.749331
H	-0.789094	5.362789	-0.510358
C	-1.603172	3.786408	-1.729394
H	-2.263547	4.450362	-2.291791
C	-1.647886	2.392292	-1.969602
H	-2.376763	1.993726	-2.676997
C	-1.005687	0.022799	-1.319727
H	-1.413994	-0.280607	-2.297026
C	1.328196	-0.147420	-0.334359
C	0.312040	-0.696627	-1.121289
C	0.523001	-1.947090	-1.753857
N	1.608142	-2.681763	-1.633959
C	2.634306	-2.152947	-0.911852
C	3.830673	-2.924267	-0.838978
H	3.812594	-3.908115	-1.310189
C	4.958488	-2.435367	-0.233496
H	5.885463	-3.008885	-0.183266
C	4.946074	-1.122775	0.312459
C	3.790523	-0.359477	0.291081
C	2.583838	-0.863085	-0.282272
O	6.141485	-0.713054	0.823846

C	6.228371	0.591299	1.374389
H	6.006680	1.368261	0.621147
H	7.264257	0.706076	1.719175
H	5.543385	0.717252	2.232159
C	-2.063876	-0.368065	-0.221564
H	-1.720048	0.123438	0.701977
C	-4.126092	-0.550181	-1.520445
H	-3.528763	-0.582584	-2.444384
H	-5.043921	0.008358	-1.764060
C	-4.454610	-1.987320	-1.015953
H	-4.130037	-2.747285	-1.745813
H	-5.539687	-2.121818	-0.873078
C	-3.713676	-2.186424	0.318744
H	-3.839136	-3.218640	0.679818
C	-2.219289	-1.890068	0.066711
H	-1.886895	-2.503218	-0.785720
H	-1.594106	-2.189921	0.921209
C	-4.325718	-1.197542	1.352193
H	-5.399758	-1.435980	1.436466
C	-4.162175	0.243683	0.737070
H	-5.149268	0.682741	0.522464
H	-3.658647	0.915338	1.450030
N	-3.386454	0.227646	-0.510625
C	-3.726821	-1.315072	2.726916
H	-2.666221	-1.047888	2.823412
C	-4.388347	-1.700996	3.822814
H	-5.448740	-1.971652	3.784335
H	-3.896964	-1.760127	4.797287
C	1.090934	1.182519	0.282538
C	1.765524	1.672622	1.396537
C	1.643955	3.024109	1.798834
C	0.873794	3.905415	1.071186
H	0.827745	4.963069	1.342452
H	2.196305	3.370151	2.675365
H	-0.268387	-2.356086	-2.393033
H	2.402886	1.011817	1.981245
H	3.813076	0.654138	0.673694

(*like*) (7*R*)-**3**

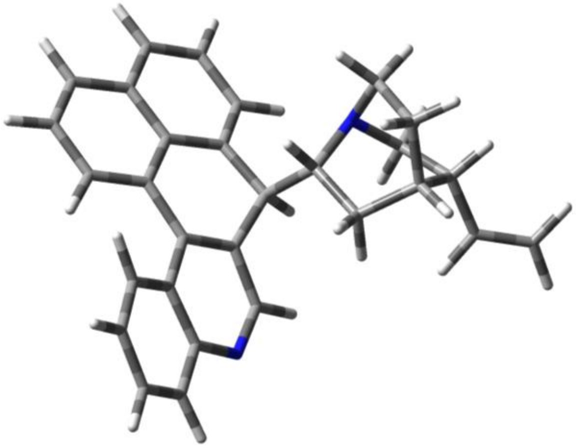

B3LYP / 6-31+G(d,p)
Imaginary Freq = 0
Dipole Moment = 2.7372565 Debye
Polarizability (α) = 366.792 a.u.

Electronic Energy (EE) =	-1230.3053	Hartree
Zero-point Energy Correction =	0.478443	Hartree
Thermal Correction to Energy =	0.501454	Hartree
Thermal Correction to Enthalpy =	0.502398	Hartree
Thermal Correction to Free Energy =	0.425883	Hartree
EE + Zero-point Energy =	-1229.8269	Hartree
EE + Thermal Energy Correction =	-1229.8039	Hartree
EE + Thermal Enthalpy Correction =	-1229.8029	Hartree
EE + Thermal Free Energy Correction =	-1229.8794	Hartree

E (Thermal) = 314.667 kcal/mol
Heat Capacity (Cv) = 97.81 cal/mol-kelvin
Entropy (S) = 161.039 cal/mol-kelvin
<u>Atomic coordinates:</u>

C	-0.008851	1.834134	-0.797039
C	-1.308262	2.067054	-0.259966
C	-1.758651	3.424269	-0.125048
C	-0.907592	4.490566	-0.518197
H	-1.262053	5.511175	-0.400511
C	0.329233	4.233796	-1.065538
H	0.969158	5.050394	-1.386845
C	0.770998	2.901435	-1.208667
H	1.751323	2.708358	-1.630074
C	0.532337	0.425348	-0.842680
H	1.170361	0.306358	-1.726659
C	-1.780752	-0.411434	-0.220578
C	-0.570536	-0.605395	-0.890389
C	-0.361395	-1.822051	-1.588434
N	-1.178813	-2.851493	-1.582820
C	-2.241949	-2.783888	-0.727709
C	-3.022674	-3.961454	-0.578047
H	-2.755205	-4.813167	-1.195123
C	-4.041740	-4.022521	0.345655
H	-4.621374	-4.932896	0.466497
C	-4.296342	-2.905000	1.172779
C	-3.586526	-1.731300	1.011693
C	-2.570119	-1.601991	0.021541
C	1.429964	0.169586	0.432884
H	0.804850	0.462418	1.284521
C	3.061787	1.159655	1.870844
H	2.316659	1.719613	2.445708
H	3.985430	1.749171	1.880197
C	3.293883	-0.264192	2.469237

H	2.499038	-0.526847	3.178243
H	4.241061	-0.310311	3.019645
C	3.301770	-1.268464	1.296143
H	3.582269	-2.267909	1.644243
C	1.897504	-1.300896	0.649298
H	1.189227	-1.838610	1.288629
H	1.938735	-1.855442	-0.293722
C	4.336435	-0.756091	0.254003
H	5.253218	-0.488978	0.796338
C	3.705119	0.545046	-0.356077
H	4.465909	1.326503	-0.461134
H	3.322410	0.344143	-1.362469
N	2.604243	1.068489	0.470657
C	4.711949	-1.758583	-0.799495
H	3.898973	-2.147753	-1.414831
C	5.955240	-2.187281	-1.043600
H	6.804652	-1.830574	-0.464586
H	6.165904	-2.908432	-1.827949
C	-2.201266	0.980987	0.079461
C	-3.494682	1.308566	0.478863
C	-3.918125	2.644610	0.645811
C	-3.065692	3.685409	0.364685
H	-3.388479	4.716871	0.478710
H	-4.934426	2.838741	0.975768
H	0.520489	-1.911064	-2.223114
H	-4.233680	0.535223	0.619676
H	-3.764258	-0.923100	1.708072
H	-5.044592	-2.972390	1.957318

(*unlike*) (7*S*)-**3**

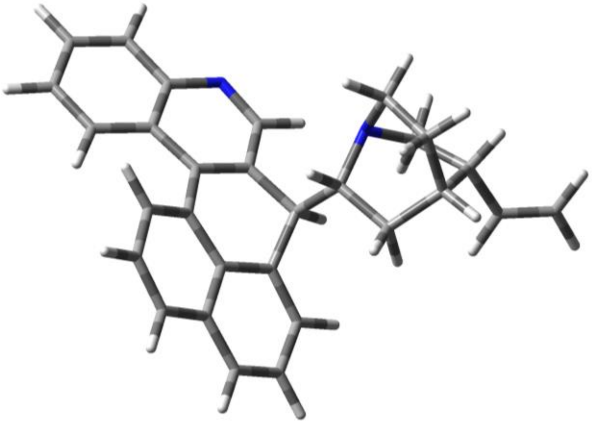

B3LYP / 6-31G(d,p)
Imaginary Freq = 0
Dipole Moment = 3.2982189 Debye
Polarizability (α) = 322.48267 a.u.

Electronic Energy (EE) =	-1230.2759	Hartree
Zero-point Energy Correction =	0.479558	Hartree
Thermal Correction to Energy =	0.502459	Hartree
Thermal Correction to Enthalpy =	0.503404	Hartree
Thermal Correction to Free Energy =	0.427432	Hartree
EE + Zero-point Energy =	-1229.7963	Hartree
EE + Thermal Energy Correction =	-1229.7734	Hartree
EE + Thermal Enthalpy Correction =	-1229.7725	Hartree
EE + Thermal Free Energy Correction =	-1229.8484	Hartree

E (Thermal) = 315.298 kcal/mol
Heat Capacity (Cv) = 97.446 cal/mol-kelvin
Entropy (S) = 159.895 cal/mol-kelvin

Atomic coordinates:

C	-0.230266	1.548891	-0.986899
C	0.866277	2.063032	-0.238440
C	0.996149	3.475861	-0.063166
C	0.132484	4.338503	-0.789567
H	0.247578	5.413007	-0.676073
C	-0.833037	3.819967	-1.621918
H	-1.475868	4.483116	-2.193157
C	-1.031499	2.421807	-1.698365
H	-1.846972	2.033035	-2.302170
C	-0.558777	0.080177	-0.823423
H	-1.126873	-0.261537	-1.697428
C	1.856294	-0.227018	-0.131692
C	0.699469	-0.750766	-0.699969
C	0.683312	-2.104559	-1.119954
N	1.694103	-2.935993	-1.021291
C	2.871751	-2.431115	-0.549151
C	3.980620	-3.316914	-0.518289
H	3.797427	-4.343592	-0.817448
C	5.230968	-2.872682	-0.158087
H	6.075806	-3.554971	-0.147824
C	5.419500	-1.509183	0.158021
C	4.354774	-0.633202	0.167003
C	3.033022	-1.065832	-0.135826
C	-1.458265	-0.132577	0.449612
H	-0.816969	0.121809	1.300874
C	-2.184923	-1.758202	2.043146

H	-1.286160	-1.652633	2.659378
H	-2.529780	-2.792033	2.152628
C	-3.292782	-0.745995	2.473597
H	-2.882995	0.027746	3.133656
H	-4.091152	-1.249600	3.030481
C	-3.856735	-0.095721	1.192460
H	-4.718119	0.537491	1.428102
C	-2.744053	0.741023	0.521521
H	-2.554594	1.657975	1.088771
H	-3.066765	1.062365	-0.474492
C	-4.287705	-1.245287	0.238243
H	-4.880338	-1.959354	0.824763
C	-2.954822	-1.936559	-0.217245
H	-3.060037	-3.026334	-0.194965
H	-2.725700	-1.667954	-1.253797
N	-1.811312	-1.558855	0.630292
C	-5.130570	-0.805943	-0.924897
H	-4.675746	-0.087363	-1.608652
C	-6.364842	-1.239747	-1.184951
H	-6.862850	-1.961404	-0.541413
H	-6.923807	-0.892191	-2.048651
C	1.829816	1.174617	0.353028
C	2.699003	1.699701	1.300378
C	2.750890	3.087355	1.562376
C	1.963742	3.965576	0.853678
H	2.051019	5.037845	1.006278
H	3.450969	3.458198	2.305074
H	-0.235906	-2.506824	-1.541236
H	3.358405	1.043017	1.853307
H	4.544734	0.412993	0.362472
H	6.416391	-1.140127	0.381171

Int.$C^{+\bullet}$

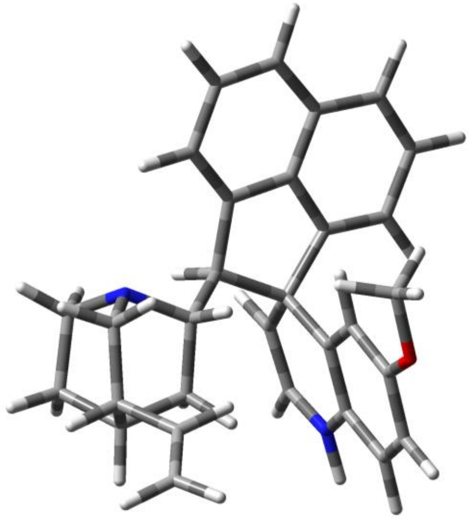

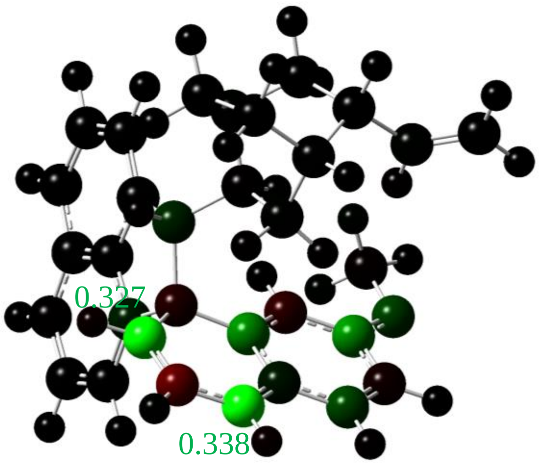

spin density:
Spin density represented by varying intensity of green color. A number for highest spin concentration on atom is given.

UM062X / CC-pVDZ
Charge = 1
Spin = Doublet
S^2 = 0.7726 (before annihilation), 0.7503 (after)
Solvation = SMD, tetrahydrofuran
Imaginary Freq = 0
Dipole Moment = 10.283622 Debye
Polarizability (α) = 491.41833 a.u.

Electronic Energy (EE) =	-1345.3756	Hartree
Zero-point Energy Correction =	0.543605	Hartree
Thermal Correction to Energy =	0.56861	Hartree
Thermal Correction to Enthalpy =	0.569555	Hartree
Thermal Correction to Free Energy =	0.48977	Hartree
EE + Zero-point Energy =	-1344.832	Hartree
EE + Thermal Energy Correction =	-1344.807	Hartree
EE + Thermal Enthalpy Correction =	-1344.8061	Hartree
EE + Thermal Free Energy Correction =	-1344.8859	Hartree

E (Thermal) = 356.808 kcal/mol
Heat Capacity (Cv) = 106.695 cal/mol-kelvin
Entropy (S) = 167.921 cal/mol-kelvin
Atomic coordinates:

C	-1.422697	-1.733739	0.040601
C	-2.616663	-0.979037	0.018734
C	-3.810900	-1.367098	0.656306
C	-3.770001	-2.595752	1.374515
H	-4.663068	-2.948939	1.893220
C	-2.602214	-3.328281	1.411818
H	-2.583173	-4.267175	1.968020
C	-1.410558	-2.916198	0.744266
H	-0.512779	-3.530024	0.789324
C	-0.379964	-1.039094	-0.821024
H	-0.317108	-1.581000	-1.778475
C	-1.042338	0.384911	-1.190126
C	-1.034516	0.575034	-2.674414
C	-0.491592	1.649812	-3.289811
N	0.098607	2.643766	-2.556479
C	0.166158	2.637451	-1.200526
C	0.814640	3.708810	-0.532386
H	1.251293	4.515435	-1.123240
C	0.891814	3.712399	0.832414

H	1.390764	4.514351	1.375552
C	0.302016	2.655346	1.575636
C	-0.353247	1.601309	0.916377
C	-0.402308	1.559142	-0.469464
O	0.425807	2.747462	2.894595
C	-0.125429	1.710523	3.708580
H	-1.214949	1.647494	3.573656
H	0.105760	1.991633	4.740414
H	0.343018	0.743400	3.472116
C	1.034955	-0.992618	-0.226706
H	0.960759	-0.573655	0.788144
C	2.015414	-2.949393	-1.296382
H	1.175411	-2.962761	-2.004585
H	2.275274	-3.998353	-1.095225
C	3.225680	-2.172936	-1.874818
H	3.062674	-1.926183	-2.934652
H	4.146000	-2.773255	-1.813958
C	3.389659	-0.888215	-1.056484
H	4.172835	-0.248953	-1.486984
C	2.043803	-0.149168	-1.048886
H	1.693842	-0.031851	-2.088063
H	2.155344	0.856184	-0.617800
C	3.780855	-1.281889	0.387340
H	4.757230	-1.788831	0.344838
C	2.690696	-2.282162	0.889889
H	3.112111	-3.292417	0.989799
H	2.316041	-1.978230	1.879771
N	1.551531	-2.360713	-0.030892
C	3.916010	-0.087230	1.289054
H	2.986226	0.439196	1.546615
C	5.068888	0.362333	1.787232
H	6.015480	-0.138414	1.561692
H	5.106718	1.239607	2.436750
C	-2.491383	0.223899	-0.699948
C	-3.569331	1.065312	-0.825320
C	-4.793279	0.684408	-0.205134
C	-4.919338	-0.485300	0.517760
H	-5.870602	-0.739968	0.988735
H	-5.654729	1.347095	-0.300597
H	-0.487715	1.791863	-4.367963
H	-3.502660	2.007400	-1.373073
H	-0.813906	0.796566	1.488280
H	-1.501754	-0.202425	-3.279746
H	0.509258	3.435663	-3.052294

Int.D$^{+\bullet}$

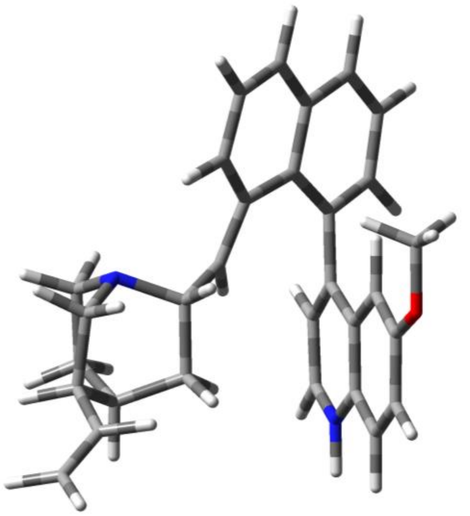

spin density:

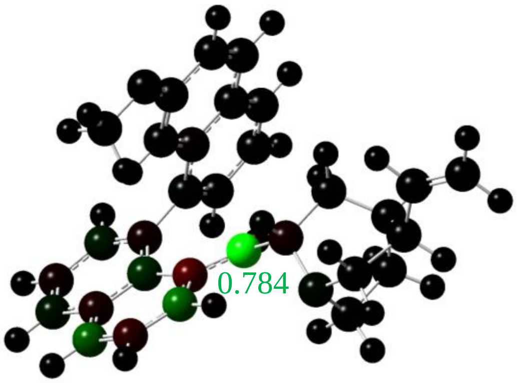

UM062X / CC-pVDZ
Charge = 1
Spin = Doublet
S^2 = 0.7848 (before annihilation), 0.7506 (after)
Solvation = SMD, tetrahydrofuran
Imaginary Freq = 0
Dipole Moment = 12.792503 Debye
Polarizability (α) = 476.326 a.u.

Electronic Energy (EE) =	-1345.367	Hartree
Zero-point Energy Correction =	0.535945	Hartree
Thermal Correction to Energy =	0.562745	Hartree
Thermal Correction to Enthalpy =	0.56369	Hartree
Thermal Correction to Free Energy =	0.477543	Hartree
EE + Zero-point Energy =	-1344.831	Hartree
EE + Thermal Energy Correction =	-1344.8042	Hartree
EE + Thermal Enthalpy Correction =	-1344.8033	Hartree
EE + Thermal Free Energy Correction =	-1344.8894	Hartree

E (Thermal) = 353.128 kcal/mol
Heat Capacity (Cv) = 110.219 cal/mol-kelvin
Entropy (S) = 181.31 cal/mol-kelvin

Atomic coordinates:

C	-0.896611	-1.566735	0.057271
C	-2.287620	-1.301454	-0.289789
C	-3.275789	-2.252911	0.106696
C	-2.921196	-3.353469	0.940127
H	-3.702593	-4.057094	1.231343
C	-1.625599	-3.506401	1.380255
H	-1.361752	-4.336181	2.037146
C	-0.623288	-2.628834	0.941104
H	0.416654	-2.826471	1.200560
C	0.182644	-0.829520	-0.488082
H	0.025208	-0.247513	-1.394312
C	-1.912330	1.078517	-1.243691
C	-1.788499	1.545150	-2.545056
C	-1.037785	2.688269	-2.816629
N	-0.444113	3.340911	-1.826392
C	-0.545135	2.969964	-0.513641
C	0.115210	3.725261	0.484545
H	0.698910	4.601181	0.197900
C	0.007694	3.339187	1.790786
H	0.504936	3.895828	2.585565
C	-0.768022	2.198453	2.154018
C	-1.420755	1.460401	1.186879

C	-1.306265	1.824582	-0.179037
O	-0.794319	1.932596	3.468188
C	-1.526871	0.787166	3.887112
H	-2.594414	0.890685	3.639008
H	-1.404440	0.731756	4.973512
H	-1.125796	-0.126664	3.420431
C	1.566822	-0.816451	0.091680
H	1.473943	-0.827250	1.190891
C	2.489687	-2.157747	-1.701066
H	1.507018	-2.355177	-2.153461
H	3.117751	-3.041118	-1.887140
C	3.138812	-0.879216	-2.294114
H	2.415384	-0.322370	-2.911146
H	3.992036	-1.136071	-2.939088
C	3.600490	0.006594	-1.129037
H	4.150983	0.881760	-1.502662
C	2.357687	0.451673	-0.348323
H	1.731952	1.090339	-0.994414
H	2.637132	1.057252	0.526201
C	4.525253	-0.839660	-0.225019
H	5.301226	-1.282796	-0.869660
C	3.642118	-1.971940	0.383002
H	4.137990	-2.946849	0.271810
H	3.488084	-1.800065	1.460264
N	2.320650	-2.049163	-0.246020
C	5.213992	-0.037248	0.839183
H	4.567171	0.425920	1.595304
C	6.534565	0.129728	0.929308
H	7.213871	-0.325426	0.202015
H	6.981513	0.721824	1.730982
C	-2.743411	-0.128735	-0.978259
C	-4.070874	-0.019008	-1.369183
C	-5.015777	-1.013092	-1.050805
C	-4.626864	-2.094817	-0.300514
H	-5.351126	-2.852652	0.003705
H	-6.052100	-0.895839	-1.368063
H	-0.894116	3.082332	-3.821128
H	-4.396613	0.882229	-1.891304
H	-2.022969	0.594086	1.449054
H	-2.244881	1.006715	-3.373637
H	0.114131	4.168378	-2.046609

Int.Fr$^{+\bullet}$

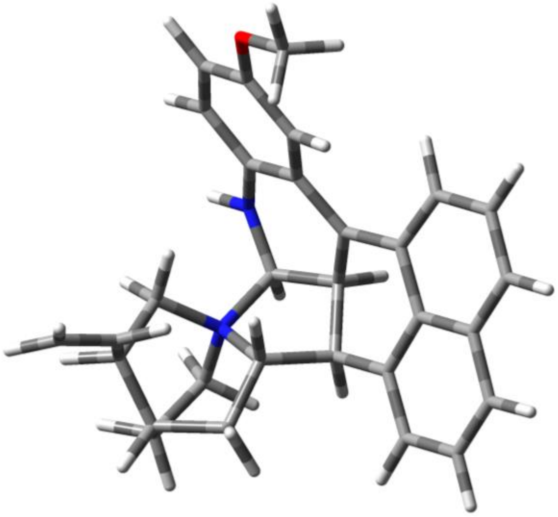

spin density:

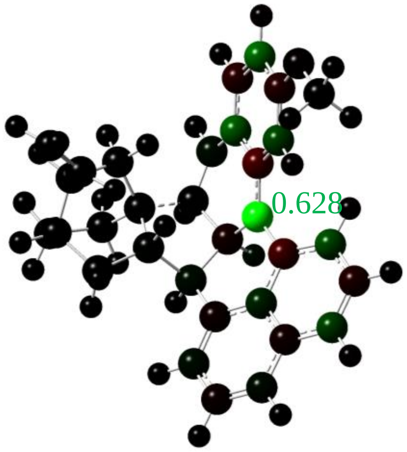

UM062X / CC-pVDZ
Charge = 1
Spin = Doublet
S^2 = 0.7783 (before annihilation), 0.7506 (after)
Solvation = SMD, tetrahydrofuran
Imaginary Freq = 0
Dipole Moment = 10.879541 Debye
Polarizability (α) = 479.08633 a.u.

Electronic Energy (EE) =	-1345.4134	Hartree
Zero-point Energy Correction =	0.541524	Hartree
Thermal Correction to Energy =	0.566365	Hartree
Thermal Correction to Enthalpy =	0.567309	Hartree
Thermal Correction to Free Energy =	0.487913	Hartree
EE + Zero-point Energy =	-1344.8719	Hartree
EE + Thermal Energy Correction =	-1344.847	Hartree
EE + Thermal Enthalpy Correction =	-1344.8461	Hartree
EE + Thermal Free Energy Correction =	-1344.9255	Hartree

E (Thermal) = 355.399 kcal/mol
Heat Capacity (Cv) = 106.698 cal/mol-kelvin
Entropy (S) = 167.104 cal/mol-kelvin

Atomic coordinates:

C	0.345424	-2.645235	-0.283029
C	1.690283	-2.194384	-0.141201
C	2.662101	-3.087262	0.395603
C	2.262439	-4.385300	0.814133
H	3.017780	-5.055303	1.229033
C	0.955639	-4.788710	0.690647
H	0.653449	-5.787388	1.008028
C	-0.002360	-3.916018	0.124993
H	-1.029838	-4.260125	-0.010757
C	-0.679515	-1.750177	-0.929987
H	-1.326926	-2.372477	-1.566861
C	1.058307	0.053629	-1.009518
C	-0.063790	-0.615744	-1.768827
C	-1.241440	0.308844	-2.072028
N	-0.883295	1.647273	-2.266122
C	0.081018	2.261500	-1.468688
C	0.086557	3.653260	-1.335416
H	-0.661701	4.238661	-1.873558
C	1.012804	4.284368	-0.522447
H	1.023185	5.368717	-0.412027
C	1.938198	3.516142	0.212941
C	1.951498	2.137597	0.083816

C	1.053303	1.466349	-0.798885
O	2.764178	4.220818	1.027369
C	3.713055	3.485694	1.776838
H	4.390989	2.916750	1.119266
H	4.292337	4.222971	2.343943
H	3.221873	2.791801	2.478505
C	-1.550231	-0.956202	0.040631
H	-0.880747	-0.341323	0.659786
C	-2.621768	1.230450	-0.031574
H	-1.695011	1.747309	0.244581
H	-3.228458	1.892954	-0.662105
C	-3.396952	0.747415	1.230252
H	-4.322290	1.336783	1.298160
C	-3.811553	-0.727815	1.018733
H	-4.469961	-1.040494	1.838137
C	-2.582165	-1.648683	0.928637
H	-2.136963	-1.838095	1.913802
H	-2.883516	-2.618563	0.504022
C	-4.541028	-0.796897	-0.323088
H	-5.337830	-0.041620	-0.361037
C	-3.511708	-0.551666	-1.434561
H	-3.865446	0.158292	-2.192671
H	-3.215456	-1.476381	-1.944915
N	-2.252079	0.040895	-0.861269
C	2.080772	-0.855528	-0.517458
C	3.427265	-0.511430	-0.423329
C	4.387838	-1.413201	0.079515
C	4.017015	-2.668508	0.500326
H	4.752077	-3.365879	0.905965
H	5.432337	-1.103037	0.129580
H	-1.804545	-0.048299	-2.938538
H	2.637222	1.543111	0.680305
H	3.756573	0.466118	-0.775081
H	-5.001676	-1.782897	-0.466869
C	-2.619076	0.964158	2.501368
C	-3.103535	1.616731	3.557634
H	-1.604376	0.550727	2.548925
H	-2.514026	1.739697	4.468356
H	-4.108635	2.048950	3.544925
H	0.325627	-1.011907	-2.719395
H	-1.572219	2.256983	-2.694894

Int.Es$^{+\bullet}$

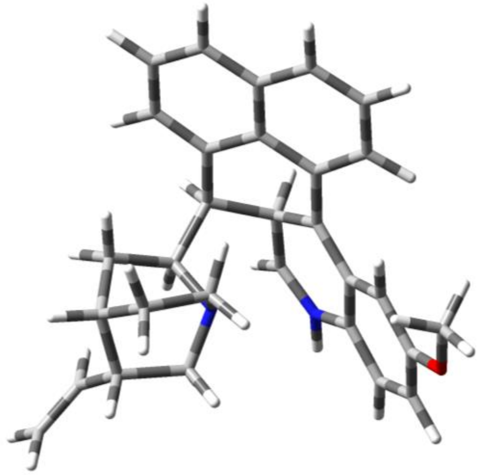

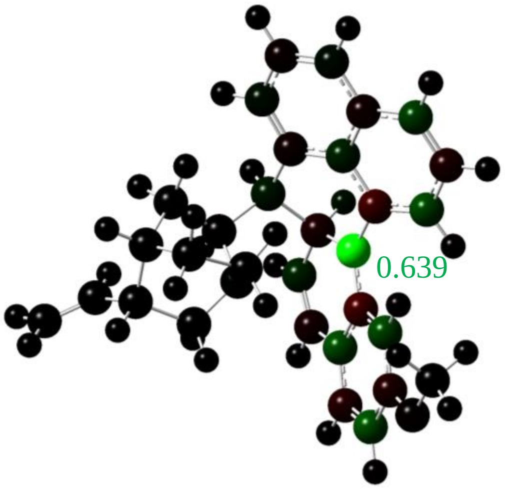

spin density:

UM062X / CC-pVDZ
Charge = 1
Spin = Doublet
S^2 = 0.7767 (before annihilation), 0.7506 (after)
Solvation = SMD, tetrahydrofuran
Imaginary Freq = 0
Dipole Moment = 7.924133 Debye
Polarizability (α) = 486.785 a.u.

Electronic Energy (EE) =	-1345.379467	Hartree
Zero-point Energy Correction =	0.538408	Hartree
Thermal Correction to Energy =	0.564119	Hartree
Thermal Correction to Enthalpy =	0.565063	Hartree
Thermal Correction to Free Energy =	0.483052	Hartree
EE + Zero-point Energy =	-1344.84106	Hartree
EE + Thermal Energy Correction =	-1344.81535	Hartree
EE + Thermal Enthalpy Correction =	-1344.8144	Hartree
EE + Thermal Free Energy Correction =	-1344.8964	Hartree

E (Thermal) = 353.99 kcal/mol
Heat Capacity (Cv) = 107.997 cal/mol-kelvin
Entropy (S) = 172.607 cal/mol-kelvin

Atomic coordinates:

C	0.233460	2.497822	0.675935
C	-1.143734	2.391236	0.310067
C	-1.797263	3.516648	-0.271471
C	-1.071081	4.718269	-0.485796
H	-1.586396	5.567553	-0.937980
C	0.248212	4.806304	-0.116694
H	0.803785	5.732808	-0.265733
C	0.890490	3.696511	0.477256
H	1.929833	3.791360	0.798849
C	0.963773	1.354153	1.358426
H	1.557506	1.814749	2.162749
C	-1.200461	0.042787	1.096433
C	-0.092998	0.433147	2.039119
C	0.541860	-0.712282	2.724091
N	0.190077	-1.929923	2.495396
C	-0.752812	-2.329842	1.534417
C	-0.928747	-3.692818	1.303624
H	-0.371107	-4.418401	1.898887
C	-1.797913	-4.112999	0.314910
H	-1.957558	-5.170260	0.107469
C	-2.474070	-3.152594	-0.467219
C	-2.299683	-1.790358	-0.232108
C	-1.448709	-1.334134	0.804099

O	-3.265520	-3.654100	-1.432715
C	-3.967715	-2.736882	-2.259253
H	-4.644783	-2.103817	-1.664050
H	-4.554331	-3.346752	-2.954305
H	-3.271153	-2.100967	-2.827496
C	1.978261	0.501207	0.542951
H	2.594407	0.022387	1.323095
C	0.832762	-0.206883	-1.472152
H	0.141283	0.631835	-1.310941
H	0.242310	-1.039296	-1.884564
C	1.983604	0.203462	-2.425955
H	2.135371	-0.552224	-3.211566
C	3.257851	0.338156	-1.582811
H	4.079064	0.756226	-2.180856
C	2.933581	1.266957	-0.399356
H	2.461259	2.173819	-0.803170
H	3.839708	1.579925	0.139970
C	3.642555	-1.076718	-1.091800
H	3.870174	-1.688254	-1.978439
C	2.380080	-1.654470	-0.373381
H	1.929381	-2.460541	-0.970826
H	2.654355	-2.080147	0.605865
N	1.353579	-0.628705	-0.162404
C	-1.891080	1.169167	0.501134
C	-3.241733	1.145711	0.165779
C	-3.878737	2.270424	-0.396897
C	-3.172277	3.427094	-0.624605
H	-3.655677	4.300404	-1.066206
H	-4.940317	2.215964	-0.640269
H	1.310822	-0.551060	3.482456
H	-2.787809	-1.061850	-0.872086
H	-3.835265	0.258143	0.383554
H	-0.525270	1.051104	2.851769
H	0.647106	-2.677622	3.025225
H	1.752813	1.157193	-2.922760
C	4.858431	-1.076291	-0.209415
C	6.028936	-1.627177	-0.534914
H	4.757249	-0.587802	0.768674
H	6.883493	-1.596602	0.144590
H	6.169257	-2.128317	-1.497627

Int.B•

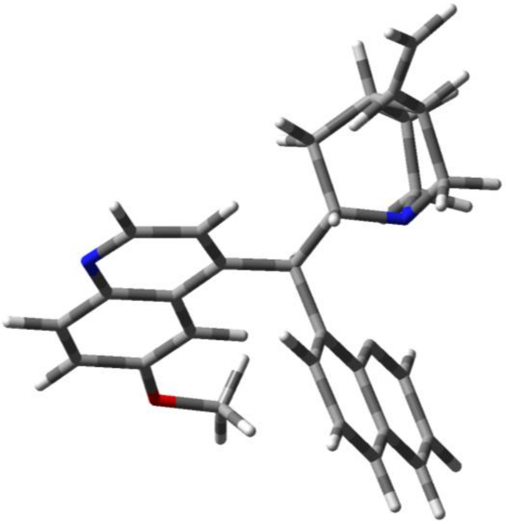

UM06-2X / CC-pVDZ
Spin = Doublet
Solvation = SMD, tetrahydrofuran
Imaginary Freq = 0
Dipole Moment = 4.8334622 Debye
Polarizability (α) = 453.14867 a.u.

Electronic Energy (EE) =	-1344.8873	Hartree
Zero-point Energy Correction =	0.523162	Hartree
Thermal Correction to Energy =	0.549665	Hartree
Thermal Correction to Enthalpy =	0.550609	Hartree
Thermal Correction to Free Energy =	0.465724	Hartree
EE + Zero-point Energy =	-1344.3641	Hartree
EE + Thermal Energy Correction =	-1344.3376	Hartree
EE + Thermal Enthalpy Correction =	-1344.3367	Hartree
EE + Thermal Free Energy Correction =	-1344.4215	Hartree

E (Thermal) = 344.92 kcal/mol
Heat Capacity (Cv) = 108.739 cal/mol-kelvin
Entropy (S) = 178.656 cal/mol-kelvin

Atomic coordinates:

C	-0.312700	-0.275551	0.018092
H	0.012156	0.003252	-0.995957
C	0.289582	-1.638610	0.313204
C	-0.400728	-2.646944	0.948111
H	-1.436406	-2.517165	1.261326
C	0.229521	-3.889313	1.206971
H	-0.337174	-4.679846	1.707672
N	1.469586	-4.169934	0.879078
C	2.186191	-3.185214	0.264730
C	3.539764	-3.468342	-0.078890
H	3.922667	-4.462486	0.155111
C	4.329037	-2.522059	-0.670939
H	5.368776	-2.723513	-0.931423
C	3.802972	-1.229660	-0.954650
C	2.491646	-0.928342	-0.652546
H	2.098111	0.061468	-0.867651
C	1.654924	-1.900937	-0.036798
O	4.676717	-0.367285	-1.519224
C	4.211073	0.949485	-1.765717
H	3.373027	0.948891	-2.481923
H	5.057432	1.496889	-2.195253
H	3.890493	1.438960	-0.831107
C	-1.854076	-0.264016	0.018506

H	-2.180201	-0.515737	1.041800
C	-2.298207	1.441909	-1.653301
H	-1.259323	1.326212	-1.993167
H	-2.544196	2.509530	-1.751121
C	-3.254042	0.561111	-2.497216
H	-2.736996	0.161015	-3.382237
H	-4.118813	1.141660	-2.853952
C	-3.731663	-0.585048	-1.600500
H	-4.316138	-1.315731	-2.177018
C	-2.494787	-1.257796	-0.984841
H	-1.782424	-1.509252	-1.788926
H	-2.766569	-2.201674	-0.492848
C	-4.618590	0.026778	-0.490457
H	-5.496401	0.482135	-0.975330
C	-3.764075	1.140457	0.199132
H	-4.161587	2.135507	-0.047944
H	-3.800639	1.031503	1.294197
N	-2.361651	1.099166	-0.224026
C	-5.112540	-1.001513	0.486721
H	-4.357371	-1.459098	1.139333
C	-6.386228	-1.377647	0.614019
H	-7.170300	-0.938787	-0.010856
H	-6.689192	-2.131897	1.343621
C	0.232809	0.763963	0.992261
C	0.755686	2.005147	0.514567
C	0.217452	0.536471	2.350537
C	0.843519	2.358253	-0.844509
C	1.260685	2.979552	1.434258
C	0.697876	1.502312	3.269445
H	-0.165626	-0.412835	2.731715
C	1.348620	3.512044	-1.351775
C	1.800446	4.199429	0.932847
C	1.211733	2.695657	2.826030
H	0.664178	1.283414	4.337634
C	1.848406	4.463971	-0.414741
H	1.377420	3.716043	-2.423656
H	2.180471	4.928909	1.650562
H	1.593381	3.438755	3.528880
H	2.266127	5.404268	-0.778357

Int.C•

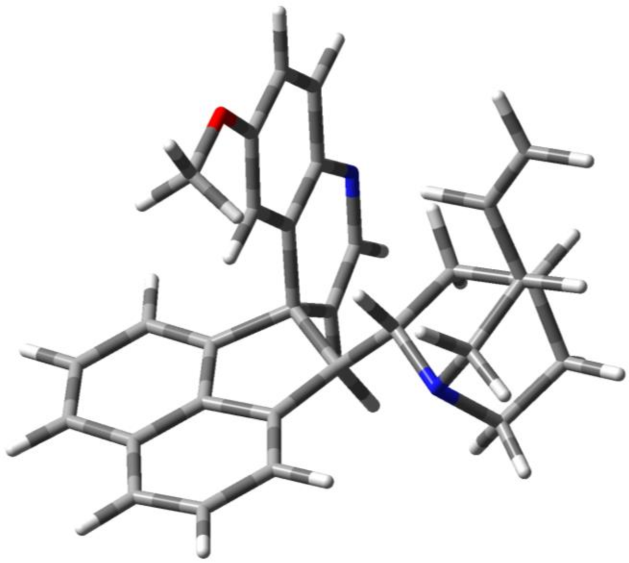

UM06-2X / CC-pVDZ
Spin = Doublet
Solvation = SMD, tetrahydrofuran
Imaginary Freq = 0
Dipole Moment = 3.6779371 Debye
Polarizability (α) = 465.42367 a.u.

Electronic Energy (EE) =	-1344.9213	Hartree
Zero-point Energy Correction =	0.522767	Hartree
Thermal Correction to Energy =	0.548805	Hartree
Thermal Correction to Enthalpy =	0.549749	Hartree
Thermal Correction to Free Energy =	0.465167	Hartree
EE + Zero-point Energy =	-1344.3986	Hartree
EE + Thermal Energy Correction =	-1344.3725	Hartree
EE + Thermal Enthalpy Correction =	-1344.3716	Hartree
EE + Thermal Free Energy Correction =	-1344.4562	Hartree

E (Thermal) = 344.38 kcal/mol
Heat Capacity (Cv) = 108.035 cal/mol-kelvin
Entropy (S) = 178.017 cal/mol-kelvin
Atomic coordinates:

C	-1.351674	-1.734591	0.076568
C	-2.574550	-1.029034	0.000751
C	-3.764197	-1.433903	0.636964
C	-3.686279	-2.620942	1.420076
H	-4.573536	-2.984029	1.942347
C	-2.490119	-3.300864	1.515511
H	-2.443515	-4.207439	2.121944
C	-1.305208	-2.877367	0.842583
H	-0.385649	-3.453771	0.928756
C	-0.330615	-1.043525	-0.814409
H	-0.264503	-1.621944	-1.750999
C	-1.023889	0.344926	-1.230315
C	-0.966055	0.539173	-2.711942
C	-0.437208	1.662368	-3.297350
N	0.097814	2.704846	-2.631992
C	0.079614	2.638286	-1.260197
C	0.644797	3.731389	-0.555441
H	1.052701	4.553050	-1.145956
C	0.686855	3.756209	0.819584
H	1.127199	4.590938	1.365587
C	0.140135	2.682765	1.554029
C	-0.435199	1.600429	0.882261

C	-0.449317	1.556847	-0.514395
O	0.219837	2.788025	2.895985
C	-0.290421	1.714297	3.670766
H	-1.369938	1.576219	3.501775
H	-0.117372	1.987414	4.717391
H	0.239261	0.774452	3.445450
C	1.085612	-0.953081	-0.230388
H	1.003783	-0.514012	0.775273
C	2.122579	-2.900413	-1.263897
H	1.287795	-2.936669	-1.977527
H	2.402101	-3.942352	-1.050666
C	3.320195	-2.106186	-1.844996
H	3.162558	-1.884884	-2.911210
H	4.254547	-2.682591	-1.762131
C	3.441215	-0.801341	-1.050977
H	4.209186	-0.148599	-1.488819
C	2.075205	-0.100925	-1.066997
H	1.729414	-0.007506	-2.110381
H	2.151300	0.916011	-0.657737
C	3.835578	-1.159712	0.401284
H	4.826552	-1.639519	0.373949
C	2.771293	-2.183372	0.913318
H	3.221822	-3.179815	1.029044
H	2.385336	-1.876861	1.898175
N	1.638350	-2.306083	-0.009074
C	3.930942	0.050804	1.286439
H	2.988669	0.568766	1.511848
C	5.064959	0.521630	1.808079
H	6.023701	0.030873	1.613494
H	5.074841	1.409176	2.444586
C	-2.477205	0.140741	-0.775578
C	-3.589013	0.925373	-0.964817
C	-4.810181	0.524848	-0.352133
C	-4.905424	-0.609091	0.430757
H	-5.855807	-0.878372	0.895672
H	-5.696430	1.144068	-0.501351
H	-0.427024	1.742492	-4.388098
H	-3.546613	1.841138	-1.558020
H	-0.869641	0.771509	1.441508
H	-1.377135	-0.257729	-3.335172

Int.D•

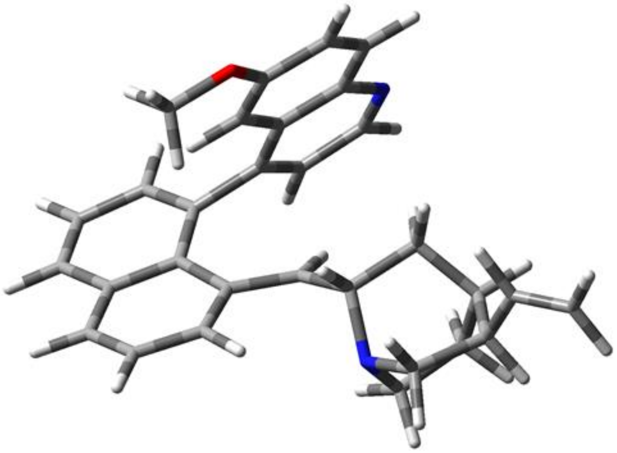

UM06-2X / CC-pVDZ
Spin = Doublet
Solvation = SMD, tetrahydrofuran
Imaginary Freq = 0
Dipole Moment = 3.1368911 Debye
Polarizability (α) = 473.279 a.u.

Electronic Energy (EE) =	-1344.919 Hartree
Zero-point Energy Correction =	0.521478 Hartree
Thermal Correction to Energy =	0.548332 Hartree
Thermal Correction to Enthalpy =	0.549277 Hartree
Thermal Correction to Free Energy =	0.462125 Hartree
EE + Zero-point Energy =	-1344.3976 Hartree
EE + Thermal Energy Correction =	-1344.3707 Hartree
EE + Thermal Enthalpy Correction =	-1344.3698 Hartree
EE + Thermal Free Energy Correction =	-1344.4569 Hartree

E (Thermal) = 344.084 kcal/mol
Heat Capacity (Cv) = 109.691 cal/mol-kelvin
Entropy (S) = 183.425 cal/mol-kelvin

Atomic coordinates:

C	-0.885593	-1.553985	0.110803
C	-2.277366	-1.315951	-0.268220
C	-3.256726	-2.271969	0.145112
C	-2.898786	-3.346996	1.010777
H	-3.675971	-4.051163	1.312551
C	-1.607193	-3.473952	1.469761
H	-1.341265	-4.283810	2.150637
C	-0.611833	-2.592917	1.023011
H	0.426432	-2.770205	1.302712
C	0.197432	-0.818138	-0.427992
H	0.044531	-0.230466	-1.329390
C	-1.905054	1.031987	-1.310040
C	-1.716270	1.422616	-2.618075
C	-0.931874	2.564847	-2.907647
N	-0.354604	3.311245	-1.992422
C	-0.552330	2.964368	-0.687938
C	0.071863	3.765216	0.312513
H	0.664533	4.618558	-0.019343
C	-0.066540	3.465784	1.638818
H	0.410036	4.066541	2.414485
C	-0.851179	2.347131	2.044426
C	-1.476810	1.556598	1.104086
C	-1.326565	1.843564	-0.281015
O	-0.913571	2.151783	3.379925
C	-1.648307	1.026828	3.832208
H	-2.710630	1.104266	3.550674

H	-1.559701	1.023435	4.924154
H	-1.231965	0.090447	3.425547
C	1.576151	-0.794196	0.165606
H	1.473348	-0.785770	1.263936
C	2.495776	-2.172997	-1.599556
H	1.513669	-2.398084	-2.040116
H	3.138634	-3.049501	-1.769087
C	3.116800	-0.897627	-2.227292
H	2.371555	-0.354992	-2.830579
H	3.954573	-1.156188	-2.891992
C	3.599701	0.007977	-1.086153
H	4.141329	0.877317	-1.485161
C	2.370984	0.465488	-0.290613
H	1.743001	1.108013	-0.928897
H	2.667168	1.072369	0.577368
C	4.540007	-0.824284	-0.185545
H	5.296395	-1.291723	-0.836809
C	3.660727	-1.931945	0.471024
H	4.157542	-2.910256	0.395714
H	3.513851	-1.717481	1.541791
N	2.335349	-2.032517	-0.145866
C	5.261643	-0.004535	0.842337
H	4.635831	0.501947	1.588205
C	6.587112	0.131497	0.912269
H	7.246101	-0.364367	0.192833
H	7.058114	0.739816	1.687636
C	-2.740096	-0.166167	-0.992014
C	-4.069239	-0.084775	-1.381806
C	-5.004810	-1.083644	-1.048790
C	-4.607307	-2.144613	-0.274293
H	-5.322607	-2.905726	0.043330
H	-6.041167	-0.986309	-1.374040
H	-0.773569	2.855292	-3.950370
H	-4.400387	0.800289	-1.927540
H	-2.082935	0.703700	1.401518
H	-2.139016	0.837419	-3.435709

Int.Er•

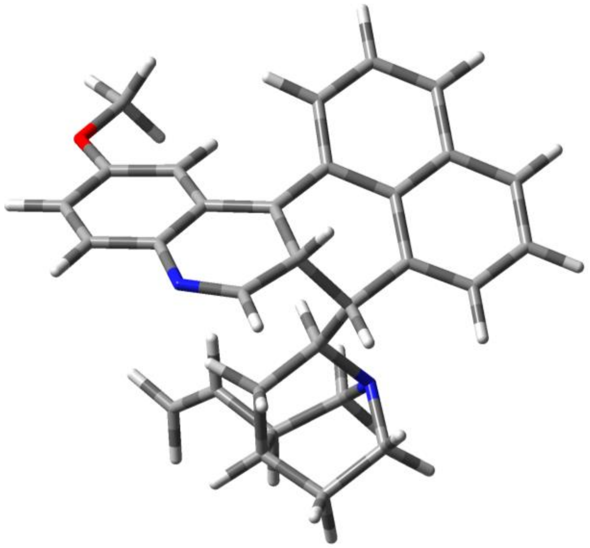

UM06-2X / CC-pVDZ
Spin = Doublet
Solvation = SMD, tetrahydrofuran
Imaginary Freq = 0
Dipole Moment = 3.2173176 Debye
Polarizability (α) = 487.05733 a.u.

Electronic Energy (EE) =	-1344.9339	Hartree
Zero-point Energy Correction =	0.524405	Hartree
Thermal Correction to Energy =	0.549945	Hartree
Thermal Correction to Enthalpy =	0.550889	Hartree
Thermal Correction to Free Energy =	0.468881	Hartree
EE + Zero-point Energy =	-1344.4094	Hartree
EE + Thermal Energy Correction =	-1344.3839	Hartree
EE + Thermal Enthalpy Correction =	-1344.383	Hartree
EE + Thermal Free Energy Correction =	-1344.465	Hartree

E (Thermal) = 345.096 kcal/mol
Heat Capacity (Cv) = 107.158 cal/mol-kelvin
Entropy (S) = 172.601 cal/mol-kelvin
Atomic coordinates:

C	-2.259916	-1.165616	0.559022
C	-1.437002	-2.229916	0.076580
C	-2.044898	-3.318555	-0.613895
C	-3.454810	-3.335181	-0.788981
H	-3.906744	-4.177652	-1.316118
C	-4.228490	-2.309237	-0.303194
H	-5.311169	-2.325236	-0.436562
C	-3.624043	-1.220403	0.365737
H	-4.245655	-0.402661	0.735541
C	-1.614889	0.024403	1.239965
H	-2.324862	0.452627	1.965019
C	0.572667	-1.141801	1.042313
C	-0.381962	-0.516902	2.016593
C	0.271747	0.437366	2.968284
N	1.499512	0.793570	2.962770
C	2.316154	0.342751	1.909072
C	3.602241	0.887328	1.833019
H	3.902204	1.589412	2.612163
C	4.464331	0.563697	0.798571
H	5.465052	0.990366	0.730968
C	4.032445	-0.318365	-0.212594
C	2.766388	-0.887528	-0.154050
C	1.885350	-0.594780	0.922866

O	4.922757	-0.543761	-1.207508
C	4.527541	-1.415325	-2.251575
H	4.297891	-2.422795	-1.868012
H	5.379655	-1.474052	-2.937965
H	3.650872	-1.023067	-2.791777
C	-1.229418	1.102275	0.201712
H	-0.523044	0.621850	-0.495816
C	-3.231672	2.461571	0.084790
H	-3.528499	2.010691	1.043082
H	-4.151747	2.599672	-0.502257
C	-2.501485	3.810138	0.311644
H	-2.627445	4.154065	1.349274
H	-2.904261	4.594679	-0.347571
C	-1.016708	3.583990	0.004668
H	-0.421521	4.466497	0.277623
C	-0.554568	2.358028	0.808143
H	-0.860283	2.496993	1.858529
H	0.540557	2.258409	0.801480
C	-0.883611	3.316266	-1.512057
H	-1.207428	4.224235	-2.045047
C	-1.861165	2.140232	-1.840664
H	-2.719612	2.508265	-2.421640
H	-1.353068	1.375271	-2.448036
N	-2.379485	1.500474	-0.628541
C	0.531526	3.020510	-1.921781
H	0.968158	2.085964	-1.544808
C	1.273520	3.806431	-2.703959
H	0.875319	4.743379	-3.105889
H	2.299840	3.542195	-2.968392
C	0.000267	-2.215988	0.254009
C	0.744385	-3.280854	-0.250324
C	0.130434	-4.347954	-0.937737
C	-1.232340	-4.371214	-1.119918
H	-1.713276	-5.198091	-1.645900
H	0.748147	-5.167277	-1.308200
H	-0.362146	0.829791	3.774634
H	1.817120	-3.323983	-0.063455
H	2.421118	-1.532591	-0.957160
H	-0.799509	-1.317370	2.659197
