# Supplementary figures and images for: Stereoselective Domino Rearrangement peri-Annulation of Cinchona Alkaloid Derivatives with 8-Bromo-1-naphthyl Grignard
Source: J Org Chem. 2022 Aug 23;87(17):11602–7. doi: 10.1021/acs.joc.2c01249 (PMC9442652; doi:10.1021/acs.joc.2c01249)

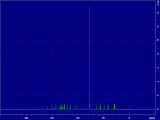

Supplement: Supplementary file 2 — jo2c01249_si_002.zip [file jo2c01249_si_002.zip › 1/13C(a)/pdata/1/thumb.png]

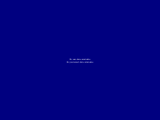

Supplement: Supplementary file 2 — jo2c01249_si_002.zip [file jo2c01249_si_002.zip › 1/13C(benzene)/pdata/1/thumb.png]

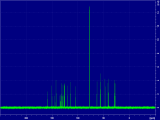

Supplement: Supplementary file 2 — jo2c01249_si_002.zip [file jo2c01249_si_002.zip › 1/13C/pdata/1/thumb.png]

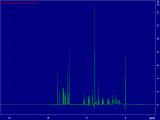

Supplement: Supplementary file 2 — jo2c01249_si_002.zip [file jo2c01249_si_002.zip › 1/1H(a)/pdata/1/thumb.png]

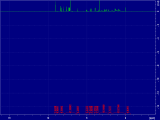

Supplement: Supplementary file 2 — jo2c01249_si_002.zip [file jo2c01249_si_002.zip › 1/1H(benzene)/pdata/1/thumb.png]

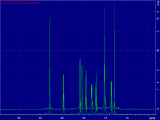

Supplement: Supplementary file 2 — jo2c01249_si_002.zip [file jo2c01249_si_002.zip › 1/1H/pdata/1/thumb.png]

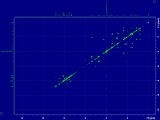

Supplement: Supplementary file 2 — jo2c01249_si_002.zip [file jo2c01249_si_002.zip › 1/COSY(a)/pdata/1/thumb.png]
